# Building machine learning prediction models for well-being using predictors from the exposome and genome in a population cohort

**DOI:** 10.1038/s44220-024-00294-2

**Published:** 2024-08-14

**Authors:** Dirk H. M. Pelt, Philippe C. Habets, Christiaan H. Vinkers, Lannie Ligthart, Catharina E. M. van Beijsterveldt, René Pool, Meike Bartels

**Affiliations:** 1 Department of Biological Psychology, Faculty of Behavioral and Movement Sciences, Vrije Universiteit Amsterdam, Amsterdam, The Netherlands; 2 Amsterdam Public Health Research Institute, Amsterdam University Medical Center, Amsterdam, The Netherlands; 3 Department of Psychiatry and Anatomy and Neurosciences, Amsterdam University Medical Center location Vrije Universiteit Amsterdam, Amsterdam, The Netherlands; 4 Amsterdam Neuroscience, Mood, Anxiety, Psychosis, Sleep and Stress Program, Amsterdam, The Netherlands; 5 GGZ inGeest Mental Health Care, Amsterdam, The Netherlands

**Keywords:** Psychology, Behavioural genetics, Heritable quantitative trait, Quality of life

## Abstract

Effective personalized well-being interventions require the ability to predict who will thrive or not, and the understanding of underlying mechanisms. Here, using longitudinal data of a large population cohort (the Netherlands Twin Register, collected 1991–2022), we aim to build machine learning prediction models for adult well-being from the exposome and genome, and identify the most predictive factors (*N* between 702 and 5874). The specific exposome was captured by parent and self-reports of psychosocial factors from childhood to adulthood, the genome was described by polygenic scores, and the general exposome was captured by linkage of participants’ postal codes to objective, registry-based exposures. Not the genome (*R*^2^ = −0.007 [−0.026–0.010]), but the general exposome (*R*^2^ = 0.047 [0.015–0.076]) and especially the specific exposome (*R*^2^ = 0.702 [0.637–0.753]) were predictive of well-being in an independent test set. Adding the genome (*P* = 0.334) and general exposome (*P* = 0.695) independently or jointly (*P* = 0.029) beyond the specific exposome did not improve prediction. Risk/protective factors such as optimism, personality, social support and neighborhood housing characteristics were most predictive. Our findings highlight the importance of longitudinal monitoring and promises of different data modalities for well-being prediction.

## Abstract (web summary)

Machine learning prediction models for adult well-being were built on longitudinal data from the Netherlands Twin Register population cohort. The exposome, but not the genome, predicted well-being in adulthood, with key factors including optimism, personality, social support and neighborhood housing characteristics.

## Main

Well-being is generally defined as a multidimensional concept encompassing not only the absence of negative aspects (such as illness or distress) but also the presence of positive elements, including a sense of fulfillment, productivity and overall flourishing^1^. Different conceptualizations of well-being exist, the most common distinction being made between subjective well-being and psychological well-being, also respectively known as hedonic and eudaimonic well-being^1–3^. The current study focuses, due to data availability, on subjective well-being, defined as the cognitive and affective evaluation of one’s life. The cognitive aspect is usually indicated by life satisfaction or quality of life, the affective aspect by positive affect or happiness and the absence of negative affect^2^. For brevity, we simply use the term well-being.

For the development of personalized well-being interventions, it is important to be able to predict who will thrive and to understand why this is the case. In the current study, we aim to develop optimal risk prediction models for well-being at the individual level and identify the factors driving these predictions^4^. Previous studies have provided possible risk and protective factors from the exposome and genome for well-being. The exposome is defined as all the environmental exposures one is exposed to during lifetime, and is increasingly being linked to health and well-being^5–9^. It consists of three interlinked and partly overlapping categories: the internal, specific external, and general external exposome^5^. In this Article, we define the specific (external) exposome as all lifestyle (for example, substance use, diet and medical information) and psychosocial factors related to (social) stress and support (for example, personality, life events, education and occupation). Related to this domain, we know that adult well-being often has its developmental origins in childhood and adolescence, as indicated by associations with childhood psychopathology^10,11^. In adolescence and adulthood, psychosocial factors such as personality traits^12^, social support^13,14^ and health indicators^4^ appear to be important. In addition, many environmental factors are associated with mental health, including socio-economic status (SES), childhood maltreatment, substance use and life events^15^. The general (external) exposome is defined here as the built, external environment in which an individual is embedded. From this domain, objective neighborhood characteristics such as urbanicity, air pollution, greenspace availability and SES indicators (for example, income, social security beneficiaries and housing stock value) have recently been associated with well-being and traits like depression^7,16–20^. Further, a recent data-driven, environment-wide association study revealed the importance of neighborhood safety for well-being, even after correcting for SES at the individual and neighborhood level^7^. Since subjective reports of one’s environment may be influenced by one’s mental health status^21^, the current study focuses on objective exposure measurements. Another reason for this focus relates to well-being policies: identifying modifiable neighborhood characteristics associated with individuals’ well-being allows for the formulation of targeted governmental policies. Finally, mental health traits (for example, depression, life satisfaction and positive affect) are partly driven by thousands of genetic variants with many small but relevant effects, many of which are shared across traits^22–25^. This is evidenced by high genetic correlations between depression, anxiety, personality traits and well-being, indicating a shared etiology^26,27^.

Aforementioned risk/protective factors are largely identified in cross-sectional studies associating well-being with one or more predictors following a pick-and-choose approach, providing group-level associations. Yet, finding an association with a certain risk/protective factor does not imply that it is useful to predict well-being at the individual level. Prediction, in this context, refers to the process of making forecasts on new, unseen data points based on patterns learned from a set of training data^28^. Associations may not translate to good predictions, for example due to low effect sizes or redundancy with respect to other more predictive variables^29^, but also given individual differences in pathways to well-being. Ultimately, personalized treatment and well-being interventions will depend on the ability to accurately predict at the individual level by pinpointing the risk/protective factors relevant to each individual.

The pick-and-choose approach further ignores well-being’s complexity of genetic, childhood, psychosocial and environmental factors interacting with each other^15,30^, while research on, for example, gene–environment interplay is abundant^15,31,32^. Abdellaoui and colleagues^33^, for example, showed that polygenic scores (PGSs) of educational attainment, a trait linked with well-being, were associated with the SES of one’s geographic location in the United Kingdom, as well as with social mobility (that is, migrating out of low SES regions). Genetic differences between people are thus correlated with environmental exposures, which may lead to differences in well-being levels.

Given its complexity, optimal well-being prediction will require broad inclusion of possible risk and protective factors from different data modalities; only analyzing them together will lead to the identification of the most relevant factors associated with well-being. In addition, optimal prediction requires the appropriate methods that can deal with the complex origins of well-being. Machine learning methods enable the inclusion of large numbers of variables (‘features’) from different data modalities, while accounting for their potential nonlinear interactions, consistent with the consensus that well-being results from complex interactions between developmental, social, psychological, genetic and environmental factors.

Previous studies, using a wide variety of linear (for example, support vector machines (SVM) and linear regression) and nonlinear (for example, extreme gradient boost and random forest (RF)) machine learning algorithms have shown multimodal models to outperform unimodal models in the prediction of resilience and depression-related phenotypes^29,34–38^. However, these studies focused on mental illness rather than on well-being. A recent study on well-being specifically found that expanding the set of features increased prediction considerably^4^, but was based on a single data modality (self-reported psychosocial predictors). Studies including multiple data modalities are often limited to clinical samples (for example, ref. ^29^), that is, when treatment is already sought, limiting their external/ecological validity and practical usefulness, especially for prevention. Machine learning prediction studies in population samples have largely failed to take an integrative approach using cross-sectional data, while lacking environmental exposures and/or genetic data^4,37–41^. These limitations may explain why predictive accuracies have not reached the standards needed for clinical use^42^.

**Extended Data Fig. 1:**
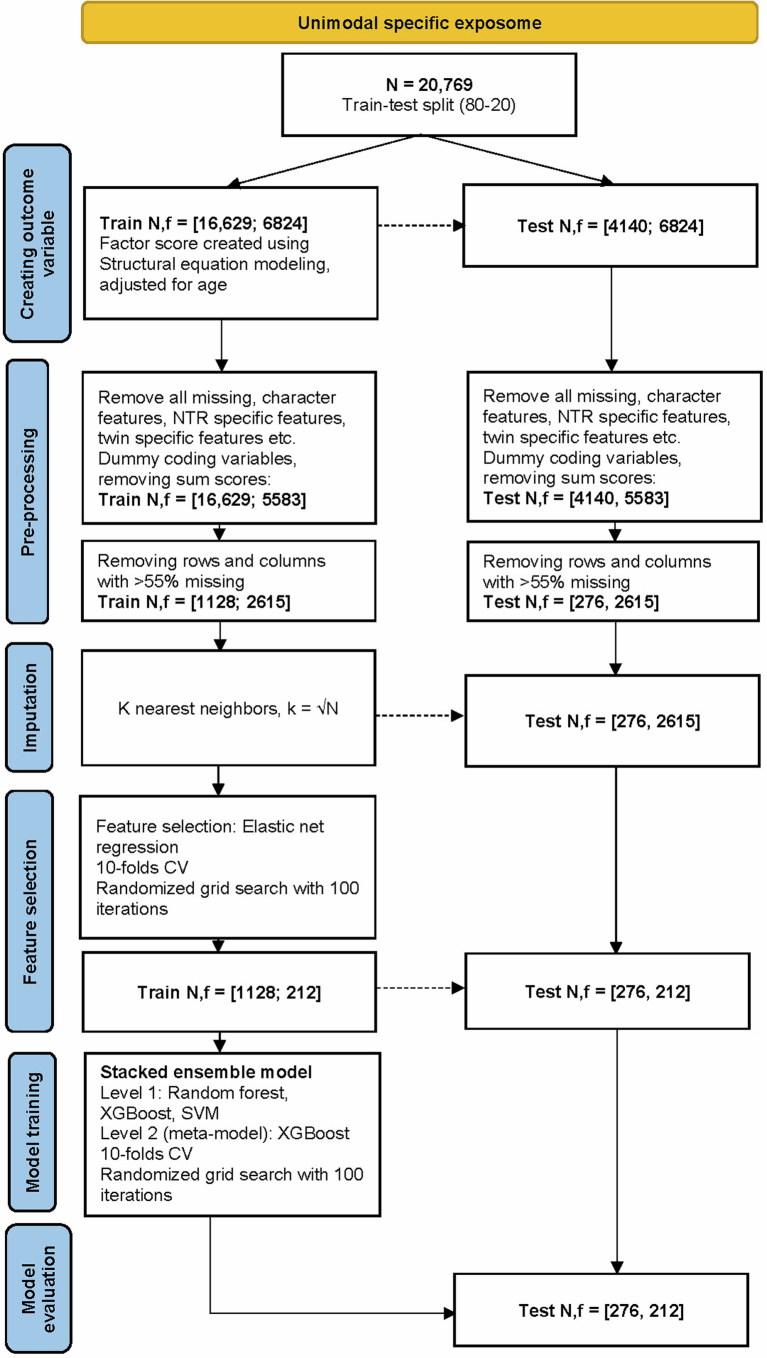
Flow chart of full data preparation and machine learning pipeline – unimodal specific exposome. *Note*. N,f = sample size, number of features, XGBoost = extreme gradient boost, SVM = support vector machine. Dotted lines represent transformations/selections in test set based on train set.

**Extended Data Fig. 2:**
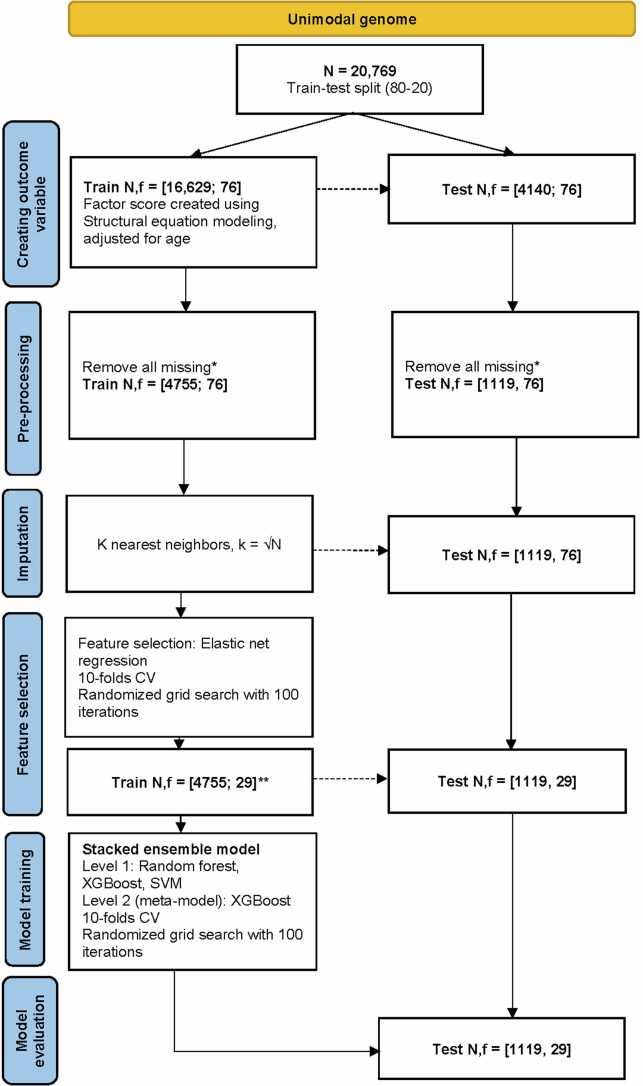
Flow chart of full data preparation and machine learning pipeline – unimodal genome. *Note*. N,f = sample size, number of features, XGBoost = extreme gradient boost, SVM = support vector machine. Dotted lines represent transformations/selections in test set based on train set. * Participants either had all or no genomic data available, ** 13 polygenic scores, 10 principal components, 6 platform dummies.

**Extended Data Fig. 3:**
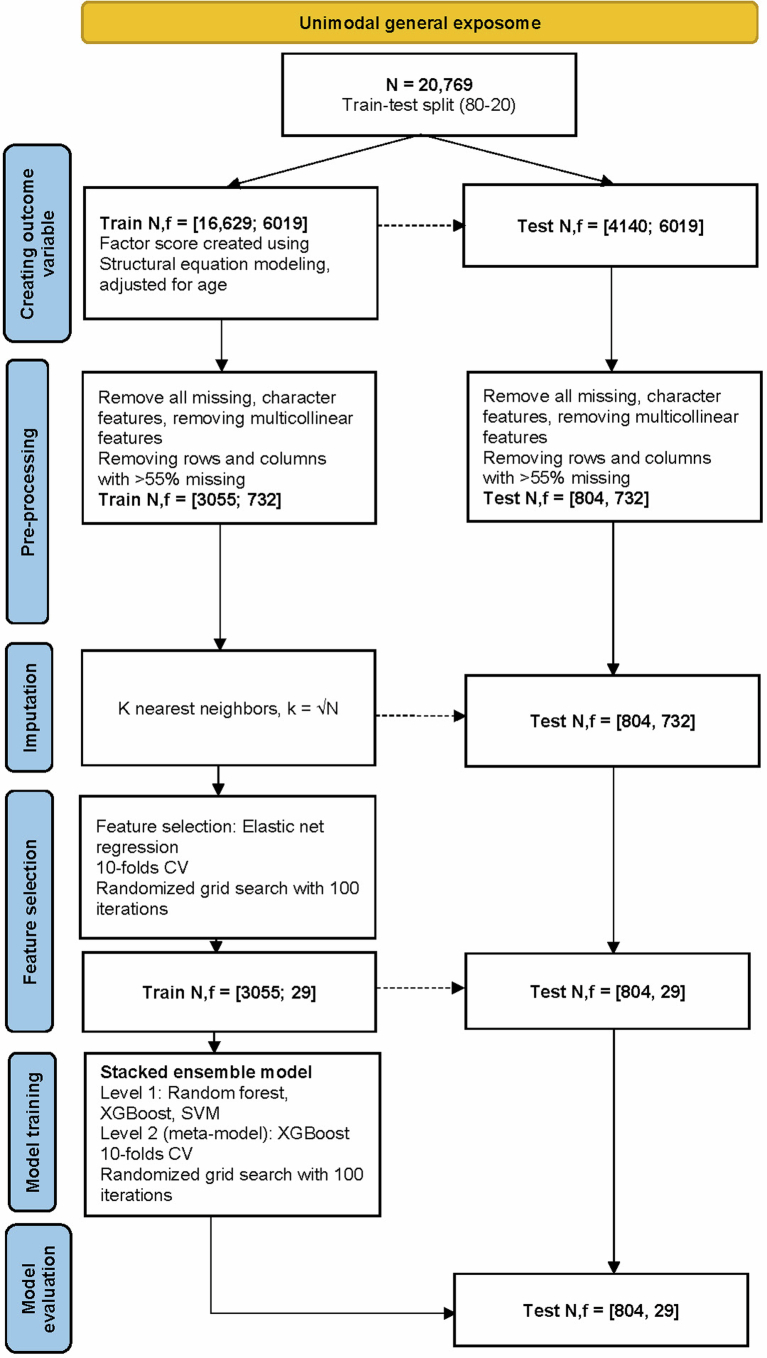
Flow chart of full data preparation and machine learning pipeline – unimodal general exposome. *Note*. N,f = sample size, number of features, XGBoost = extreme gradient boost, SVM = support vector machine. Dotted lines represent transformations/selections in test set based on train set.

In the present study, we address some of previous studies’ caveats by building prediction models for well-being using longitudinal objective environmental exposures (general exposome) and psychosocial factors (specific exposome), combined with genetic data (genome). We use data from a large population cohort, the Netherlands Twin Register (NTR), collected between 1991 and 2022, seven waves of the Young NTR (YNTR) collected around age 3, 5, 7, 10, 12, 14 and 16, and three adult waves collected in the Adult NTR (ANTR8 (2009–2012), ANTR10 (2013–2015) and ANTR14 (2019–2022); Table 1). We first train three machine learning algorithms, XGBoost (XGB), SVM and RF, and their predictions serve as inputs for a final XGB meta-model. The second aim is to identify the most predictive features of well-being using Shapley Additive Explanation (SHAP) values^43^, a method used to determine feature importance in predictive models. The outcome, well-being in adulthood, is based on multiple ratings of life satisfaction, happiness and quality of life. Our preprocessing and modeling pipeline is presented in Extended Data Figs. 1–3 and Supplementary Fig. 1.

**Table 1:** Demographic information

											Age					Background ^a^			
Dataset	*N*	%W	*N*_Train_	*N*_Test_	Gender	YNTR3	YNTR5	YNTR7	YNTR10	YNTR12	YNTR14	YNTR16	ANTR8	ANTR10	ANTR14	Dutch (%)	Non-Dutch (%)	No. Fam	
Specific exposome	1,404	71^b^	1,128	276	W	3.25 (0.22)	5.28 (0.31)	7.26 (0.24)	9.95 (0.3)	12.15 (0.37)	14.92 (0.59)	17.09 (0.57)	18.53 (0.74)	21.13 (2.11)	27.83 (2.3)	95 95	5 5	1,001	
						[2.9–4.3]	[4.7–7]	[6.8–8.8]	[9.4–12.3]	[11.5–13.5]	[13.2–18.4]	[15.7–18.7]	[18–22]	[18–26]	[23–33]				
					M	3.26 (0.21)	5.27 (0.25)	7.26 (0.21)	9.98 (0.3)	12.16 (0.35)	14.88 (0.61)	17.05 (0.54)	18.47 (0.69)	20.83 (2.04)	27.6 (2.3)				
						[2.9–3.9]	[4.8–6.7]	[6.8–8.2]	[9.4–11.1]	[11.5–13.3]	[13.2–16.2]	[16.1–18.8]	[18–20]	[18–26]	[24–33]				
Genome	5,874	66^b^	4,755	1119	W	3.29 (0.25)	5.61 (0.77)	7.43 (0.43)	10.05 (0.36)	12.14 (0.38)	15.07 (1.17)	17.24 (1.21)	31.57 (14.45)	34.17 (14.98)	38.27 (13.2)	97 94	3 6	2,974	
						[2.8–4.7]	[4.7–9.4]	[6.8–9.7]	[9.1–11.9]	[11.5–13.7]	[11.3–24.8]	[11.9–27.9]	[18–65]	[18–70]	[18–71]				
					M	3.31 (0.25)	5.71 (0.84)	7.44 (0.46)	10.07 (0.36)	12.16 (0.37)	15.02 (1.12)	17.1 (1.06)	34.8 (16.29)	37.5 (16.89)	41.91 (15.39)				
						[2.9–4.3]	[4.8–8.5]	[6.8–9.6]	[9.1–11.9]	[11.5–14.5]	[11.8–25.7]	[12.7–26]	[18–72]	[18–77]	[18–74]				
General exposome	3,859	66^c^	3,055	804	W	3.29 (0.24)	5.53 (0.68)	7.39 (0.4)	10.08 (0.37)	12.17 (0.38)	15.23 (1.34)	17.28 (1.17)	20.87 (7.15)	23.01 (6.51)	30.22 (7.15)	86 85	5 6	2,569	
						[2.8–4.5]	[4.7–9.4]	[6.8–9.7]	[9.4–12.7]	[11.5–13.7]	[10.4–25.5]	[13.2–25]	[18–59]	[18–60]	[24–66]				
					M	3.29 (0.23)	5.53 (0.68)	7.37 (0.39)	10.09 (0.36)	12.17 (0.4)	15.25 (1.41)	17.23 (1.18)	21.81 (9.19)	23.56 (7.97)	31.54 (9.37)				
						[2.9–4.3]	[4.7–8.5]	[6.8–9.7]	[9.4–11.9]	[11.5–14.8]	[13.2–25.4]	[13.6–25.6]	[18–62]	[18–64]	[24–70]				
Specific exposome + genome	897	73	701	186	W	3.25 (0.21)	5.29 (0.33)	7.26 (0.23)	9.95 (0.28)	12.15 (0.37)	14.91 (0.59)	17.09 (0.58)	18.54 (0.76)	21.32 (2.18)	27.97 (2.33)	97 94	3 6	625	
						[2.9–4]	[4.7–7]	[6.8–8.8]	[9.4–11.1]	[11.5–13.5]	[13.2–18.4]	[15.7–18.7]	[18–22]	[18–26]	[23–33]				
					M	3.26 (0.21)	5.27 (0.25)	7.25 (0.21)	9.97 (0.29)	12.17 (0.37)	14.9 (0.64)	17.02 (0.53)	18.42 (0.65)	20.91 (2.19)	27.55 (2.46)				
						[2.9–3.9]	[4.8–6.7]	[6.8–8.1]	[9.4–11.1]	[11.5–13.3]	[13.2–16.2]	[16.1–18.3]	[18–20]	[18–26]	[24–32]				
Specific + general exposome	1,080	71^b^	868	212	W	3.25 (0.21)	5.29 (0.31)	7.26 (0.24)	9.96 (0.31)	12.15 (0.36)	14.92 (0.6)	17.11 (0.58)	18.53 (0.75)	21.16 (2.13)	28.09 (2.15)	94 95	5 5	818	
						[2.9–4.2]	[4.7–7]	[6.8–8.8]	[9.4–12.3]	[11.5–13.5]	[13.2–18.4]	[16.1–18.7]	[18–21]	[18–26]	[24–33]				
					M	3.26 (0.21)	5.27 (0.25)	7.25 (0.21)	9.98 (0.31)	12.13 (0.34)	14.89 (0.64)	17.05 (0.55)	18.45 (0.7)	20.76 (2.01)	27.96 (2.06)				
						[2.9–3.9]	[4.8–6.7]	[6.8–8.1]	[9.4–11.1]	[11.5–13]	[13.2–16.2]	[16.1–18.8]	[18–20]	[18–26]	[24–33]				
Genome + general exposome	1,801	68	1,443	358	W	3.28 (0.24)	5.56 (0.73)	7.4 (0.41)	10.05 (0.35)	12.13 (0.36)	15.11 (1.14)	17.19 (0.97)	19.98 (5.18)	22.8 (5.09)	29.64 (5.52)	90 5	3 89	1,180	
						[2.8–4.5]	[4.7–9.4]	[6.8–9.6]	[9.4–11.9]	[11.5–13.5]	[11.3–24.8]	[13.2–23.4]	[18–54]	[18–56]	[24–61]				
					M	3.29 (0.24)	5.63 (0.8)	7.4 (0.44)	10.09 (0.36)	12.15 (0.35)	15.02 (0.85)	17.06 (0.8)	20.88 (7.39)	23.25 (6.88)	30.63 (7.93)				
						[2.9–4.3]	[4.8–8.5]	[6.8–9.6]	[9.4–11.6]	[11.5–13.7]	[13.2–21.3]	[13.9–23.4]	[18–62]	[18–64]	[24–70]				
Specific exposome + genome + general exposome	702	73	556	146	W	3.25 (0.21)	5.29 (0.34)	7.27 (0.23)	9.95 (0.28)	12.14 (0.36)	14.9 (0.6)	17.12 (0.58)	18.52 (0.75)	21.36 (2.18)	28.21 (2.17)	96 94	3 6	522	
						[2.9–4]	[4.7–7]	[6.8–8.8]	[9.4–10.9]	[11.5–13.5]	[13.2–18.4]	[16.1–18.7]	[18–21]	[18–26]	[24–33]				
					M	3.27 (0.21)	5.27 (0.27)	7.25 (0.22)	9.97 (0.3)	12.13 (0.35)	14.93 (0.68)	17.02 (0.54)	18.38 (0.65)	20.76 (2.18)	27.9 (2.24)				
						[2.9–3.9]	[4.8–6.7]	[6.8–8.1]	[9.4–11.1]	[11.5–13]	[13.2–16.2]	[16.1–18.3]	[18–20]	[18–26]	[24–32]				

Fam., families; W, women; M, men.

The migration background of the participants was determined following the official definition of the Dutch statistics bureau: if at least one parent is born outside of the Netherlands, the background of the participant is ‘non-Dutch’.

^a^In case columns do not add to 100, remaining percentages are ‘unknown’.

^b^One observation gender unknown.

^c^Two observations gender unknown.

## Results

### Unimodal analyses

**Fig. 1:**
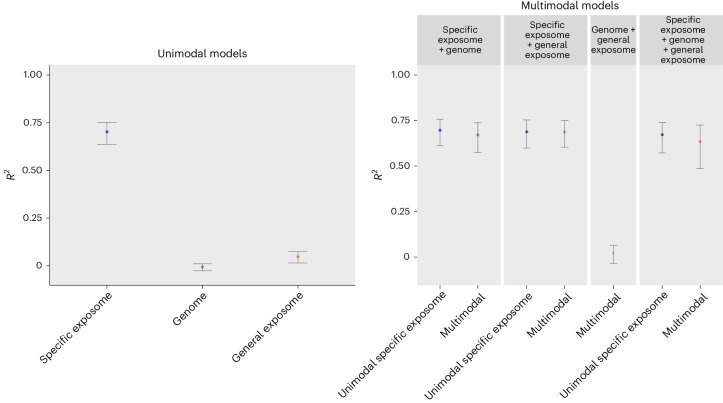
Model performance (*R*^2^) of unimodal and multimodal analyses. Error bars represent 95% CIs based on the distribution of 10,000 family-wise bootstrap samples (lower bar given by the 2.5th percentile, upper bar given by the 97.5th percentile), with the *R*^2^ value obtained in the independent test set shown in the center.

The performance of the model including specific exposome predictors in the independent test set was high given conventional standards (*R*^2^ = 0.702, 95% confidence interval (CI) [0.637–0.753]; Fig. 1). The genome showed not to be predictive of well-being (−0.007 [−0.026–0.010]), while our initial general exposome model showed small but significant predictive power as indicated by the confidence interval not including zero (0.036 [0.011–0.057]). However, many general exposome features were characterized by distributions with many zeros (for example, the number of swimming pools within a 1 km radius usually is zero). As a sensitivity analysis, we therefore first dichotomized all general exposome features for which the mode was zero and subsequently re-ran our unimodal general exposome model. Given the considerably higher *R*^2^-value of this model (0.047 [0.015–0.076]) and our aim of building optimal models, results based on transformed general exposome features are reported below (nontransformed results are in Supplementary Material 3).

### Multimodal analyses

As reported in Fig. 1, the multimodal model including genetic and specific exposome predictors showed an *R*^2^ value of 0.671 [0.574–0.738], while the model including general and specific exposome predictors reached an *R*^2^ value of 0.688 [0.606–0.750]. In line with the unimodal results for the general exposome and genome, the combination of the environmental and genetic features showed little predictive power for well-being (0.022 [−0.034 −0.066]). When all three data modalities were included, an *R*^2^ of 0.634 [0.490–0.728] was reached. We tested whether adding the genetic and general exposome beyond the specific exposome improved prediction by re-estimating unimodal specific exposome models in the smaller multimodal samples (Methods and Fig. 1) and comparing mean squared errors across models. Neither the addition of the genome (*Z* = 0.972, *P* = 0.334, *Δ*_MSE_ (difference in mean squared errors) = 0.118 [−0.120, −0.356]), nor the general exposome (*Z* = 0.392, *P* = 0.695, *Δ*_MSE_ = 0.045 [−0.180, 0.270]), nor both (*Z* = 2.104, *P* = 0.029, not significant compared with our 0.005 threshold, *Δ*_MSE_ = 0.289 [0.020–0.559]), significantly increased model performance. In fact, model performance decreased somewhat when adding the other two data modalities beyond the specific exposome. However, in the model including the specific and general exposome, two general exposome features were in the top 15 most predictive features: the number of newly built social rent houses around age 12 was the third most predictive feature (SHAP), and household income of housing of benefit receivers was the 14th most predictive feature (permutation importance). Similarly, in the model based on all three data modalities, the number of newly built social rent houses around age 12 was the fifth most predictive feature (SHAP).

### Feature importance

#### Unimodal specific exposome

**Fig. 2:**
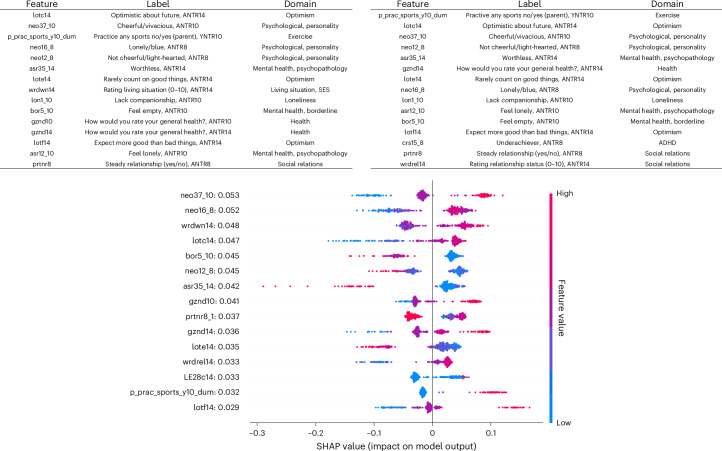
Feature importance of the specific exposome. Top: SHAP (top left) and permutation importance values (top right) of the top 15 specific exposome features across all three machine learning models (extreme gradient boosting, SVM and RF). Bottom: SHAP values of top 15 phenotypic specific exposome features based on optimal extreme gradient boosting model. LE28c14, life event: had a child (1–5 years ago). Values shown on *y* axis are mean absolute SHAP values per feature.

In the unimodal specific exposome model, the top 15 items from adulthood related to optimism, loneliness, personality (neuroticism and extraversion), subjective health, mental health traits (emptiness and worthlessness) and social relations and support (Fig. 2). One item was from an attention deficit hyperactivity disorder measure (‘underachiever’). For both SHAP values and permutation importances, one top 15 item was measured in childhood, that is, parental exercise behavior around age 10. The remaining top items were measured in adulthood.

The bottom panel of Fig. 2 shows the SHAP values of the top 15 features in relation to their feature values, based on the optimal XGB model. While most features show a relatively linear association with well-being, others show nonlinear associations. An illustrative example is bor5_10 (‘feel empty’; a borderline personality scale item). Although high scores on this item are always predictive of lower well-being levels, there is a subgroup of people with high items scores for which the influence is relatively small (SHAP values of approximately −0.07) and similar to those with intermediate scores on this item. For others, the influence is larger (SHAP values between −0.10 and −0.15). Similarly, for four individuals, agreeing with asr35_14 (‘worthless’) has a very strong negative influence on well-being, while for others this relation is less strong. Some symptoms may thus contribute to well-being in different ways across individuals.

#### Unimodal general exposome

**Fig. 3:**
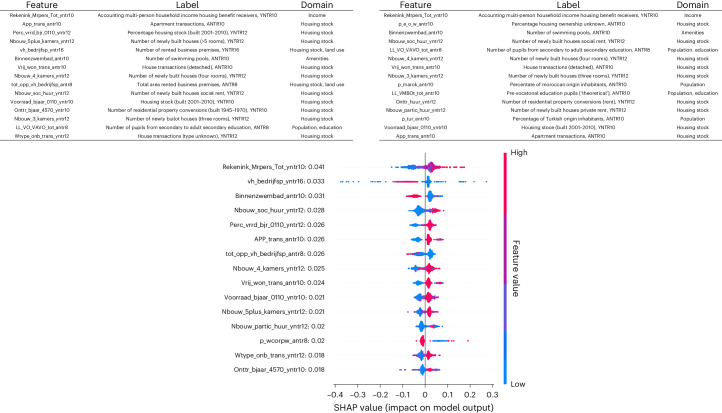
Feature importance of the general exposome. Top: SHAP (left) and permutation importance values (right) of top 15 general exposome features across all three machine learning models (extreme gradient boosting, SVM and RF). Bottom: SHAP values of top 15 phenotypic specific exposome features based on an optimal extreme gradient boosting model. p_wcorpw_antr8, percentage public housing in ANTR8. Values shown on *y* axis are mean absolute SHAP values per feature.

Figure 3 shows the top 15 (dichotomized when the mode was zero) general exposome features (top) and the top 15 SHAP values based on the optimal extreme gradient boosting model (bottom). Notably, most features are related to housing stock and the large share of top 15 features from childhood/adolescence (10 (67%) for SHAP values, and 7 (47%) for permutation importances).

The bottom panel of Fig. 3 clearly shows nonlinear patterns in the prediction of well-being for a number of general exposome features. For example, a low number of rented businesses premises in the area around age 16 (vh_bedrijfsp_yntr16) has a strong positive contribution to well-being for some individuals, while for others the contribution is strong and negative. As another example, a higher percentage of public housing in the area measured at wave ANTR8 (p_wcorpw_antr8) generally has a very small negative effect on well-being, but for some individuals the influence is strong and positive.

We refrain from reporting feature importance values for the unimodal genome model because of its nonsignificant predictive performance (see Supplementary Material 1).

### Longitudinal prediction based on the specific exposome

**Fig. 4:**
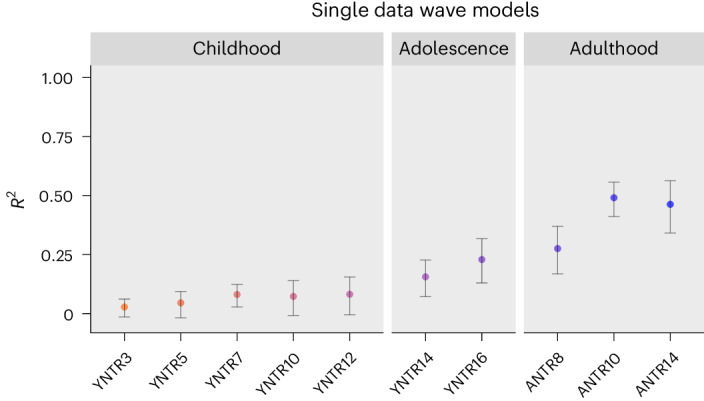
Model performance (*R*^2^) based on single waves of phenotypic (specific exposome) data. Error bars represent 95% CIs based on the distribution of 10,000 family-wise bootstrap samples (lower bar given by the 2.5th percentile, upper bar by the 97.5th percentile), with the *R*^2^ value obtained in the independent test set shown in the center.

Single waves of specific exposome data from early childhood showed nonsignificant *R*^2^ values at age 3 (0.028 [−0.015–0.060]) and age 5 (0.045 [−0.018–0.094]), as shown in Fig. 4. Model performance at age 7 (0.081 [0.028–0.124]), age 10 (0.073 [−0.009–0.141]) and age 12 (0.082 [−0.006–0.154]) were highly similar. In adolescence, model performance increases steadily with time of measurement being closer to adulthood (YNTR14: 0.156 [0.069–0.228] and YNTR16: 0.229 [0.133–0.315]). Performance of single waves in adulthood ranged from 0.275 [0.169–0.369] for ANTR8, to 0.491 [0.413–0.556] for ANTR10, with ANTR14 in between (0.463 [0.340–0.562]). Across the ten single wave models, model performance was positively associated with the total number of available features for each wave before (*r*(8) = 0.771, *P* = 0.009 [0.276–0.943]) and after feature selection (*r*(8) = 0.732, *P* = 0.016 [0.191–0.932]). Sensitivity analyses showed, however, that the increased prediction by more proximal features was not solely due to the sheer number of features (Supplementary Material 2).

**Fig. 5:**
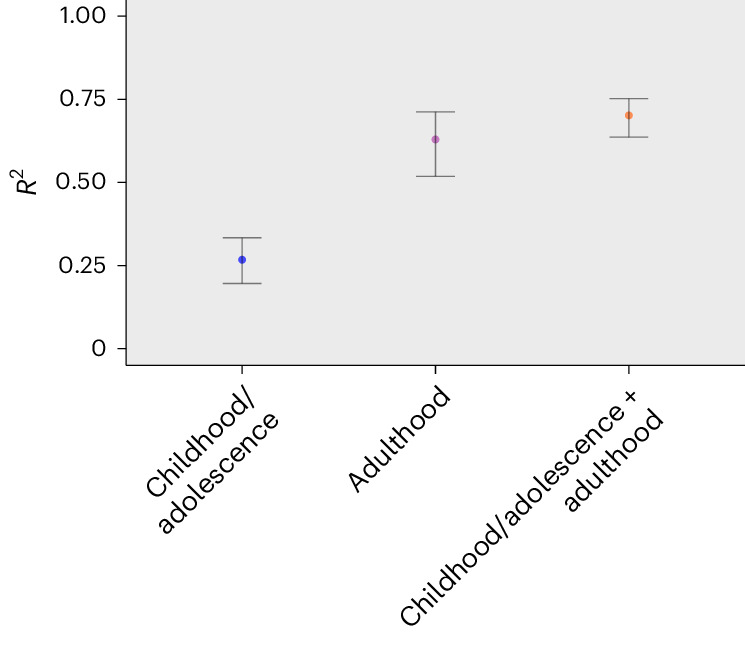
Model performance (*R*^2^) based on the type of longitudinal phenotypic (specific exposome) data. Error bars represent 95% CIs based on the distribution of 10,000 family-wise bootstrap samples (lower bar given by the 2.5th percentile, upper bar by the 97.5th percentile), with the *R*^2^ value obtained in the independent test set shown in the center.

*R*^2^ values of models based on all features from childhood/adolescence and adulthood separately, and their combination, are presented in Fig. 5. The model based on only childhood and adolescence features reached an *R*^2^ of 0.268 [0.193–0.333], while the performance of the model including only (all) adulthood waves (0.629 [0.517–0.712]) was significantly higher (*Z* = −6.219, *P* < 0.001, *Δ*_MSE_ = −0.745 [−0.980–−0.510]). The difference in performance between the model based on solely adulthood features and the full model based on all childhood/adolescence and adulthood features combined (0.702) was not significant (*Z* = 0.892, *P* = 0.372, *Δ*_MSE_ = −0.089 [−0.283–0.106]).

Note that model performance of all childhood and adolescence waves (0.268) was only marginally higher than the model based on YNTR16 alone (0.229; *Z* = −1.936, *P* = 0.051, *Δ*_MSE_ = −0.194 [−0.390–0.002]). In addition, in the model based on all childhood and adolescent wave features, 7 (SHAP) and 8 (permutation importance) out of 15 top features were from YNTR16, suggesting that this prediction was dominated by YNTR16 features. Thus, the predictive power of the ‘all childhood/adolescence waves features’ model was largely due to YNTR16 features.

## Discussion

Using ten data waves from a large population cohort collected between 1991 and 2022, machine learning models were trained on the basis of an extensive set of predictors from the genome (PGSs), the specific exposome (longitudinal psychosocial predictors) and general exposome (longitudinal objectively measured environmental exposures) to predict well-being in adulthood. In our unimodal specific exposome models, the large amount of predictors from childhood to adulthood resulted in prediction of well-being with high accuracy (*R*^2^ = 0.702). The most important features were optimism, loneliness, personality traits, other mental health traits and social relations and support. The general exposome showed a modest predictive value for well-being (*R*^2^ = 0.047) on its own, but adding its features to the specific exposome did not increase prediction. PGSs for traits from different domains were not significantly predictive of well-being, neither independently nor incrementally predictive beyond the specific exposome. Results were robust to several sensitivity analyses with respect to the number of outcome measurements, outliers, number of features and feature-to-sample ratio (Supplementary Material 2). Importantly, the predictiveness of the specific exposome compared with the genome and general exposome was not solely due to differences in feature numbers (2615, 60 and 732, respectively). Relatedly, feature-to-sample ratios may have been partly responsible for the fact that adding the genome and general exposome beyond the specific exposome decreased performance somewhat, but sensitivity analyses showed this not to be the sole reason for this finding (Supplementary Material 2).

Our specific exposome model performance was highly similar to a recent study reporting *R*^2^ values between 0.70 and 0.80 across models for the prediction of well-being over one year^41^. Yet, it is important to highlight how our study differed from Oparina et al. (2022), who obtained much lower *R*^2^ values (about half) while following a similar data-driven approach. First, we included a much larger number of potential predictors, including longitudinal predictors from childhood and adolescence, and from multiple adult waves. Longitudinal analyses showed that although proximal features were more predictive, the addition of distal features from childhood and adolescence increased prediction. In line with this, about half of the predictors (43%) selected by the feature selection algorithm were from childhood and adolescent waves. Second, our feature set consisted of high-quality features including personality and other mental health conditions (for example, anxiety and depression) while Oparina et al. (2022) largely included economic predictors. Yet, our models excluding mental health features (as in Oparina et al. 2022) also performed well (Supplementary Material 2), owing to the quantity and quality of the remaining features. A final important distinction is our use of a well-being factor score rather than a single item measure, reducing measurement error and improving model performance. Notably, when comparing results based on single waves of adult data, our findings were in line with Oparina et al. (2022), suggesting robustness of our results.

The environmental exposures showed modest predictive power on their own. In our optimal model—in which features with a mode of zero were dichotomized—half of the top features were measured in childhood/adolescence and predominantly related to housing stock. In the Netherlands, the composition of houses in a neighborhood (for example, public/social versus private) is an important indicator of neighborhood SES^44,45^, with public housing being more concentrated in cities^46^. Our results are in line with studies finding associations between (childhood) housing characteristics and important life outcomes, including well-being^47–49^. In our models including nondichotomized features, feature domains were more diverse (for example, income, population composition, education and amenities), although most indicators were (in)directly related to neighborhood SES and/or urbanization. Some of them were identified as risk/protective factors of mental health (for example, depression, anxiety and psychological distress) in previous studies^16,17^. Previously advocated risk/protective factors for well-being such as air pollution and greenspace^20,50^, although included in our feature set, did not appear to be predictive. Our results underline important differences in what it means to predict (versus associate); these exposures typically show small effect sizes when studied in isolation, and when many other exposures are included their effects may be further depreciated, limiting their collective predictive power.

Drawbacks related to objective environmental exposures linked to participants’ postal codes may have reduced their predictive power. For some exposures, the spatial resolution may not be fine-grained enough; for air pollution, the street address or even the floor level may be most informative^51^. Our approach also assumes uniform exposure within the same postal code, ignoring that people are exposed to environmental pressures when they commute, are at work, go to gym and meet with friends. Future research should explore ecological momentary assessment with passive global positioning system tracking on smartphones to capture the dynamic relationship between the environment and well-being^52^.

The selected PGSs were for traits previously (genetically) associated with well-being (for example, childhood maltreatment, resilience and loneliness), but also for more distal traits (for example, circadian rhythm and smoking cessation). Yet, collectively, the selected PGSs showed limited predictive power in the independent test set, which may be due to several factors. First, PGSs are based on genome-wide association studies (GWAS) that only capture the tiny effects of a restricted set of common variants; by increasing the variant coverage of PGSs and GWAS sample sizes, out-of-sample prediction may increase^53–55^. Relatedly, our genetic sample (*N* = 5,874) was perhaps still too limited for the PGSs to become predictive out-of-sample. With respect to the number of PGSs, we included a large number (60) covering multiple domains, but more extensive sets may be needed. As genotyping costs decrease, hopefully it will soon become possible to create PGSs for even larger numbers of traits and individuals, rendering the combined predictive power of PGSs for well-being high enough to be practically useful.

The findings of the current study can be used to formulate hypotheses on nonlinear pathways to well-being. Our SHAP values showed that the relation between some items and environmental exposures on the one hand and well-being on the other differed across groups of participants. If replicated, a next logical step would be to identify possible moderators of the effects of these items/symptoms. This can for example be achieved by associating individuals’ SHAP values with external variables such as gender and age, or other traits such as depression or educational level. For the environmental exposures, it would entail in-depth analyses as to why some aspects of the built environment (for example, low number of rented business premises in adolescence) affect well-being positively for some and negatively for others. The geographical context will have a large influence on this^48^: naturally, both neighborhoods with low and high SES, or highly urbanized or rural areas, can have a low numbers of rented businesses. In the end, risk stratifications based on the exposome may pave the way for personalized prevention and treatment options.

Our results have important implications both for future investigations of well-being and the practical utility of well-being prediction models. In line with well-being’s complexity, predictive features included early childhood circumstances and behavior, SES, substance use, personality, life events, psychopathology, and lifestyle behaviors (Supplementary Table 1). Future studies should embrace this complexity, avoiding a narrow focus on individual risk/protective factors. Several highly predictive features align with prior research (for example, social support, personality and self-rated health)^4,41^, suggesting potential targets for interventions. The current study also shows how a data-driven approach can generate new hypotheses on the importance of features. For example, parental exercise behavior around age 10 was highly predictive of well-being later in life. Future research should investigate through which pathways this may occur, for example, through social learning^56^ or health communication^57^. Further, the importance of consistent long-term monitoring is signified by multiple wave models outperforming single data wave models. Importantly, single waves of data were predictive of adult well-being from as young as age 7 onward. However, we did find proximal features to be more predictive than more distal features. This may be partly due to changes in rater effects, since childhood predictors were based on parent ratings and adolescence/adult ratings on self-reports. Additionally, the lack of incremental prediction of childhood predictors beyond adult predictors may be due to downstream effects of childhood differences. By controlling for those effects by including proximal adulthood features, inclusion of the childhood features would not contribute significantly to the model anymore. Still, the feature selection algorithm did choose (early) childhood features, and inclusion of more features from multiple waves childhood/adolescence boosted prediction considerably, underlining the importance of longitudinal information.

From a policy perspective, our general exposome results highlight the importance of initiatives targeted at housing. Although previous studies have shown associations between housing characteristics and well-being^49^, the current study shows that neighborhood housing stock in adulthood but also in childhood/adolescence can predict adult well-being at the individual level. These results are especially relevant given the current housing crisis in the Netherlands with long waiting lists for public housing for lower income residents, and surging housing prices in the private sector^49,58^. Our results suggest the importance of (local) governments prioritizing affordable housing, as it may have direct or downstream consequences on individuals’ well-being.

Some limitations of this study are worth mentioning. First, given our data-driven approach, we decided to only remove direct indicators of well-being from our feature set, and no other features based on conceptual or statistical considerations. This decision led to the inclusion of features that conceptually overlap with well-being (for example, optimism and self-esteem), perhaps somewhat artificially boosting the performance of our specific exposome models. Yet, a model excluding all mental health features altogether reached similar *R*^2^ values (Supplementary Material 2). Second, in each wave, only two or three hedonic well-being measures were available to identify well-being at that time point. Future studies should include more indicators, preferably including eudaimonic measures, to improve identifiability of the used factor model. Moreover, our results should be interpreted in relation to hedonic (subjective) well-being; although strongly (genetically) associated with eudaimonic and social well-being^1,59,60^, it is not certain that our results generalize to these well-being concepts. Third, our results are restricted to a Western Educated Industrialized Rich Democratic sample, limiting generalizability to other countries or contexts. Our general exposome results should also be interpreted in relation to the specific Dutch context (for example, high population density and high well-being), which may have influenced general exposome–well-being relationships^17^. For example, air pollution levels are comparatively low, and because of the Netherlands’ small size, regional differences are small^61,62^. In addition, many different, possibly opposing or indirect effects at different geographical levels may be operating simultaneously; neighborhoods’ housing prices are typically associated with improved mental health, but such neighborhoods are often found in Dutch urban areas associated with reduced mental health^16,17^. Similarly, in the Dutch context, blue space has been associated with reduced mental health due to the larger cities having many canals^17^. Together, this may have resulted in low predictive power of the general exposome at the individual level. Our study should thus be replicated in other countries, which differ in terms of, for example, population decomposition, neighborhood characteristics and urbanization grades. Fourth, women were overrepresented and people with a migration background underrepresented in our sample compared with the Dutch population. Fifth, due to attrition, our predictive models were applied to somewhat less happy and higher educated individuals compared with those who dropped out of the study, although differences were small. This may have affected our results if pathways to well-being differ across these groups. Further, our results may not generalize to clinical samples. External validation of our algorithms in external, longitudinal and more heterogeneous cohorts in terms of outcomes, predictors and demographics (gender and migration background) is thus needed. Finally, although we used longitudinal data, our results are still based on associations; causal interpretations of our findings are therefore unwarranted. Given that we included indicators associated with well-being in childhood and adolescence, bidirectional patterns and reverse causality may be responsible for adult well-being. For optimal prediction, the aim of this study, this is less of an issue given that the task is to predict individuals’ well-being, regardless of its origins. For understanding of how individuals develop high or low well-being levels, however, causality is more important; although our SHAP values analyses provide some insights, novel causal machine learning^63^ approaches are needed in the future.

To conclude, based on extensive, longitudinal assessments of the exposome, we are able to predict adult well-being levels with high and modest accuracy based on the specific and general exposome, respectively. Currently, there is no incremental prediction of general exposome and genome beyond the specific exposome, but the future may hold more promising results as more sophisticated methods for their assessment are rapidly developing. Combining them with the internal exposome (for example, microbiome and metabolome) may pave the way to personalized interventions based on one’s genetic background and exposome.

## Methods

### Sample

Data from ten study waves of the NTR were used, collected between 1991 and 2022. In the NTR, every 2/3 years, longitudinal surveys about lifestyle, personality, psychopathology and well-being in twins and their families are collected^64^. The NTR is a population-wide, nonclinical sample and distinguishes between the YNTR and the ANTR. The YNTR comprises children rated by their parents, and standardized surveys are sent out around the ages of 1, 2, 3, 5, 7, 9 and 12. For several years (2004–2014), YNTR twins aged 14, 16 and 18 were invited for self-report surveys. When participants in the YNTR reach the age of 18, they are invited to take part in the ANTR in which adult participants provide self-reports.

Data from the YNTR waves 3, 5, 7, 10, 12, 14 and 16 were used because these included the most consistently administered variables. We use a fully data-driven approach: features are included on the basis of availability, not on substantive considerations. Only mother reports were included, because mothers were most compliant in responding, thus maximizing sample size. Additionally, aggregated NTR variables were included as these include important variables for the prediction of well-being (for example, parental SES). For our outcome (well-being), we used ANTR waves 8, 10 and 14 based on the availability of relevant well-being variables. Signed informed consent, also for record linkage, was obtained from all individual participants included in the study. Participants did receive compensation for participation. For more information on data collection within the NTR, see refs. ^64–67^. Data was obtained by completing the data sharing request forms and approval of the NTR Data Access Committee.

In total three unimodal (specific exposome, genome and general exposome) and four multimodal (specific exposome/genome, specific/general exposome, genome/general exposome and all three) datasets were created. Demographic information on gender, age and migration background for all datasets are reported in Table 1. In childhood, gender was assessed by the parents, and in adolescence and in adulthood through a self-report question. Since biological information was not used to determine sex, the term ‘gender’ is relevant for this study. The migration background of the participant was determined following the official definition of the Dutch statistics bureau: if at least one parent is born outside of the Netherlands, the background of the participant is ‘non-Dutch’. In the majority of the cases (93% for mothers and 96% for fathers), parents’ birth countries were assessed through self-reports, otherwise information was based on Dutch census statistics. Because we did not use a self-identification question on ethnicity, we do not report the specific ethnic backgrounds of the non-Dutch participants. In the unimodal specific exposome sample, the mean number of study waves present was 8.96 (standard deviation (s.d.) 0.73), ranging between 6 and 10. Most people had data on nine study waves (53% of the sample). Across all waves, the average years between consecutive waves was 2.78 (s.d. 1.37), with a minimum average years of 1.60 (s.d. 0.96, range 0–5 years) between YNTR16 and ANTR8, and maximum average years of 6.17 (s.d. 0.43, range 5–8 years) between ANTR10 and ANTR14. A small number of participants completed two surveys within the same year; the largest number (51 or 3.6%) was found for the YNTR16 and ANTR8. In the unimodal general exposome sample, neighborhoods were spread all over the Netherlands (Supplementary Fig. 2). Across waves, the number of different six-digit postal codes ranged from 287 (YNTR3) to 2,511 (ANTR8). The mean number of participants per neighborhood (four-digit postal codes) across waves was 1.39 (s.d. 0.54), ranging between 1 and 15. Multimodal datasets were created by merging the preprocessed unimodal datasets.

Attrition analyses showed that the participants included (versus not included) in the analyses reported somewhat lower levels of well-being and were more highly educated, although effect sizes were small (Supplementary Table 2). Gender differences in attrition were negligible.

### Measures

#### Outcome: well-being

A continuous well-being score was created on the basis of the Satisfaction with Life Scale^68^, Subjective Happiness Scale^69^ and a measure of quality of life, the Cantril Ladder^70^. The Satisfaction with Life Scale consists of five items answered on a seven-point scale (1 = ‘strongly disagree’ to 7 = ‘strongly agree’). An example item is: ‘I am satisfied with my life’. The Subjective Happiness Scale consists of four items rated on a seven-point scale (1 = ‘strongly disagree’ to 7 = ‘strongly agree’). An example item is ‘On the whole I am a happy person’. The Cantril Ladder requires participants to indicate the step on a ladder at which they place their lives in general on a ten-point scale (10, best possible life, and 1, the worst possible life).

We fitted a latent trait–state–occasion model^71^ using structural equation modeling (in lavaan^72^) to compute an overall adult well-being factor score based on all available well-being measures from ANTR8, ANTR10 and ANTR14, for each respondent. For factor score estimation, the regression method was used with a transformation so that the covariance matrix of the estimated factor scores matched the model-based latent factor covariance matrix^73,74^. The outcome represents individuals’ stable, overall well-being levels while controlling for time-specific states and age at time of measurement (by regressing out age at each wave within the model; Supplementary Fig. 3). In this model, all data are preserved: even those with missing values on any of the outcomes will receive a general well-being score. Our outcome variable was restricted to adults (≥18 years). Participants were included if they had two or more well-being measure available (either within or across waves). To prevent ‘data leakage’ known to affect model performance^75^, the latent trait–state–occasion model was fitted in the training set and the model parameters based on this set were used to create factor scores in both the training and test set. Model fit (degrees of freedom, 4; *χ*^2^, 488.836; *P* < 0.001), Comparative Fit Index 0.985, Tucker-Lewis Index 0.976, Root Mean Squared Error of Approximation 0.028, Standardized Root Mean Squared Residual 0.029) was good according to common standards^76^. Correlations between the overall well-being score and its constituent traits were high, between 0.694 (quality of life, ANTR14) and 0.796 (satisfaction with life, ANTR14); Supplementary Fig. 4.

#### Features

For full lists of initially considered features, included features after preprocessing and feature selection, see Supplementary Table 1.

##### Phenotypic data (specific exposome)

We included individual items/symptoms of scales rather than sum scores in our models (for example, individual items of the NEO Five Factor Inventory for extraversion rather than the extraversion scale score). Some summary measures (within measurement wave) based on count variables were calculated for this study’s purposes (for example, calculating ‘total conditions present’ from individual medical conditions).

##### Genetic data (genome)

Genetic predictors were included through PGSs. PGSs are aggregate scores of an individual’s genetic predisposition for a given trait, calculated by summing risk alleles weighted by their effect sizes from discovery GWAS^77,78^. Given our data-driven approach, PGSs for all traits readily available in the NTR were included. Supplementary Table 3 presents a list of the 60 PGSs included in this study and their original GWASs. The PGSs covered traits from a wide range of domains (for example, personality, childhood health, childhood psychopathology, substance use, SES and exercise behavior).

PGSs were calculated in the standardized pipeline within the NTR. In short, variants for which the effect allele frequency was 0.01≤ effect allele frequency ≤0.99 are retained. The allele frequencies and effect sizes of the variants are aligned with the NTR reference for the 1,000 genomes variants. Variants not included in this reference were excluded. The processed summary statistics serve as input for the LDpred 0.9 software. For estimating the target linkage disequilibrium structure a (1) selection of unrelated individuals in the NTR sample are used and (2) a set of well-imputed variants in the NTR sample are selected. The parameter ld_radius is set by dividing the number of variants in common (from the coordination step output) by 12,000. For the coordination step, the median sample size is used as the input value for *N*. The plink2 software package generates the PGSs (using the –score option) to the input weighted effect sizes and the genotype dataset. We include PGSs based on the infinitesimal model, that is, including all SNPs. In all models including PGSs, as covariates the first ten genomic principal components to control for population structure and genotyping platform dummies were included.

##### Environmental exposures (general exposome)

For the general exposome, we leveraged the Geoscience and health Cohort Consortium database^79^, which has previously been used in combination with the NTR^7^ and in other studies on mental health^17^. This database includes a wide range of objective environmental exposures at the neighborhood (postal code) level (for example, liveability, safety, population composition, air pollution and neighborhood SES indicators) based on several data sources^80^. A full list of available exposures can be found elsewhere (https://www.gecco.nl/exposure-data-1/), with more information on the included features is reported in Supplementary Tables 1 and 4. All available exposures for the time period 1990–2022 were requested through the data access form. Geoscience and health Cohort Consortium database includes measurements of exposures per year, and for each wave of data collection within the NTR, the postal code of the respondent was available. When participants register for the NTR, they are asked to provide their address. Participants are asked to contact the NTR when they move so that their address can be updated. In addition, addresses are regularly updated by cross-checking with the Dutch Personal Records Database (in Dutch: Basis Registratie Personen). For some participants, postal code information may not be accurate if they move without notification. We checked this in a subset of participants for which self-report postal codes (four digits) were available in the child and adolescent waves: in ~90% of the cases (range 88–94%), the postal codes in the database matched with self-reported values. For each individual, the postal code at the year of survey completion was matched with the exposure of that year. Exposures were linked to the NTR data either using participants’ six- or four-digit postal code depending on which level a given feature was available. For features with different spatial resolutions available (for example, number of supermarkets within a 500 m, 1,000 m or 1,500 m radius), we chose a resolution of 1,000 m (for example, ref. ^18^).

### Analyses

The reported analyses are part of a larger preregistration (https://osf.io/msbvp), for additions and deviations see https://osf.io/tuhgs. Before any analyses were done, the dataset was divided into two independent datasets in an 80/20% split, the former (*N*_train_) being used for feature selection, training and validation using tenfolds cross-validation, while the second outheld dataset (*N*_test_) was used for final model testing (Supplementary Fig. 1). The NTR includes family data with relatives being nonindependent observations. Therefore, members of the same family were not split across training and test set in order to prevent overfitting that could occur when they are separated between datasets. In addition, grouped tenfolds cross-validation was used in training to take relations between family members into account.

We ran all machine learning models for each unimodal dataset, all possible pairwise combinations, and a model including all three data modalities, resulting in a total of seven sets of analyses. For each set, we maximized the sample size, leading to different sample sizes across analyses (Table 1). However, in the multimodal analyses, we compared the multimodal model performance (for example, specific exposome + genome) with the performance of a unimodal specific exposome model based within this same (reduced) sample for optimal comparability.

The feature selection step was only applied in the unimodal analyses: the multimodal analyses were based on the features that were selected in each of the three unimodal analyses. This made the unimodal and multimodal models readily comparable: if we would have entered all three data modalities into the feature selection algorithm, then only the phenotypic features would have been selected since the predictive power of PGSs and objective environmental exposures is much lower compared with the phenotypic features. In addition, the unimodal analyses were based on larger sample sizes than the multimodal analyses ensuring reliability and generalizability of the features.

The availability of longitudinal specific exposome data allowed predicting adult well-being from previous time points by training and testing models based on different slices of features. In the unimodal specific exposome dataset, we first trained models based on features from each wave separately (Fig. 4), on features only from childhood/adolescence (that is, YNTR3 until YNTR16), and on features only from adulthood (all three adult waves jointly), and compared them with the full phenotypic model including all childhood/adolescence and adulthood features described earlier (Fig. 5).

#### Preprocessing

All preprocessing steps were informed on the training data and based on this the test data were preprocessed to avoid data leakage.

##### Removing and transforming features

Features were screened for nonsensible values, and if present, set to missing. Features with zero variance and character (that is, text) features were removed. All twin-specific features were removed since we aimed at building prediction models for the general population. All direct well-being (that is, outcome) indicators and features including the words ‘happy’ or ‘happiness’ were excluded from the adult surveys, as this would overlap too much with the outcome. Models including direct well-being indicators in childhood and adolescence are reported in Supplementary Material 3. All continuous variables were first standardized and subsequently normalized (range 0–1), while categorical variables were dummy encoded.

As reported elsewhere^7,17^, the general exposome features showed strong correlations with each other and many were highly skewed. In our main analyses, in our general exposome dataset, we iteratively removed variables with correlations >0.95 with other exposures until no more of these features remained. Using the e1071 (ref. ^81^) package, all general exposome features were transformed by cubing them (skewness <1) or taking their cube root (skewness values >1).

##### Missing value imputation

The postal codes of the participants showed a relatively large number of missing values. To increase the general exposome sample size, we imputed missing values as follows. For many individuals, gaps in available postal codes between waves were found, even though no changes occurred in recorded postal codes. As an example, postal code 1111AA would be recorded for an individual at YNTR5, then two missing values for waves YNTR7 and YNTR10, and then a record of postal code 1111AA at wave YNTR12. In that case, we assumed the individual did not move in between waves and missing values were imputed with the recorded values (that is, 1111AA). For the year of exposures to link the imputed postal codes to, linear interpolation was used: in the previous example, if YNTR5 was completed in 2002, and YNTR12 in 2008, then exposures were selected from 2004 (YNTR7) and 2006 (YNTR10). Given the high stability of objective environmental exposures over time^17^, this linear interpolation scheme can be expected to give reliable results.

In the unimodal datasets, participants and features with more than 55% missing values were excluded (deviating from our preregistered 50% as this would have reduced our sample sizes too much). Remaining missing values were imputed using the *k*-nearest neighbors^82^ method, a relatively simple nonparametric multivariate imputation technique more time efficient than more complex imputation techniques^83^. Following common practice, we chose *k* by taking the square root of the number of observations in each of the respective datasets.

##### Feature selection

Because of the relatively large number of features compared with the number of individuals, feature selection was applied to reduce the number of features before prediction. We applied elastic net regression^84^ (with tenfolds cross-validated tuning on the training set), a combination of ridge and Least Absolute Shrinkage and Selection Operator (LASSO) regression, previously shown to provide more stable results than either methods separately^85^.

Out of 2,615 specific exposome features, 212 features from several domains (Supplementary Table 1) were selected. These included 91 (43%) features from childhood and adolescence (each wave contributing at least five features), the remaining 121 (57%) being measured in adulthood (Supplementary Table 5). For the genome, 13 out of 60 features were selected (PGSs for agreeableness, asthma, childhood bodymass index, childhood maltreatment, circadian rhythm, educational attainment, household income, loneliness, moderate to vigorous physical activity, pubertal growth, resilience, smoking cessation and well-being). Finally, for the general exposome, 29 out of 732 exposures were selected in our dichotomized feature models (Results), including 15 features (52%) from adolescence (3 from YNTR10, 9 from YNTR12, 2 from YNTR14 and 1 from YNTR16). These features mostly related to housing stock (for example, number of newly built houses, house transactions, number and area of rented business premises, and percentage public housing), but also to population decomposition (for example, number of education pupils, percentage of inhabitants with non-western migration backgrounds), amenities (for example, kernel density local food shops in 1,000 m radius), and income (multi-person household income of housing benefit receivers). In our nondichotomized general exposome model, 36 features were selected with 18 of them (50%) from adolescence (4 from YNTR10, 8 from YNTR12, 2 from YNTR14 and 4 from YNTR16). These exposures related to housing stock (for example, apartment transactions), population decomposition (for example, percentage of inhabitants with non-western migration background and absolute number of single-person households), neighborhood statistics (for example, mortality and divorce rates), urbanization grade, amenities and neighborhood SES indicators (number of social security beneficiaries and percentage of inhabitants with a high (top 20%) income).

#### Model training

In line with previous studies^34,37^, we created a stacked ensemble model based on commonly used XGB, RF and SVM models. Stacked ensemble models train a second-level meta-learner based on the first-level (that is, XGB, RF and SVM) model predictions to arrive at an overall prediction. As each algorithm has its own advantages and limitations, the overall meta-learner tends to outperform each individual model by combining the inputs into a best guess^34,40,86^. For the final, second-level model we used the XGB model as it tends to perform well when predicting mental health^4,29^. Standard hyperparameters were tuned in a random grid search with 100 searches for each model, with grid ranges largely determined by previous studies^34,36,37,87^. Searched parameter grids and optimal (that is, tuned) combinations are reported in Supplementary Table 6. A standard ordinary least squares (OLS) regression based on the feature selected set was conducted as a baseline comparison.

#### Model evaluation

##### Performance

To evaluate model performance, the *R*^2^ of the optimal model (Supplementary Table 5) in the independent test set is reported. This metric was chosen for comparability with previous work^4^. Note that negative *R*^2^ values can be obtained if the model performs worse than simply predicting the outcome mean—typically a sign of overfitting in the training data. To compare performance across models, 95% CIs were estimated using nonparametric bootstrapping at the family level^88^ (10,000 samples). *R*^2^ values are deemed significant if the confidence intervals do not include zero. Nonparametric clustered Wilcoxon signed rank tests^89,90^ on the squared model prediction errors (that is, (*y*_pred_*i*_ − *y*_test_*i*_)^2^) were used to test whether the performance of two models (unimodal versus multimodal) differed significantly from each other. Throughout all analyses, all *P* values reported are based on two-tailed tests, using a conservative *P* value of 0.005 (ref. ^91^).

##### Feature importance

To derive meaning from our trained models, feature importances for each of the lower-level models were investigated using SHAP^43^ values. SHAP allows for the estimation of each feature’s importance for each individual prediction (that is, participant), by comparing model performance with and without each feature. The overall importance of each feature can then be calculated by taking the mean of the absolute SHAP values across all individual predictions. Features with the highest SHAP values (indicating higher importance) are identified and the top 15 are reported. Since we trained three different models (XGB, RF and SVM), we report the top 15 based on the mean feature importance value across these three models. Because different feature importance methods have their own strengths, we also report permutation importances, which measure the degree to which each algorithm relied on a given variable in making its predictions^4^.

All preprocessing was done in the R environment^92^. All machine learning analyses were conducted in Python using PyCharm in combination with Anaconda3 as the user interface; the RF and SVM models were conducted using the scikit-learn package^93^ and the extreme gradient boosting models were conducted using xgboost^94^. The TRIPOD Checklist for Prediction Model Development is reported in Supplementary Table 7.

### Ethics statement

All procedures conducted in studies involving human participants adhered to the ethical standards of the institutional and/or national research committee, as well as the 1964 Helsinki Declaration. Data collection was approved by the Central Ethics Committee on Research Involving Human Subjects of the University Medical Centers Amsterdam. Informed consent was obtained from all individual participants included in the study. Only participants who consented to record linkage were included. Ethical approval numbers are as follows: YNTR3/YNTR5/YNTR7/YNTR10/YNTR12 (94/105, 21-06-1994; 96/205, 14-01-1997; 99/068, 11-08-1999; 2003/182, 18-12-2003; and 2010/359, 18-02-2011), YNTR14/YNTR16 (2003/182, 18-12-2003; 16-12-2010; 12-09-2012; and 30-10-2013), ANTR8 (NL25220.029.08/2008-244), ANTR10 (2011/334; 12-10-2011, 2012/433; and 26-02-2013), ANTR14 (2018/389; 25-07-2018, VCWE-2018-124; and 16-08-2018) and genetic data (04.001.98; 25-05-2007).

### Reporting summary

Further information on research design is available in the Nature Portfolio Reporting Summary linked to this article.

## Supplementary information

Supplementary Information Supplementary Figs. 1–6 and Materials 1–3.

Reporting Summary

Supplementary Tables Supplementary Tables 1–10.

## Data Availability

Being part of a national prospective cohort study, the Netherlands Twin Register data cannot be made publicly available for privacy reasons, but they are available for legitimate researchers via the data access procedure at https://tweelingenregister.vu.nl/information_for_researchers/working-with-ntr-data. Data of the Geoscience and health cohort consortium (GECCO) can be requested via the data access request form at https://www.gecco.nl/exposure-data-1/.

## Code availability

Python scripts for the machine learning models can be found at https://osf.io/zphw8/.
