# Supplementary material for: Building machine learning prediction models for well-being using predictors from the exposome and genome in a population cohort
Source: Nat Ment Health. 2024 Aug 14;2(10):1217–30. doi: 10.1038/s44220-024-00294-2 (PMC11511667; doi:10.1038/s44220-024-00294-2)
Supplement: Supplementary file 1 — Supplementary Figs. 1–6 and Materials 1–3. [file 44220_2024_294_MOESM1_ESM.pdf]

nature mental health

Supplementary information

https://doi.org/10.1038/s44220-024-00294-2

Building machine learning prediction models for well-being using predictors from the exposome and genome in a population cohort

In the format provided by the authors and unedited

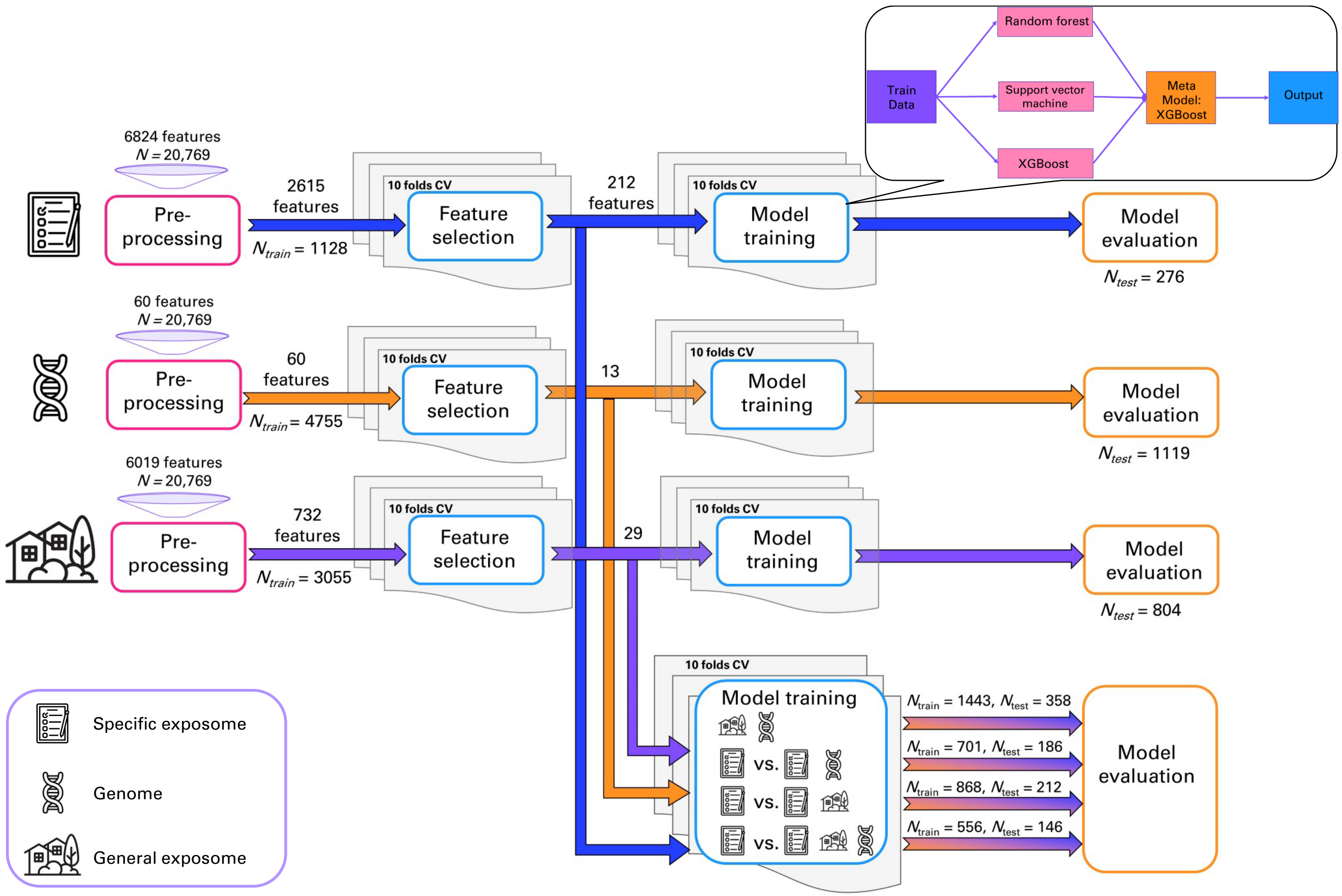

Figure S1. Machine learning pipeline. Pre-processing (pink boxes) was applied to both the training and (independent) test sets, feature selection and model training in the training sets (blue boxes), model evaluation (yellow boxes) in the test sets.

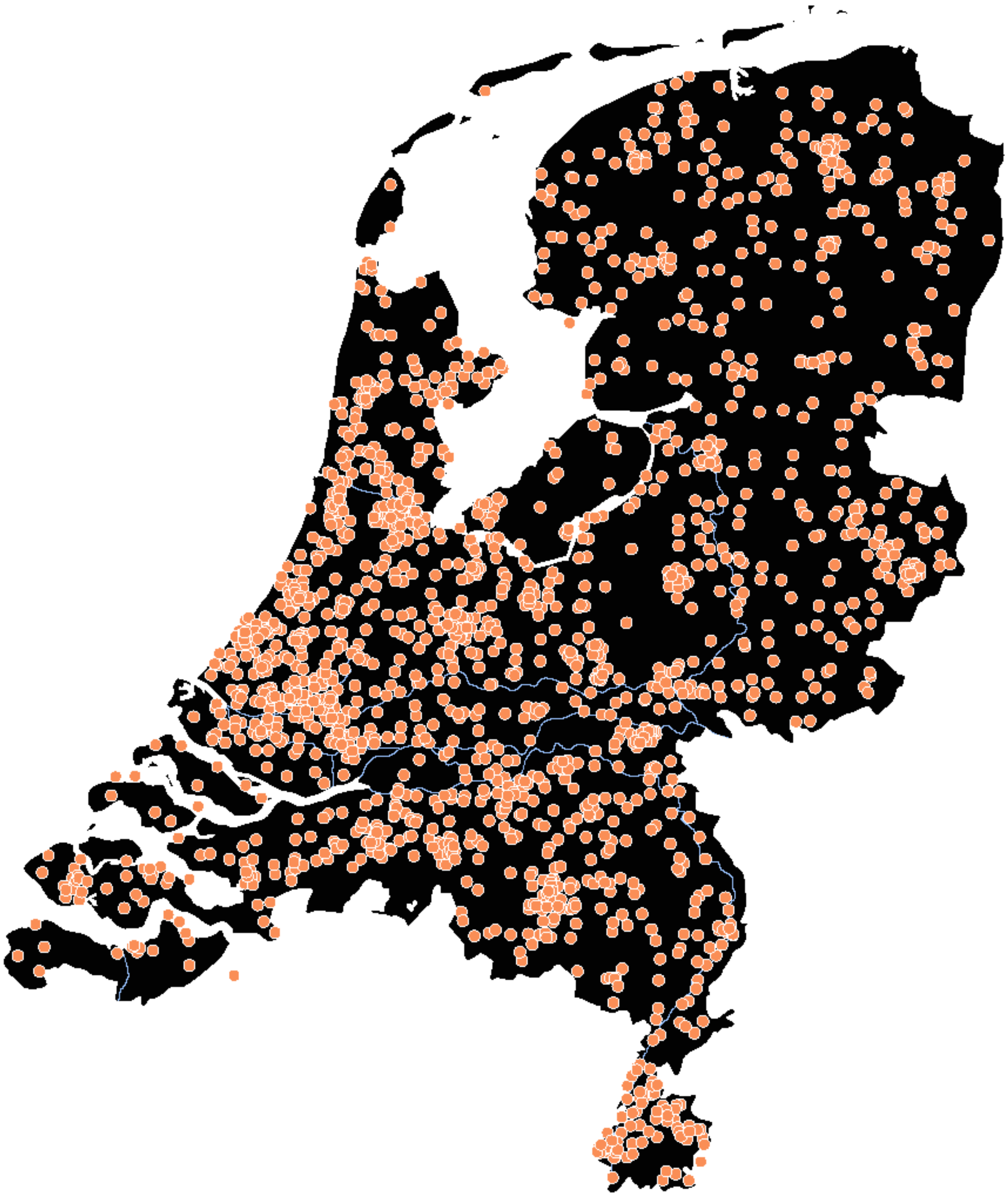

Figure S2. Distribution of participants' neighborhoods across The Netherlands. Each dot represents a postal code recorded in adulthood.

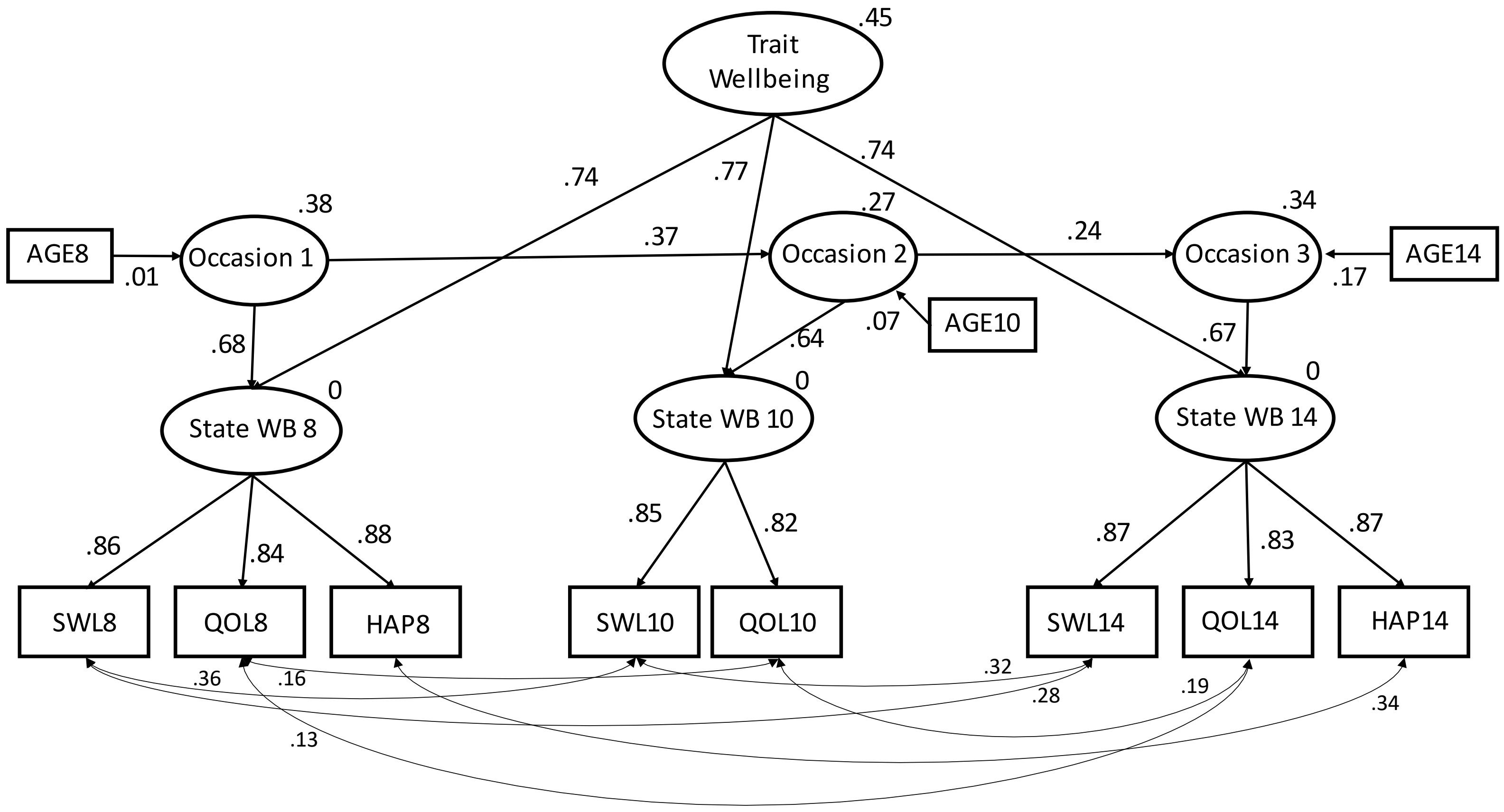

Figure S3. Latent trait-state-occasion model fitted to create outcome score (Trait Wellbeing). WB = Wellbeing; SWL = Satisfaction with life; QOL = Quality of Life; HAP = Happiness. Numbers refer to study waves. Loadings, regression paths, and correlations are standardized values, variances are unstandardized values. Fit measures: *df* = 34, *Chi-square* = 488.836 ($p < .001$), *CFI* = .985, *TLI* = .976, *RMSEA* =.028, *SRMR* = .029.

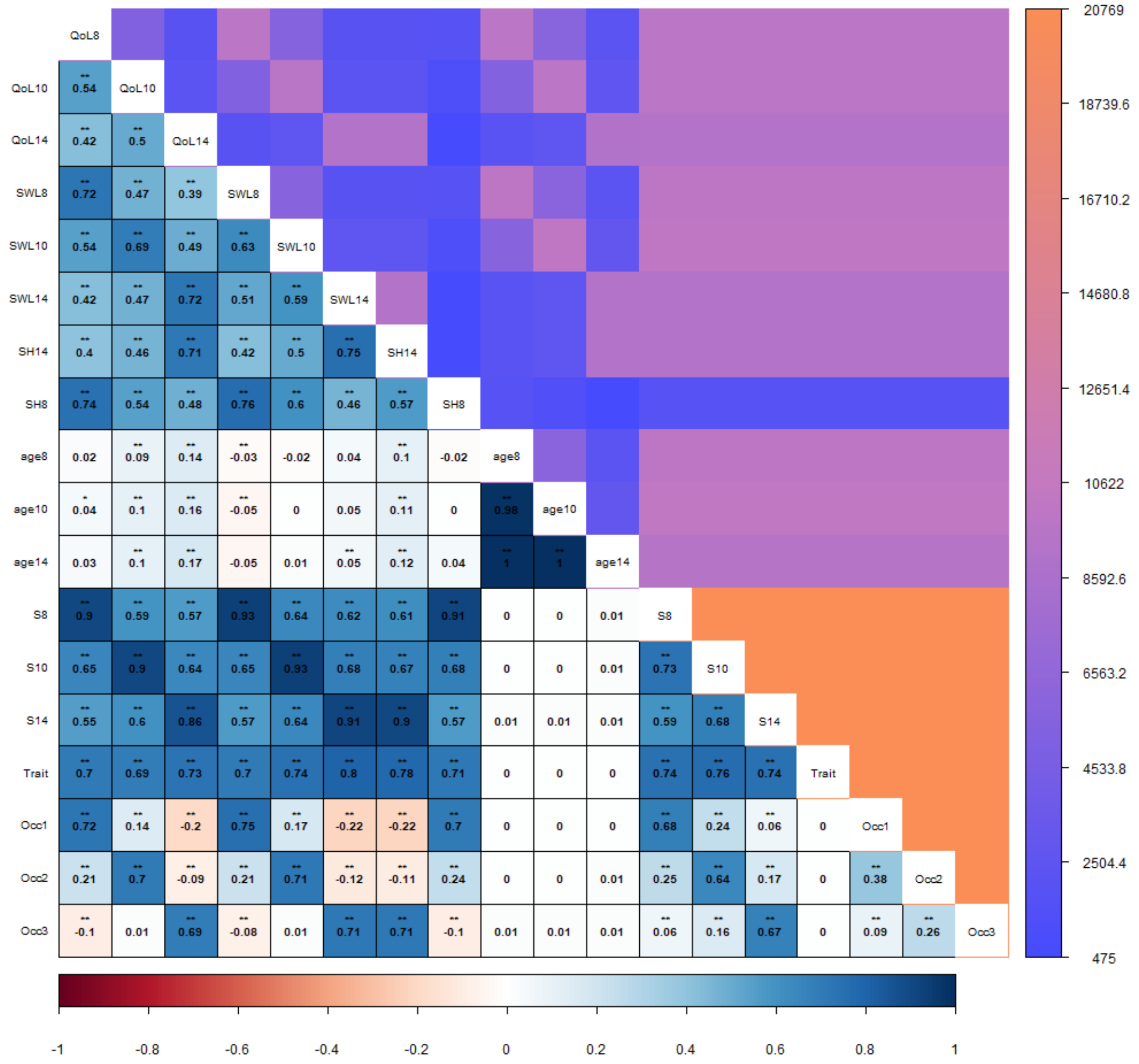

Figure S4. Correlations (i.e., Generalized Estimation Equation betas to correct for familial clustering) between outcome measures. Sample sizes presented in upper diagonal. Trait = outcome measure, S = state, QoL = Quality of life (Cantril Ladder), SWL = Satisfaction with life, SH = Subjective happiness, Occ = Occasion. Numbers 8, 10, and 14 refer to study waves. $^{*}p < .01$, $^{**}p < .001$

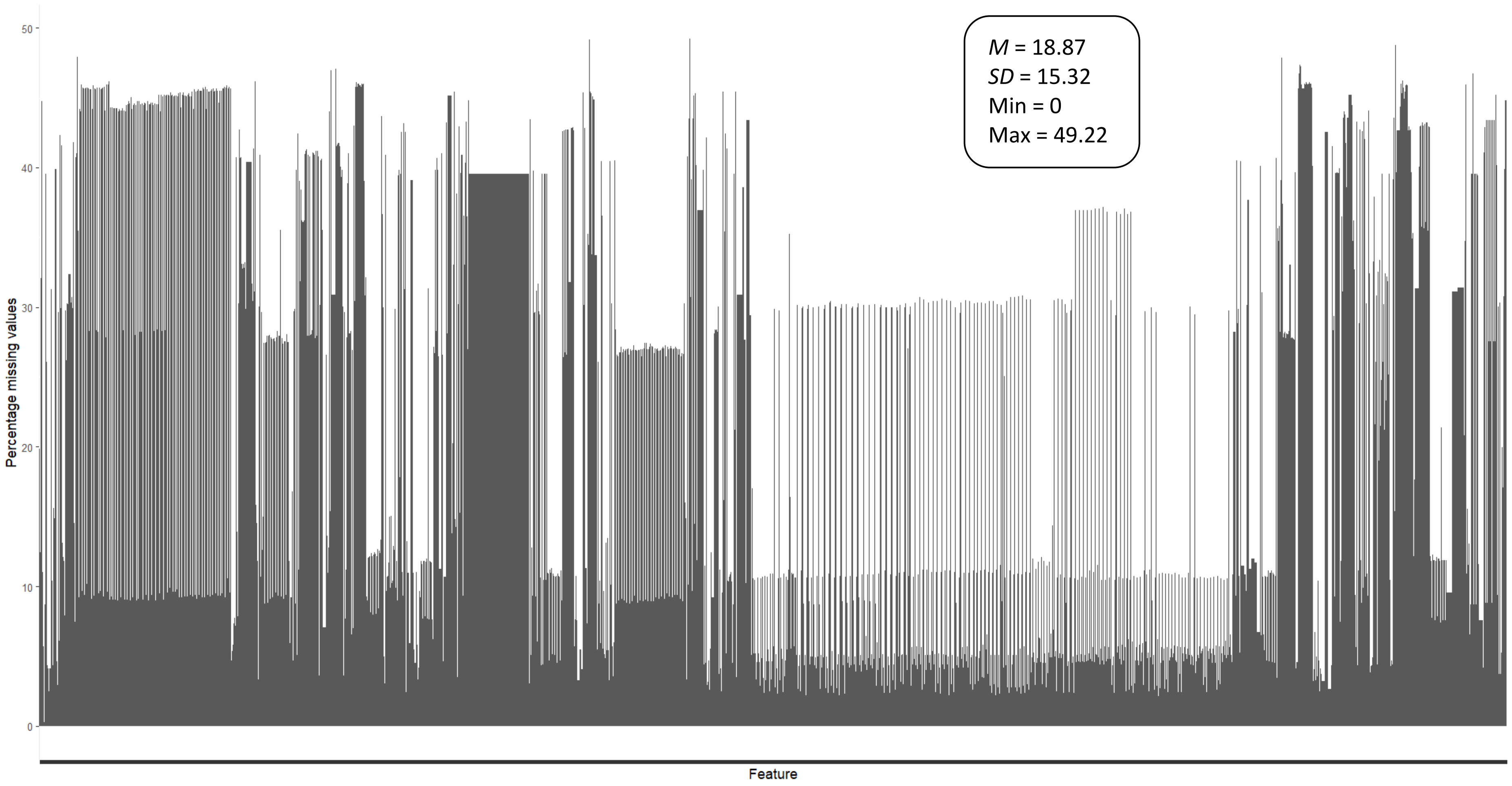

Figure S5A.1. Percentage of missing values for each feature – Unimodal specific exposome (full feature set)

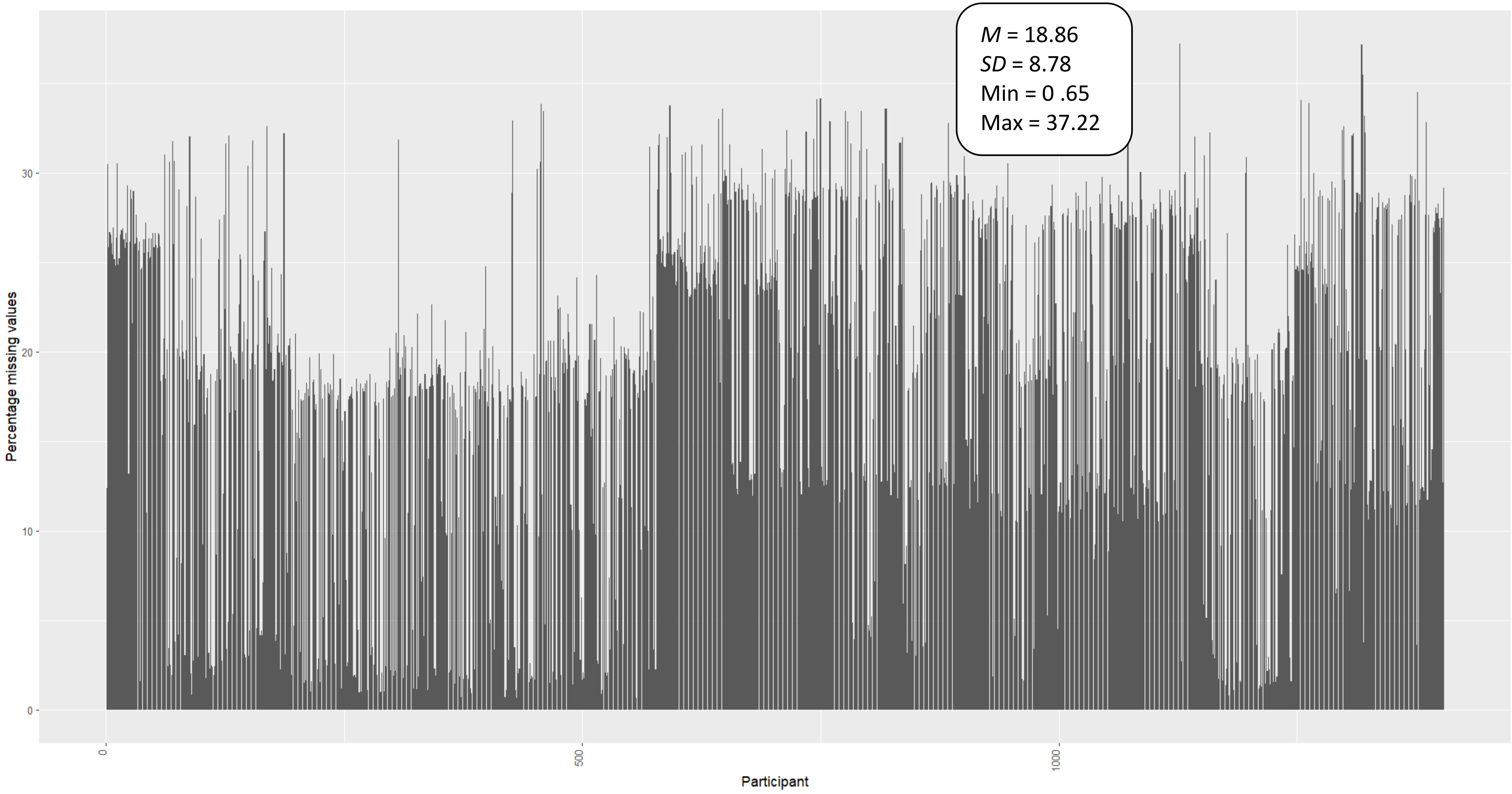

Figure S5A.2. Percentage of missing values for each participant – Unimodal specific exposome (full feature set)

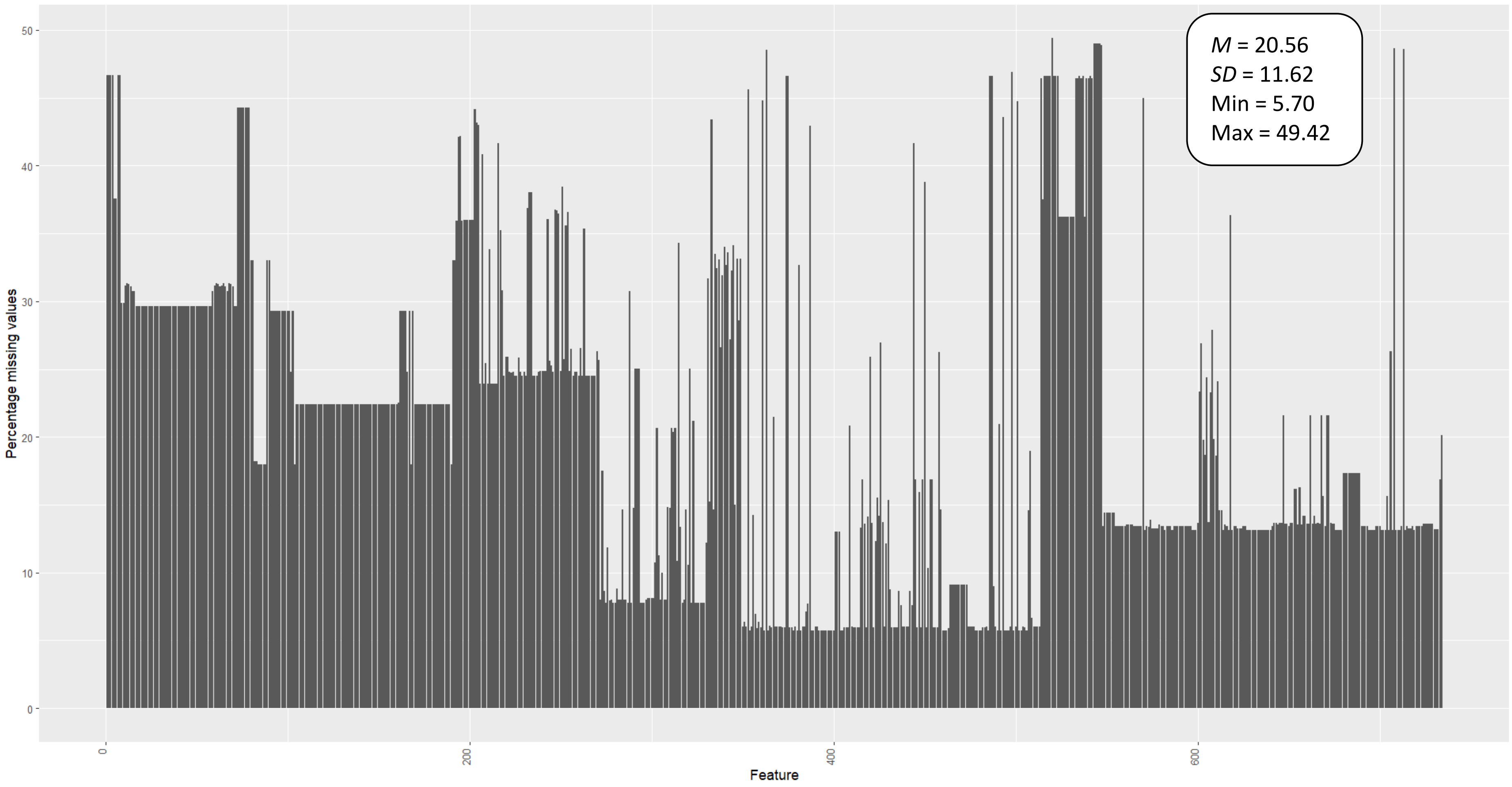

Figure S5B.1. Percentage of missing values for each feature – Unimodal general exposome (full feature set)

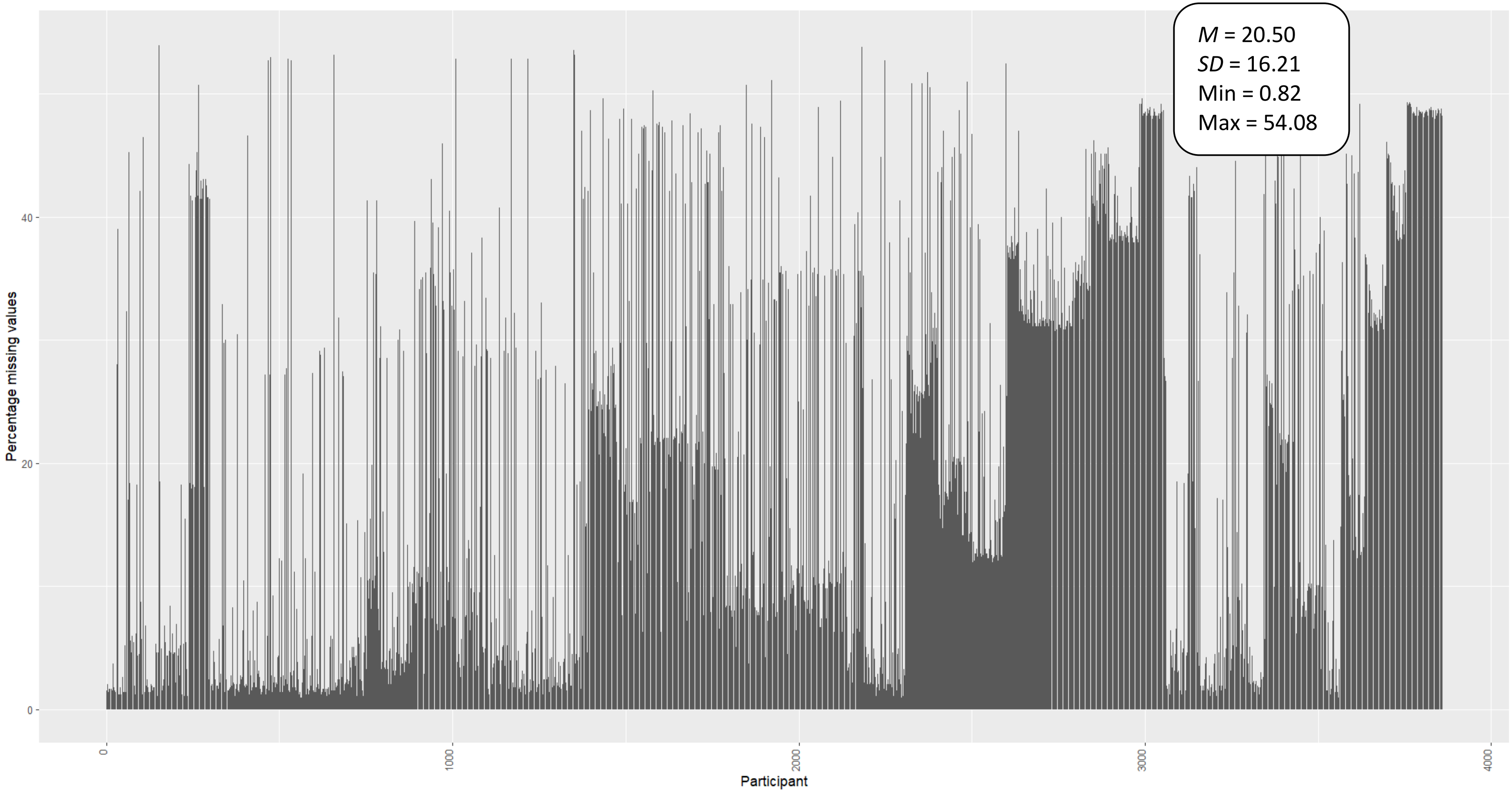

Figure S5B.2. Percentage of missing values for each participant – Unimodal general exposome (full feature set)

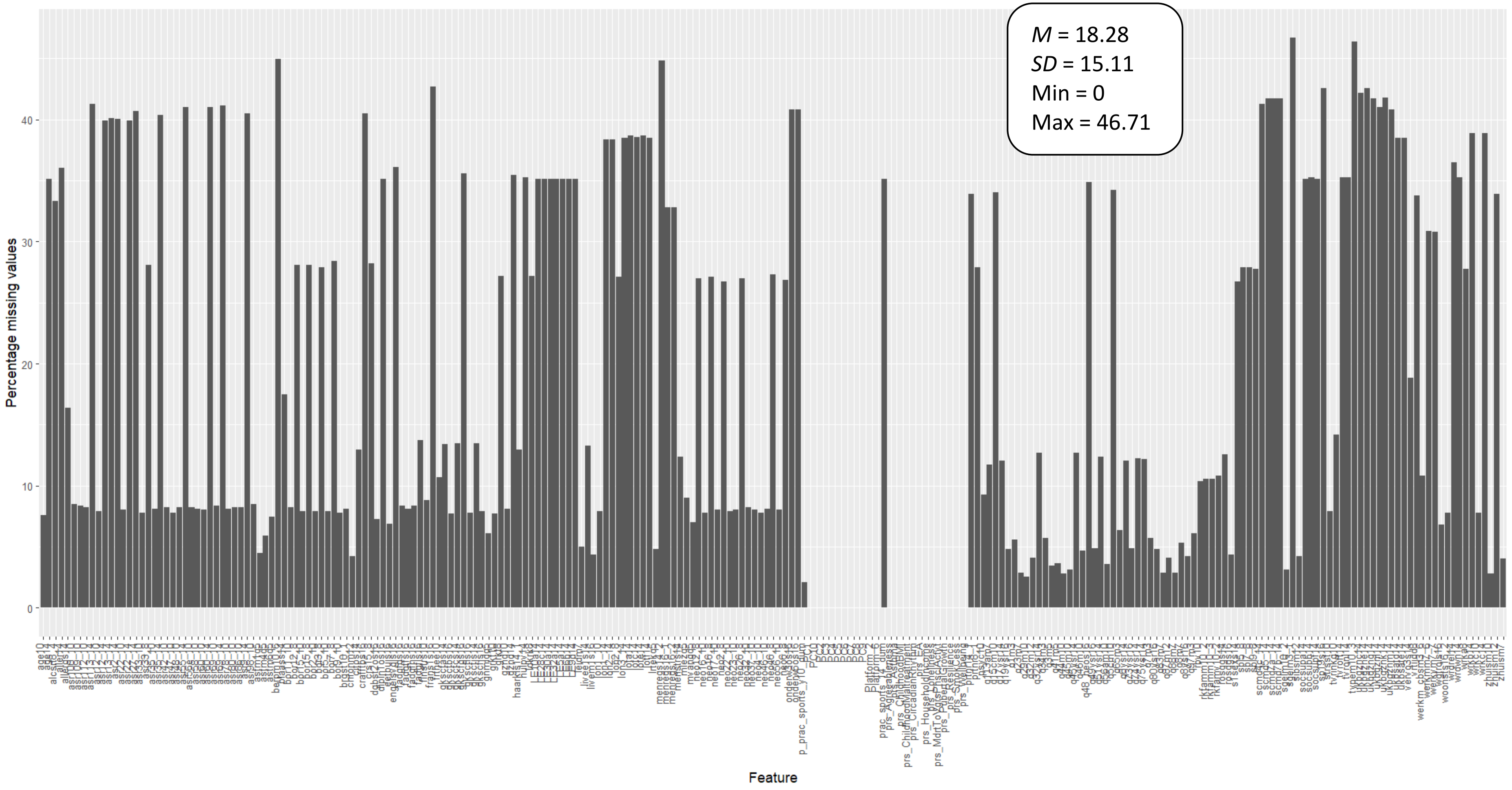

Figure S5C.1. Percentage of missing values for each feature – Multimodal specific exposome + genome (after feature selection). See Table S2 for feature labels.

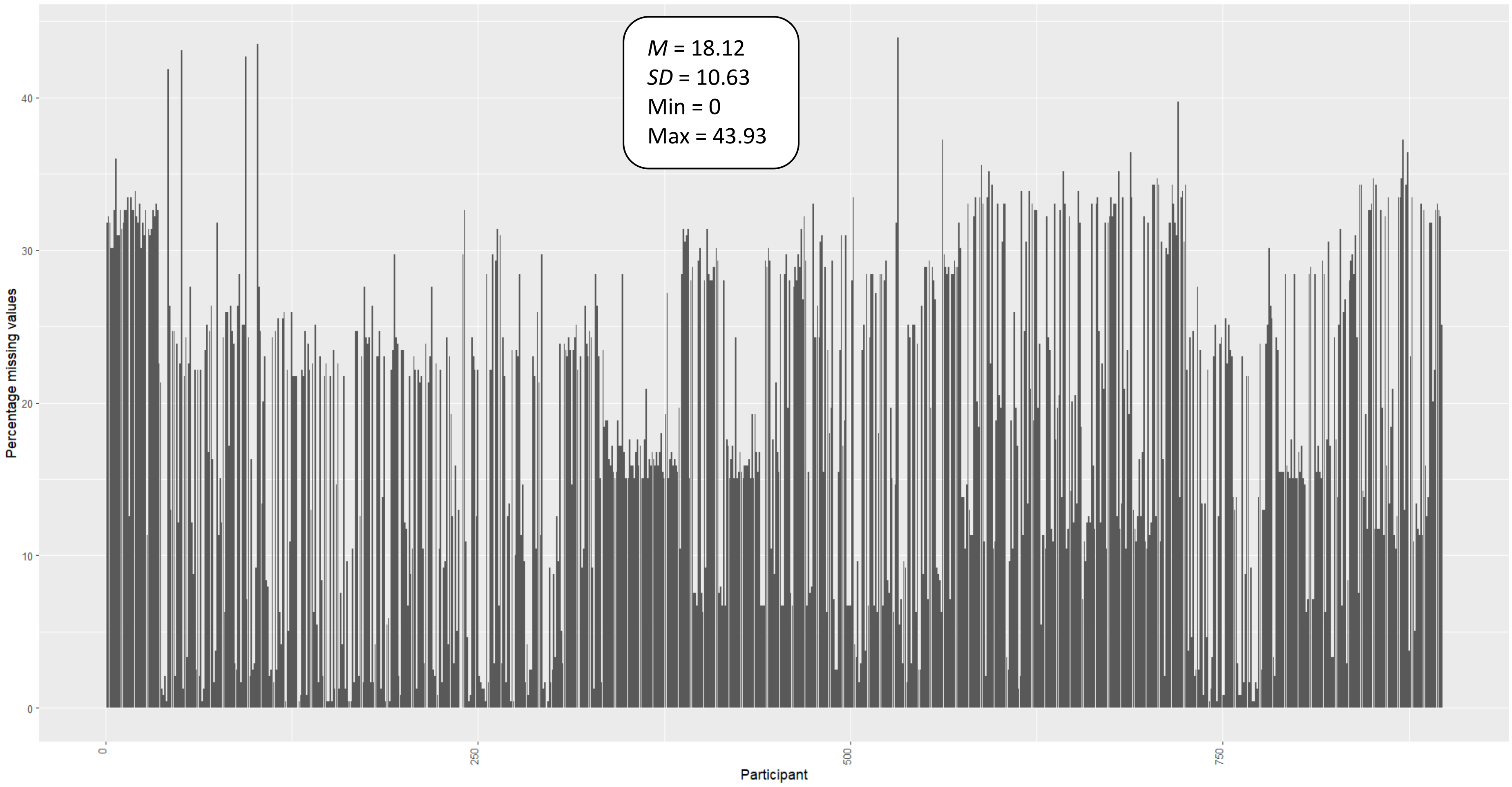

Figure S5C.2. Percentage of missing values for each participant – Multimodal specific exposome + genome (after feature selection).

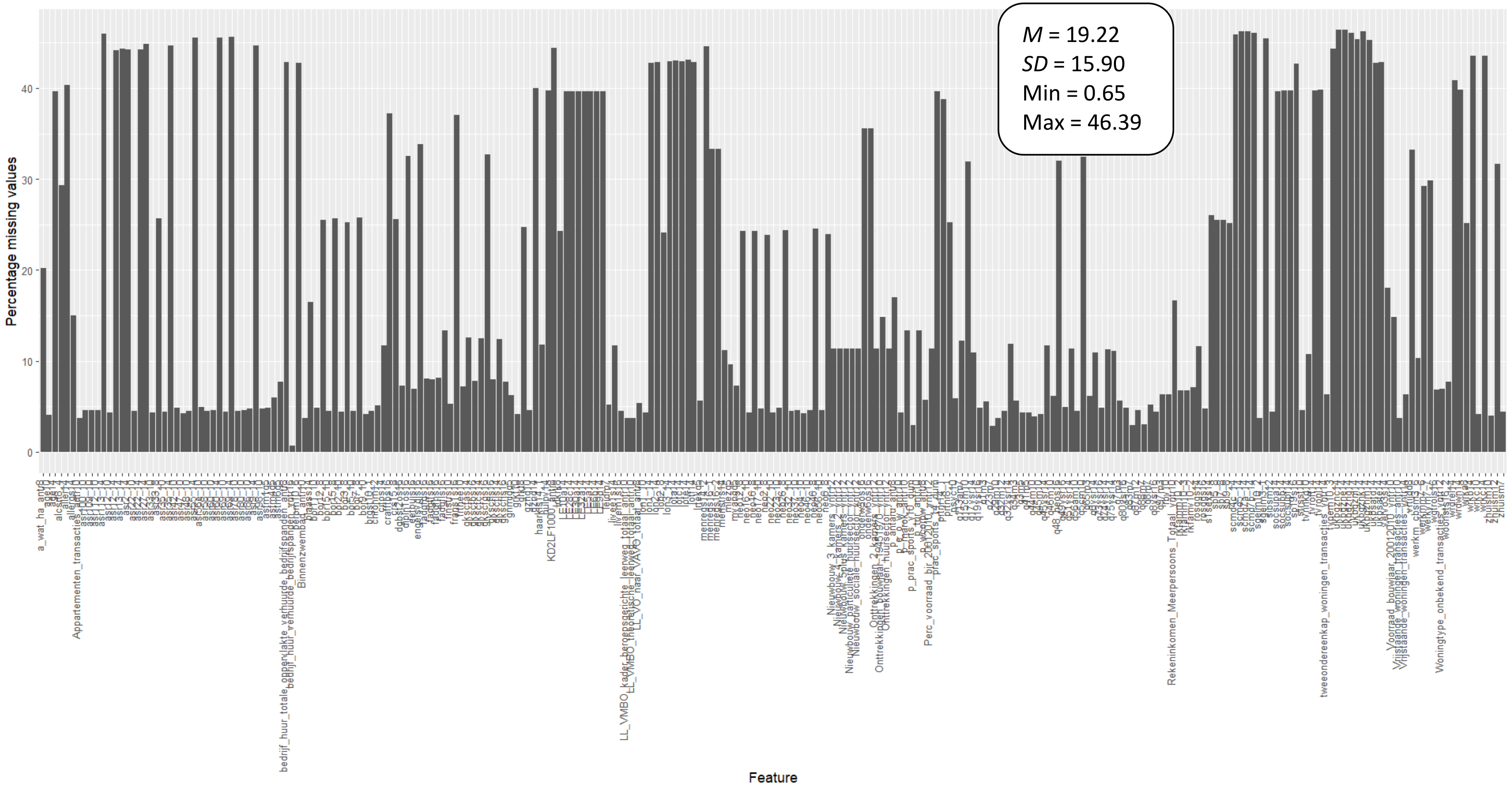

Figure S5D.1. Percentage of missing values for each feature – Multimodal specific exposome + general exposome (after feature selection). See Table S2 for feature labels.

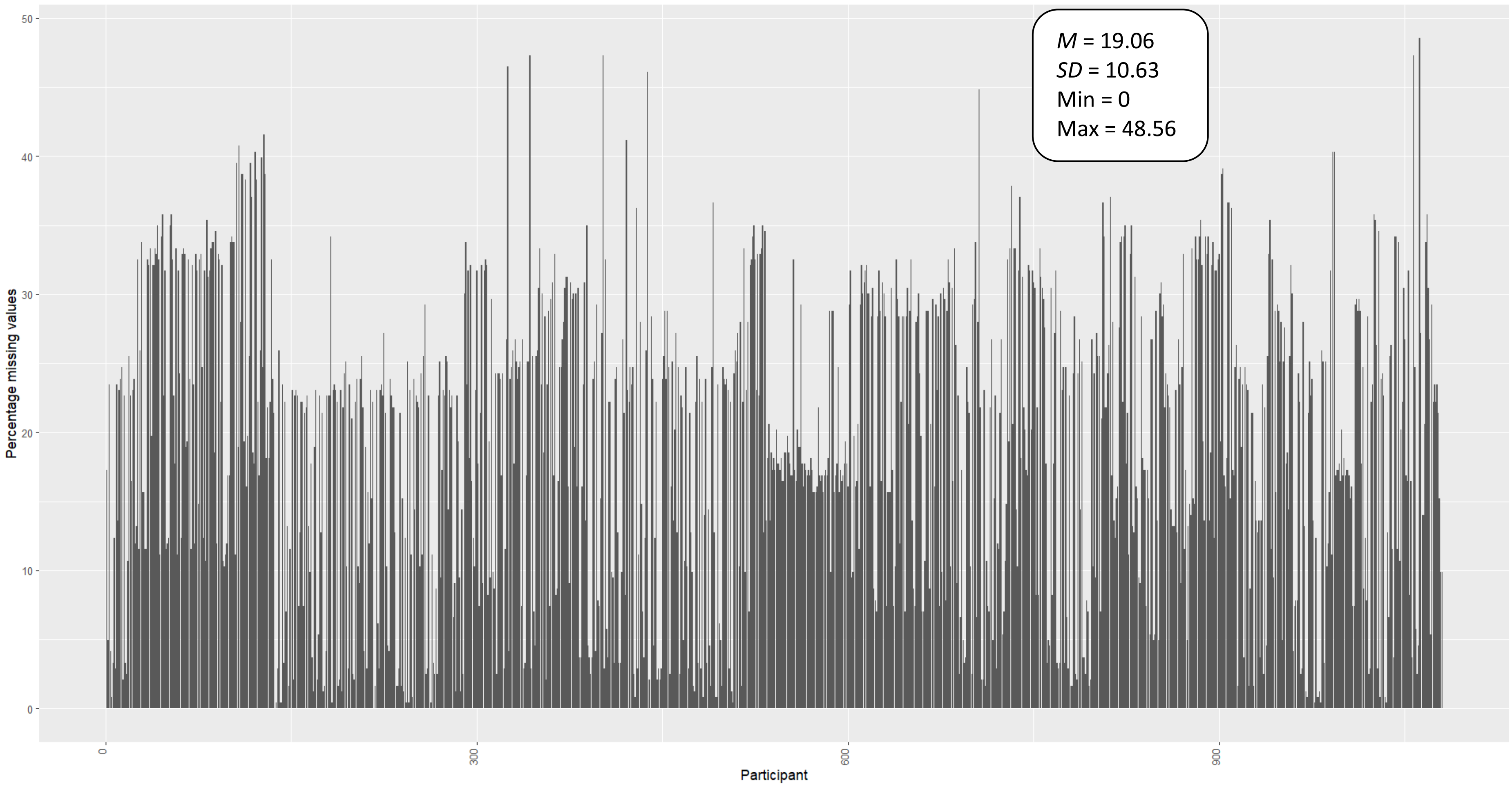

Figure S5D.2. Percentage of missing values for each participant – Multimodal specific exposome + general exposome (after feature selection).

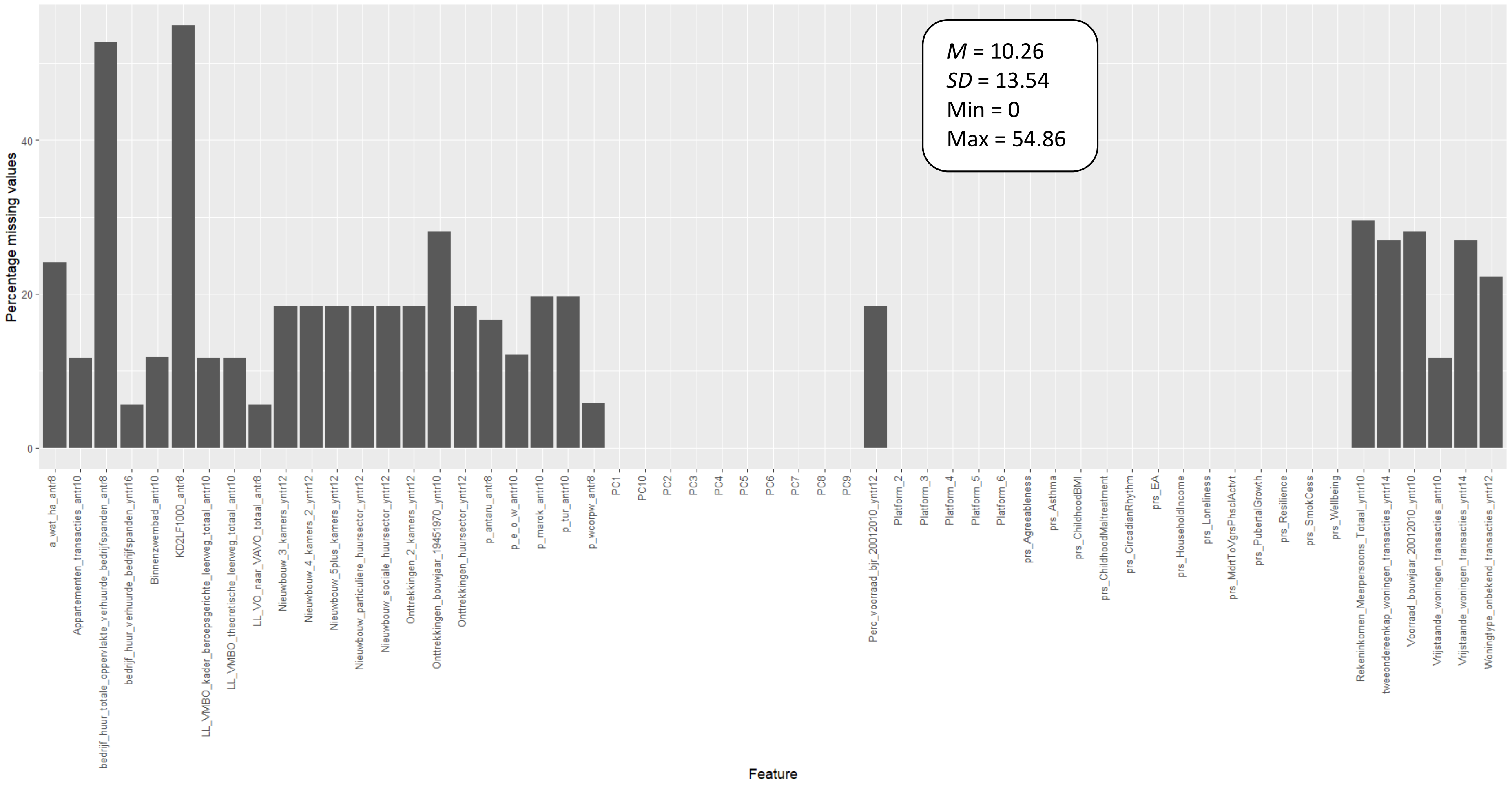

Figure S5E.1. Percentage of missing values for each feature – Multimodal genome + general exposome (after feature selection). See Table S2 for feature labels.

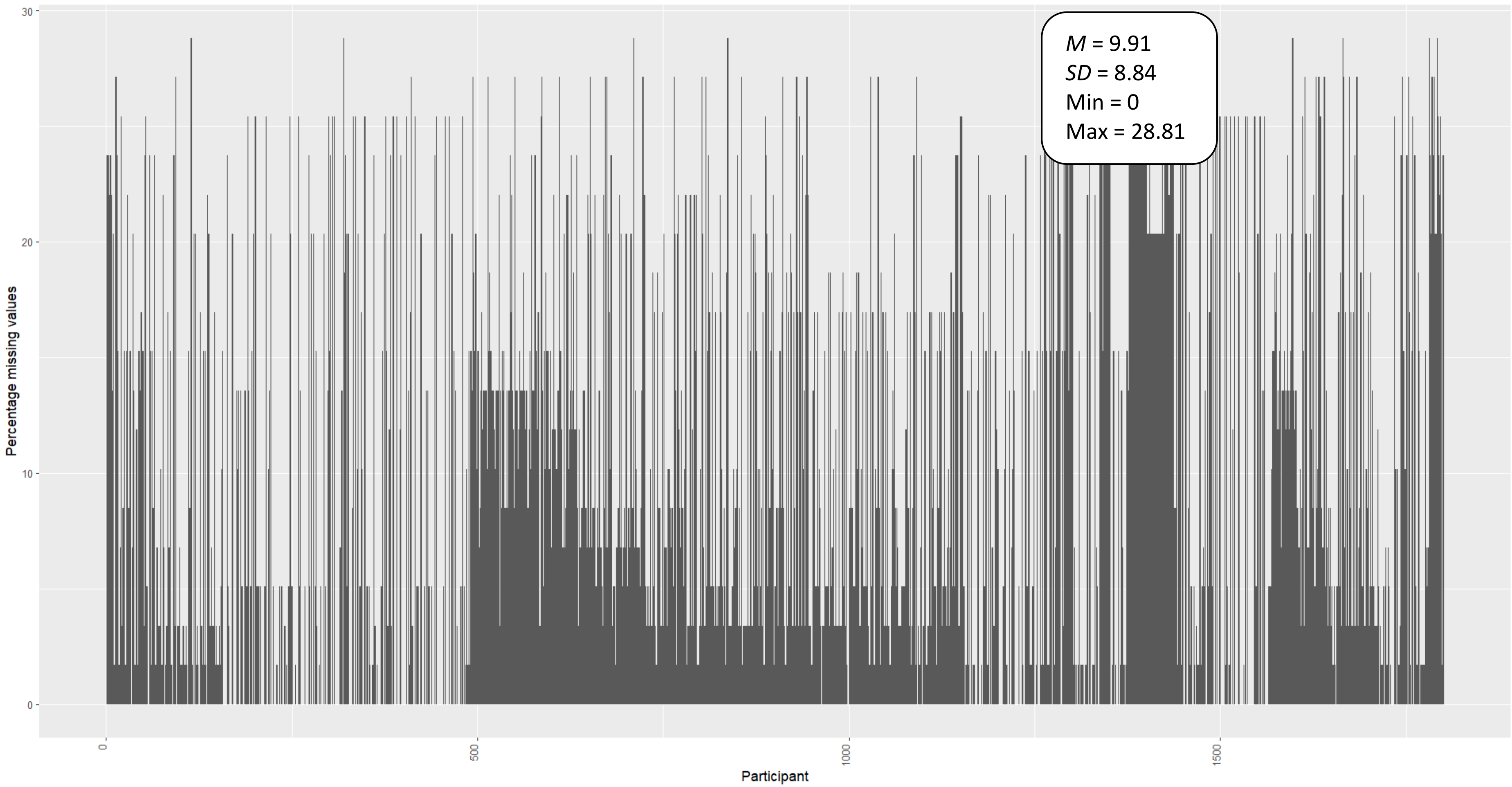

Figure S5E.2. Percentage of missing values for each participant – Multimodal genome + general exposome (after feature selection).

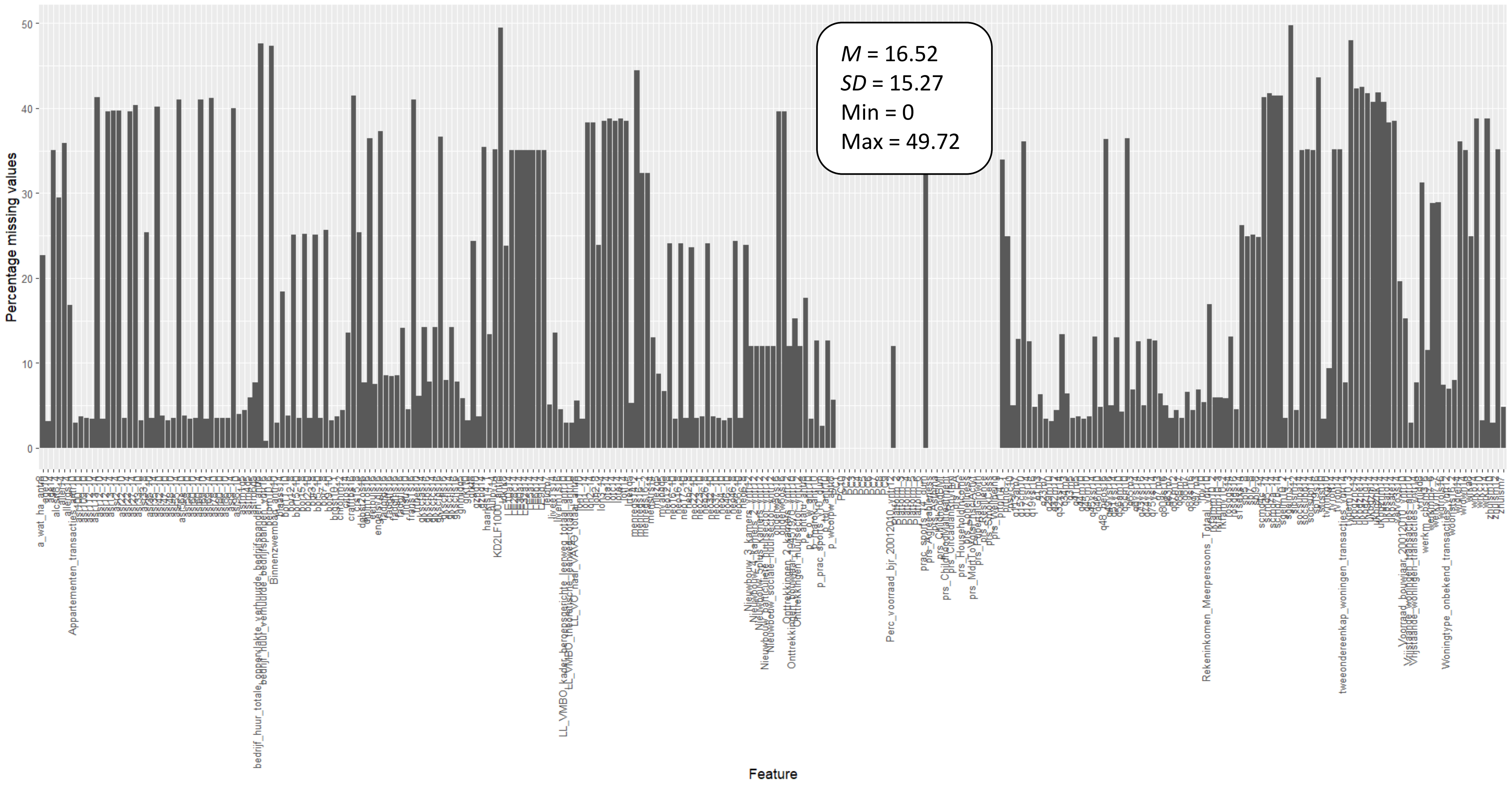

Figure S5F.1. Percentage of missing values for each feature – Multimodal specific exposome + genome + general exposome (after feature selection). See Table S2 for feature labels.

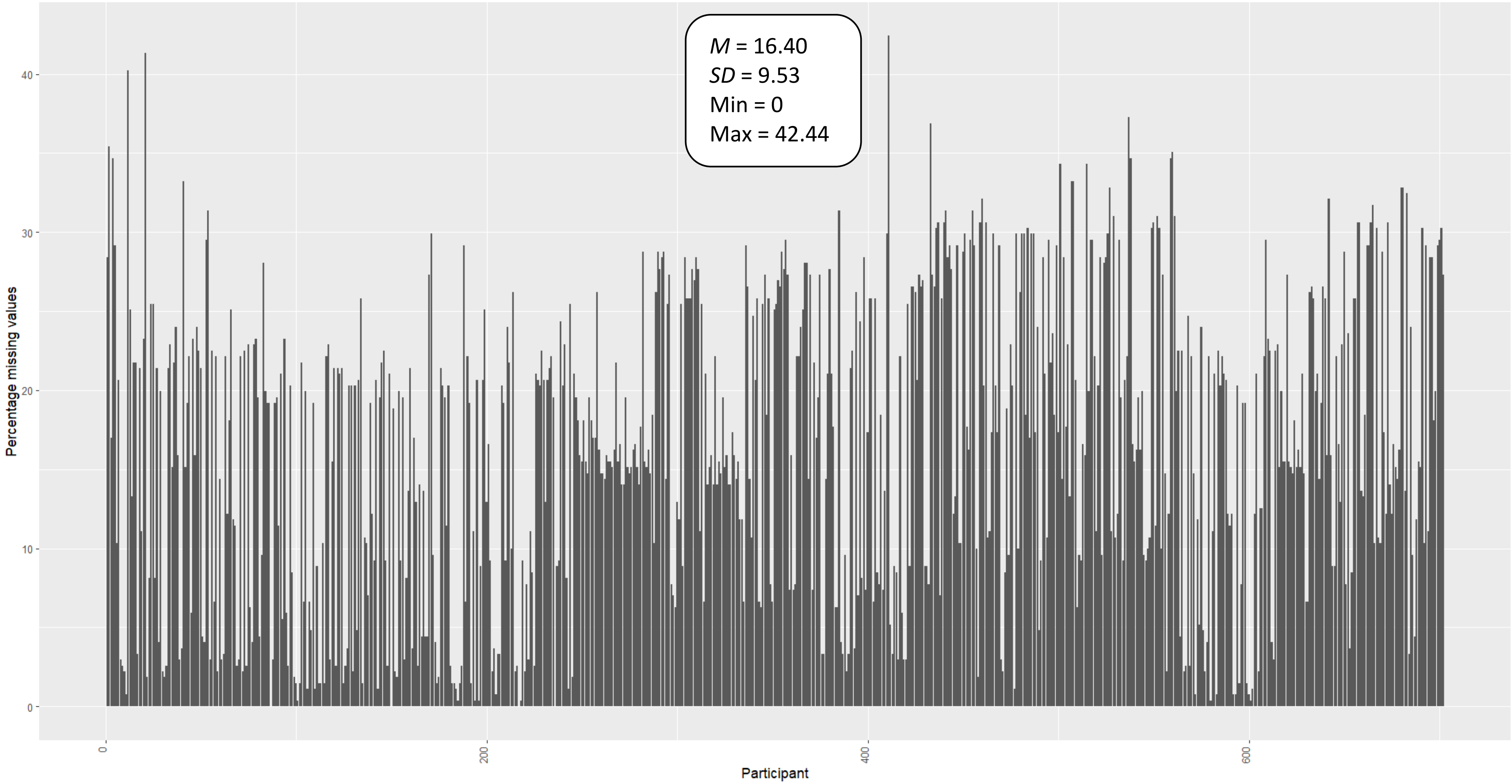

Figure S5F.2. Percentage of missing values for each participant – Multimodal specific exposome + genome + general exposome (after feature selection).

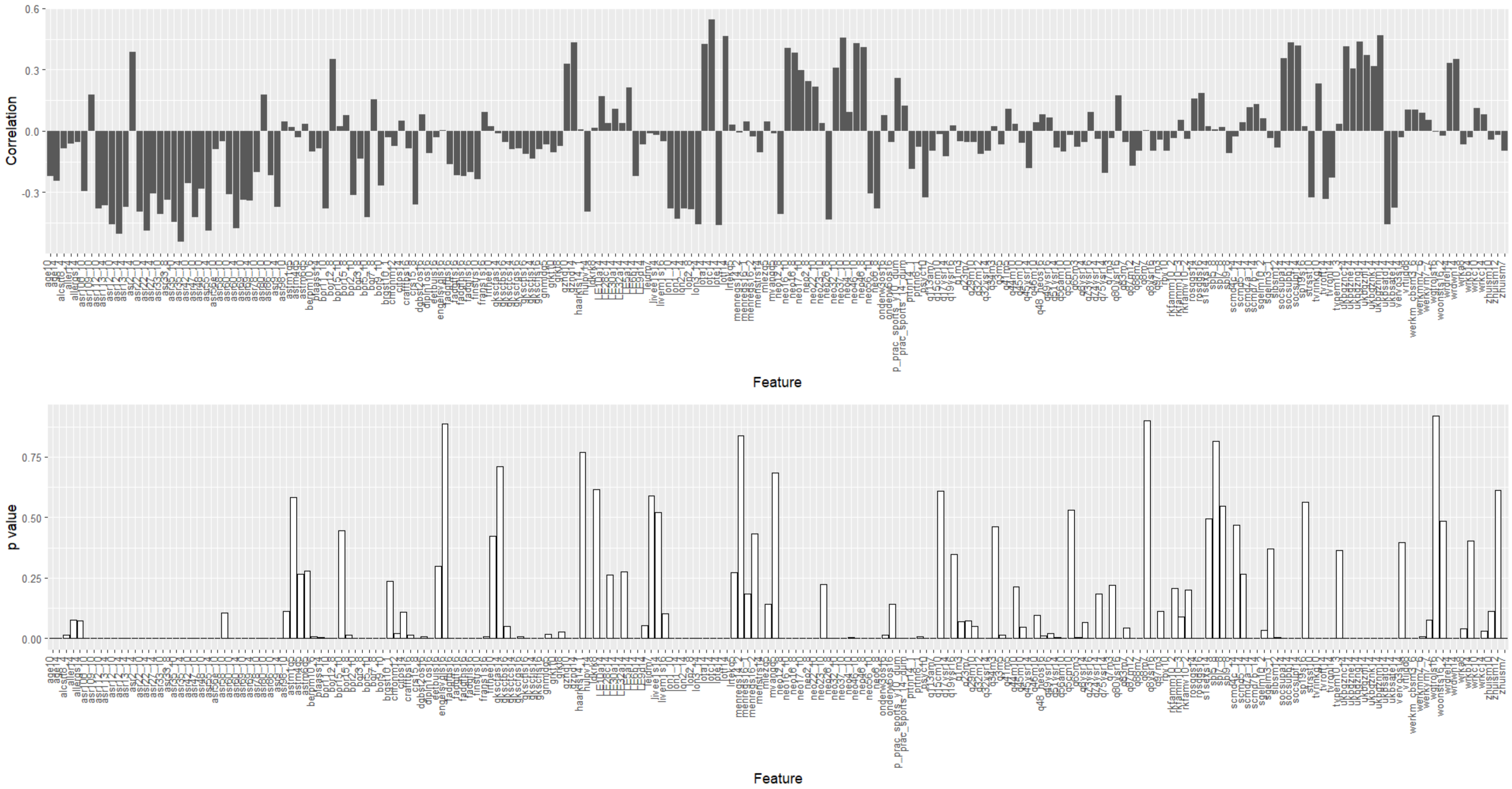

Figure S6. Correlations between specific exposome features (top) and the outcome, and their corresponding *p*-values (bottom).

Supplemental material 1: SHAP values of top 15 features

Unimodal specific exposome model	p. 2
Unimodal genome model	p. 4
Unimodal general exposome model	p. 7
Multimodal: specific exposome + genome	p. 10
Multimodal: specific exposome + general exposome	p. 13
Multimodal: genome + general exposome	p. 16
Multimodal: specific exposome + genome + general exposome	p. 19

Unimodal specific exposome

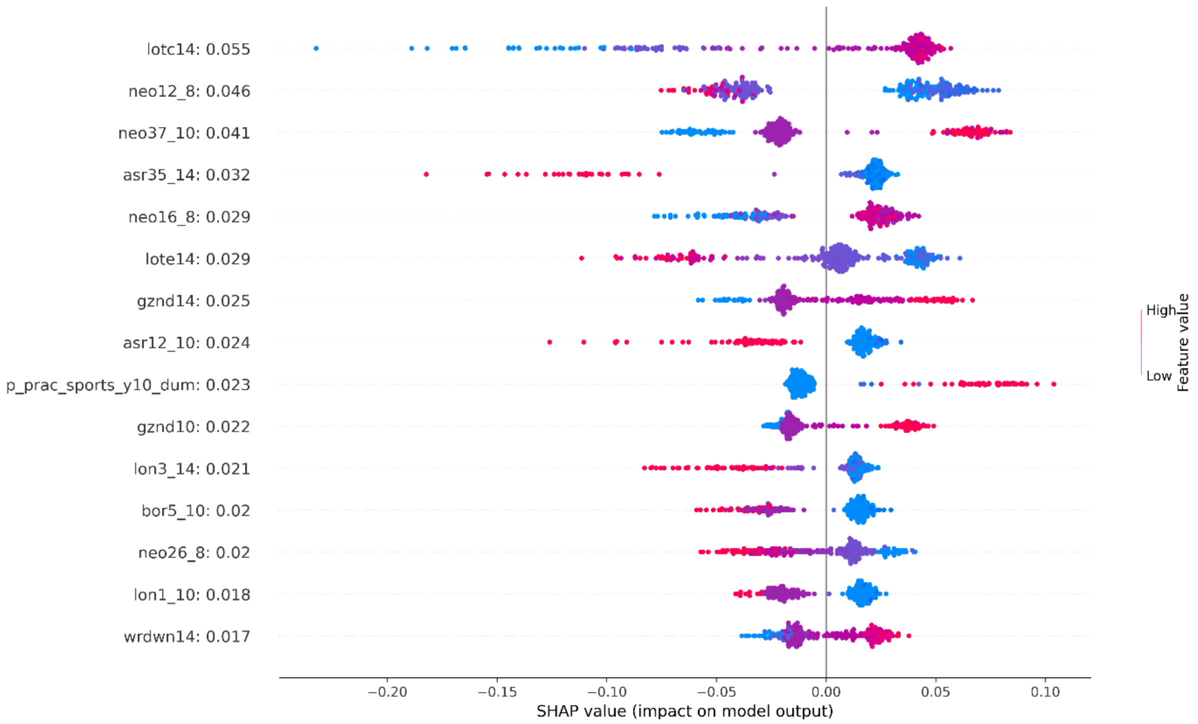

Random forest (RF)

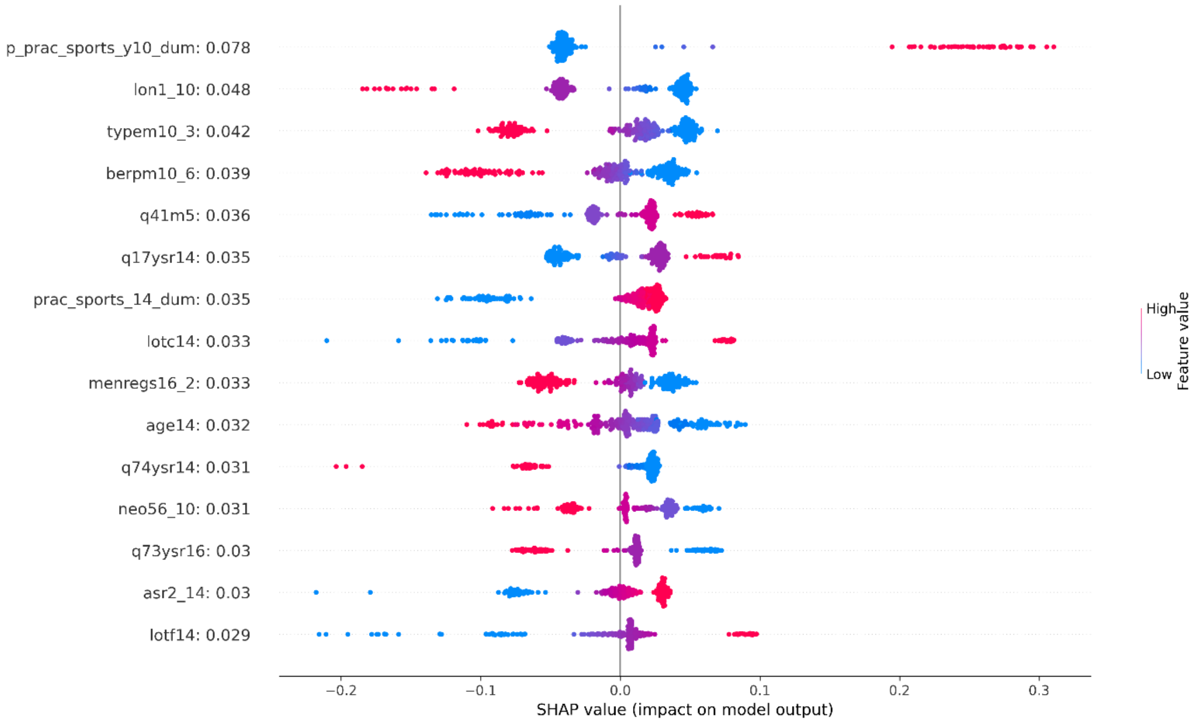

Support vector machine (SVM)

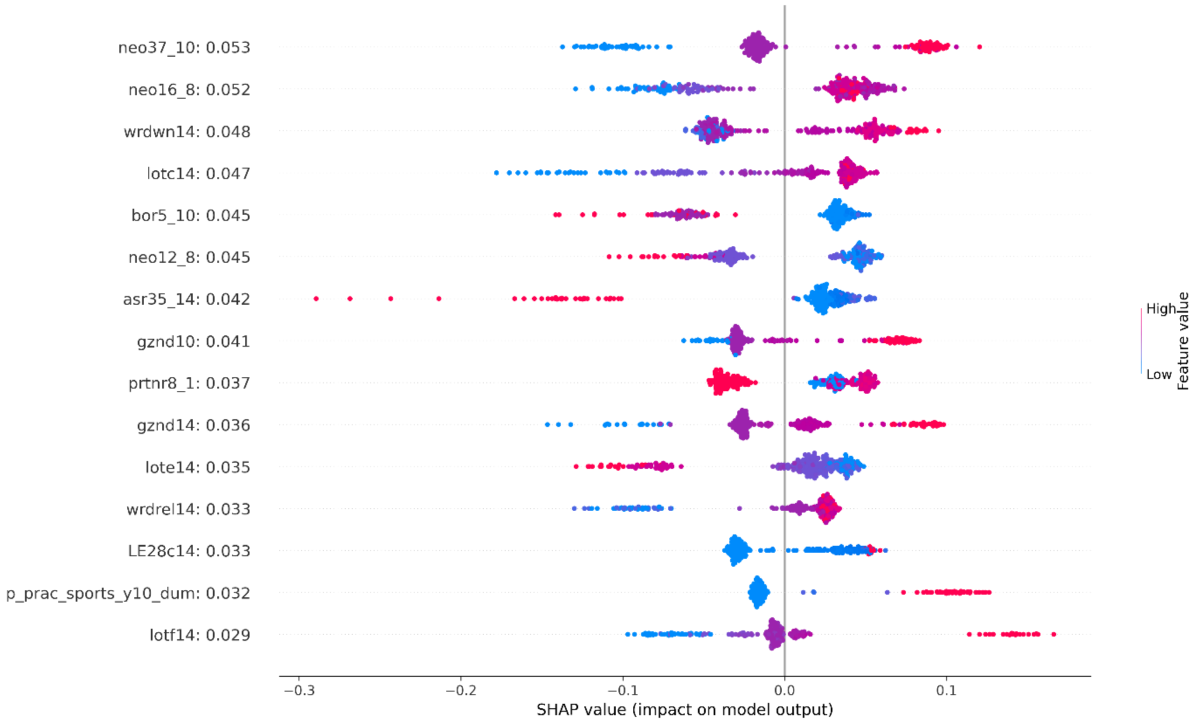

Extreme gradient boost (XGB)

Unimodal genome

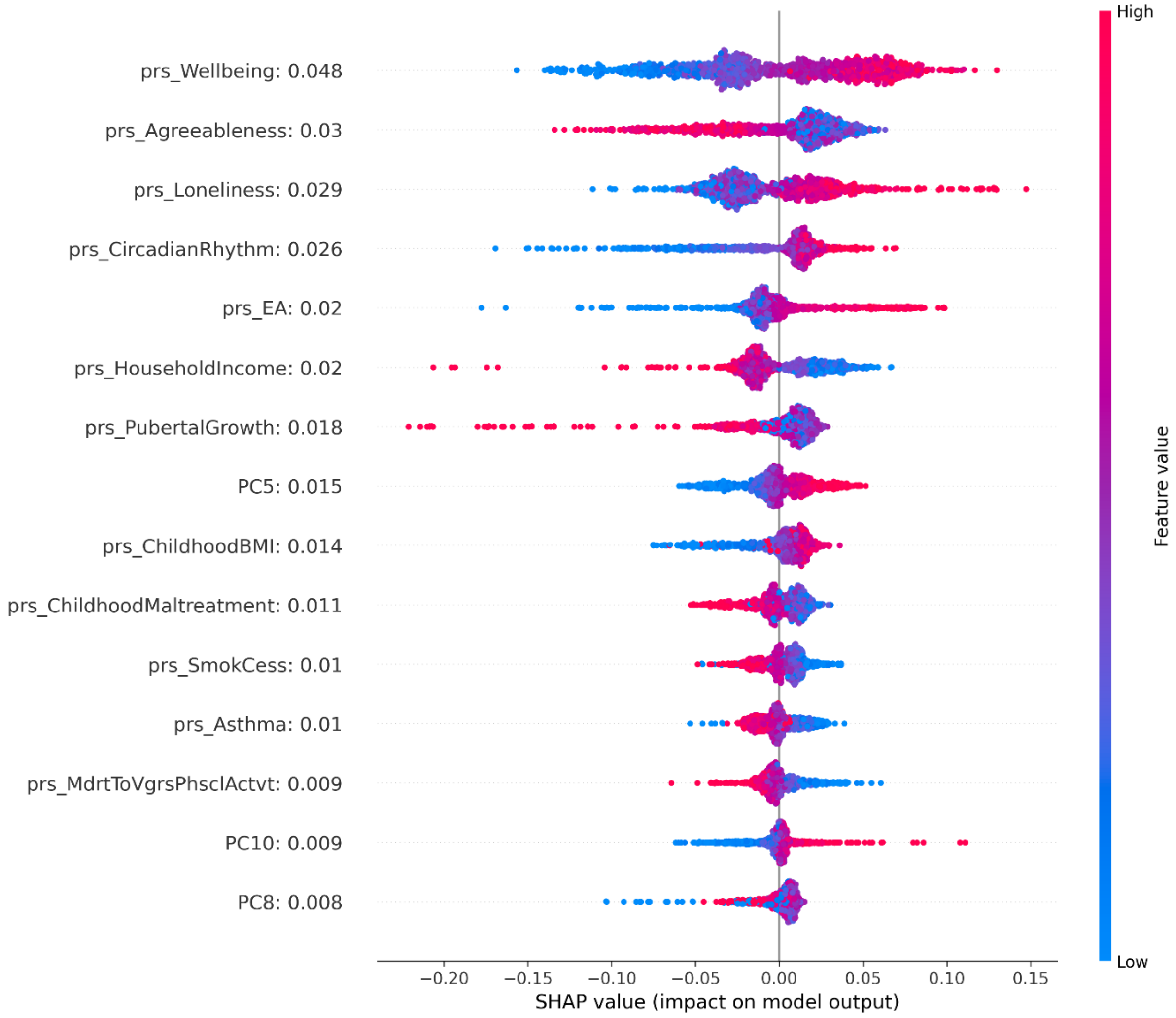

RF

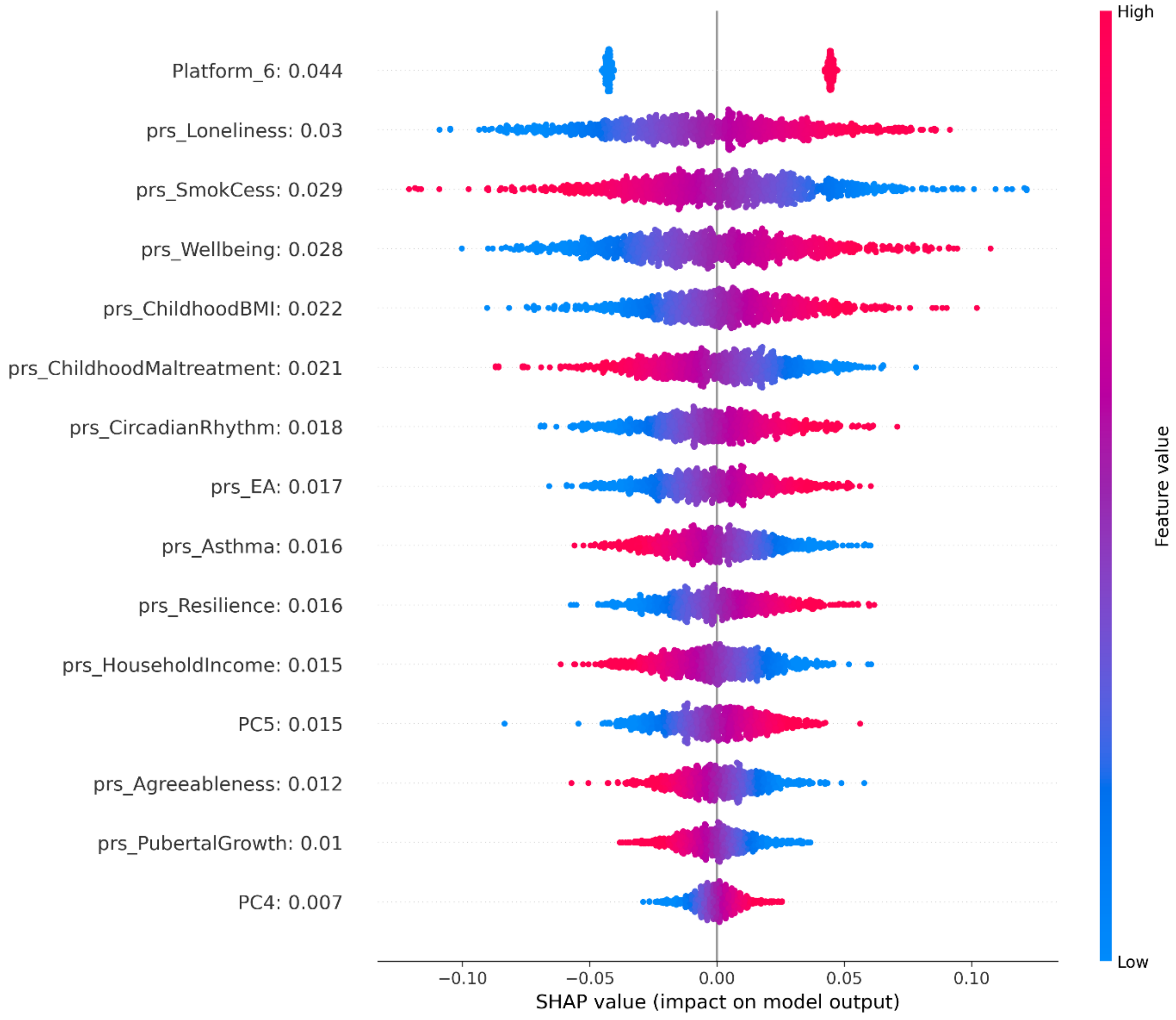

Platform_6: 0.044
prs_Loneliness: 0.03
prs_SmokCess: 0.029
prs_Wellbeing: 0.028
prs_ChildhoodBMI: 0.022
prs_ChildhoodMaltreatment: 0.021
prs_CircadianRhythm: 0.018
prs_EA: 0.017
prs_Asthma: 0.016
prs_Resilience: 0.016
prs_HouseholdIncome: 0.015
PC5: 0.015
prs_Agreeableness: 0.012
prs_PubertalGrowth: 0.01
PC4: 0.007
−0.10
−0.05
0.00
0.05
0.10
SHAP value (impact on model output)
High
Feature value
Low

SVM

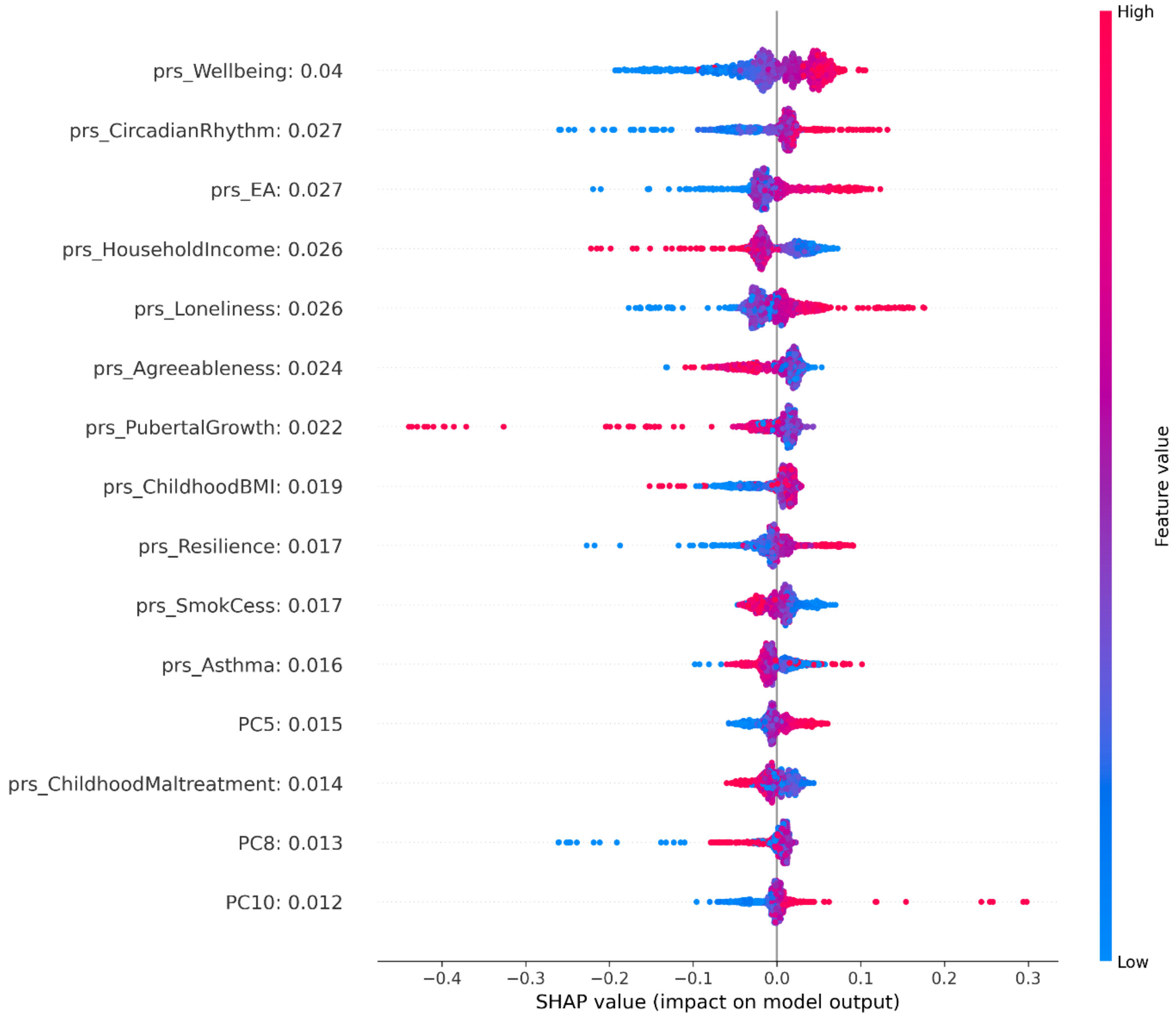

prs_Wellbeing: 0.04
prs_CircadianRhythm: 0.027
prs_EA: 0.027
prs_HouseholdIncome: 0.026
prs_Loneliness: 0.026
prs_Agreeableness: 0.024
prs_PubertalGrowth: 0.022
prs_ChildhoodBMI: 0.019
prs_Resilience: 0.017
prs_SmokCess: 0.017
prs_Asthma: 0.016
PC5: 0.015
prs_ChildhoodMaltreatment: 0.014
PC8: 0.013
PC10: 0.012
−0.4
−0.3
−0.2
−0.1
0.0
0.1
0.2
0.3
SHAP value (impact on model output)
High
Feature value
Low

XGB

Unimodal general exposome

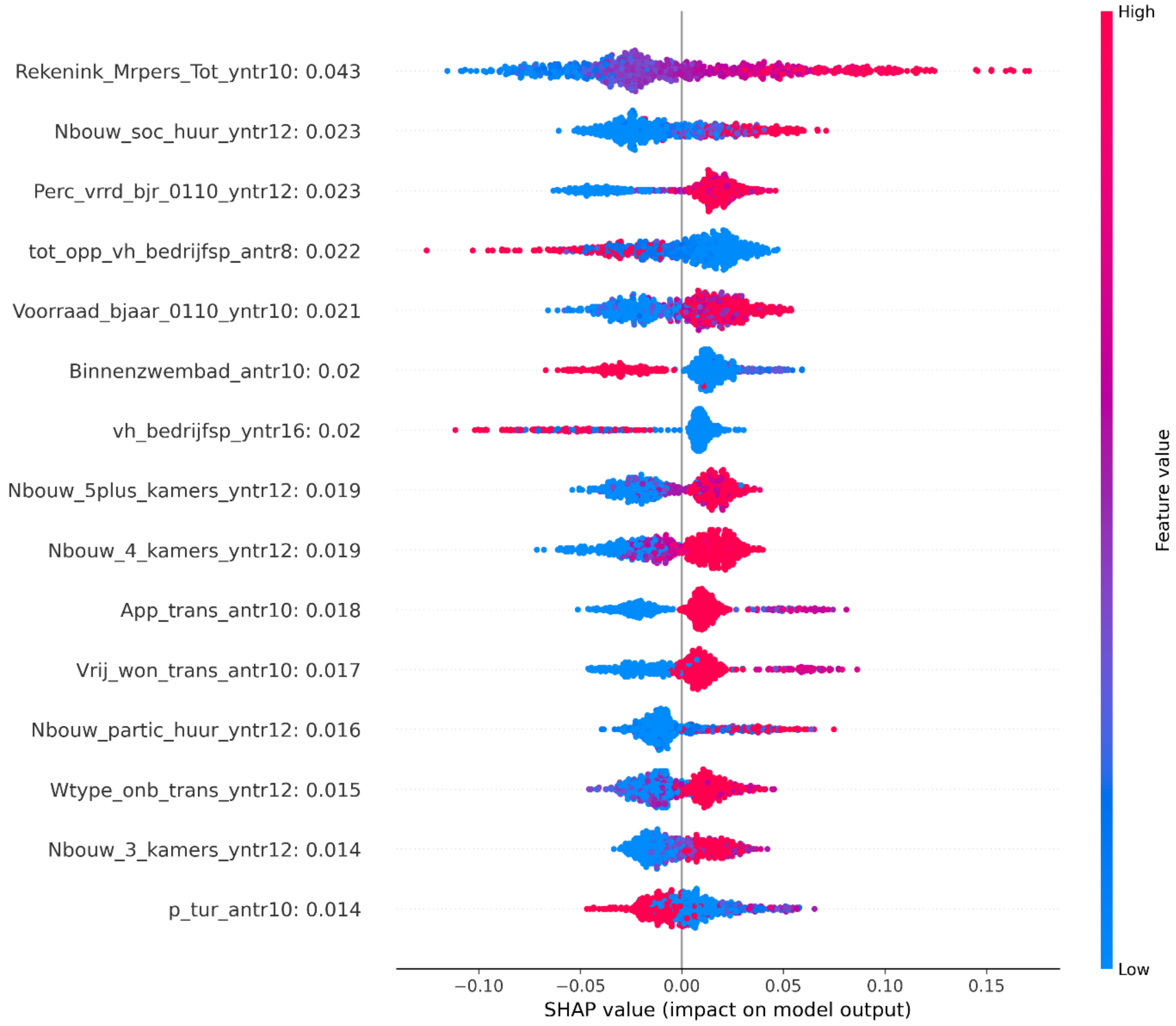

RF

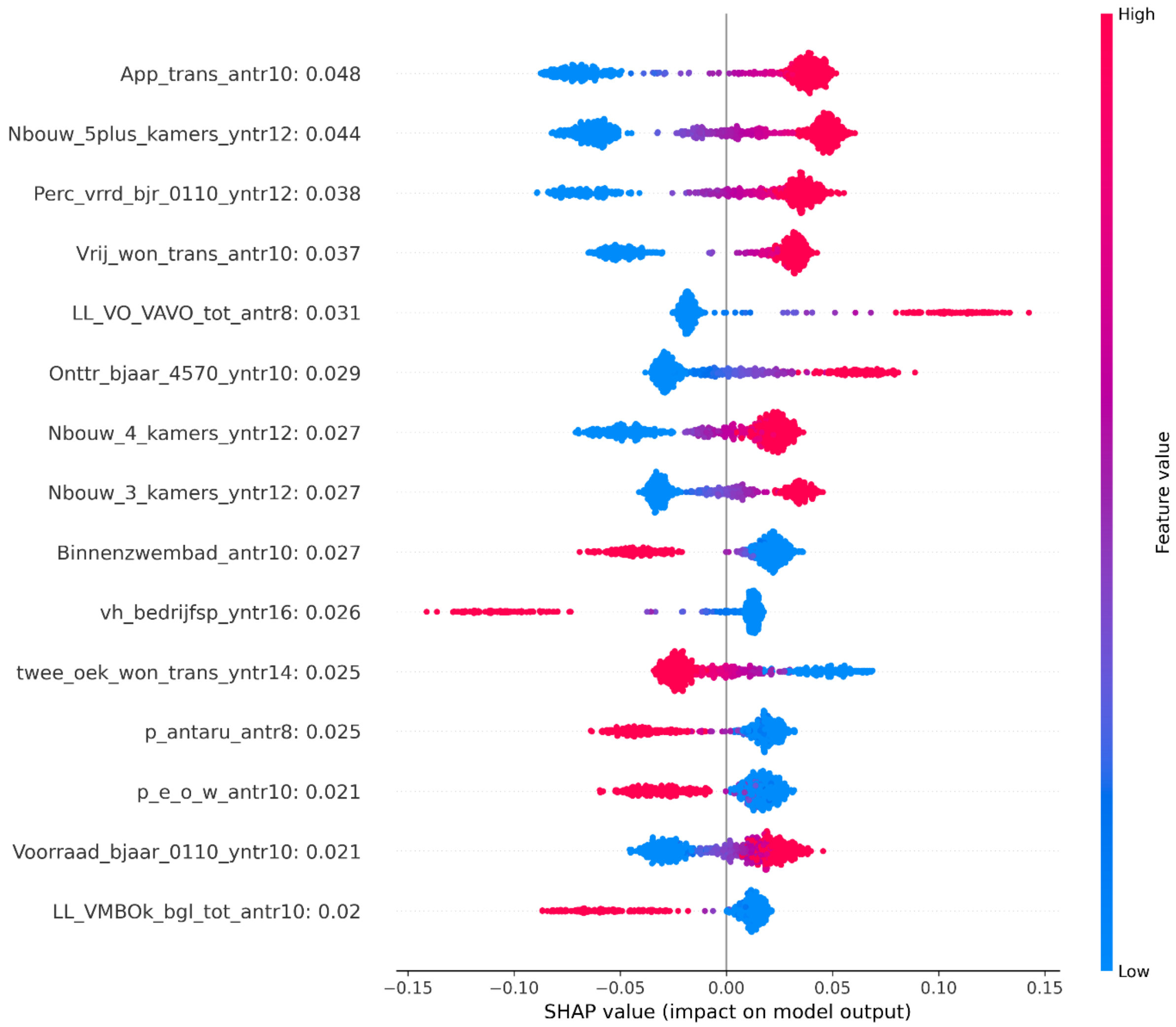

App_trans_antr10: 0.048
Nbouw_5plus_kamers_yntr12: 0.044
Perc_vrrd_bjr_0110_yntr12: 0.038
Vrij_won_trans_antr10: 0.037
LL_VO_VAVO_tot_antr8: 0.031
Onttr_bjaar_4570_yntr10: 0.029
Nbouw_4_kamers_yntr12: 0.027
Nbouw_3_kamers_yntr12: 0.027
Binnenzwembad_antr10: 0.027
vh_bedrijfsp_yntr16: 0.026
twee_oek_won_trans_yntr14: 0.025
p_antaru_antr8: 0.025
p_e_o_w_antr10: 0.021
Voorraad_bjaar_0110_yntr10: 0.021
LL_VMBOk_bgl_tot_antr10: 0.02
−0.15
−0.10
−0.05
0.00
0.05
0.10
0.15
SHAP value (impact on model output)
High
Low
Feature value

SVM

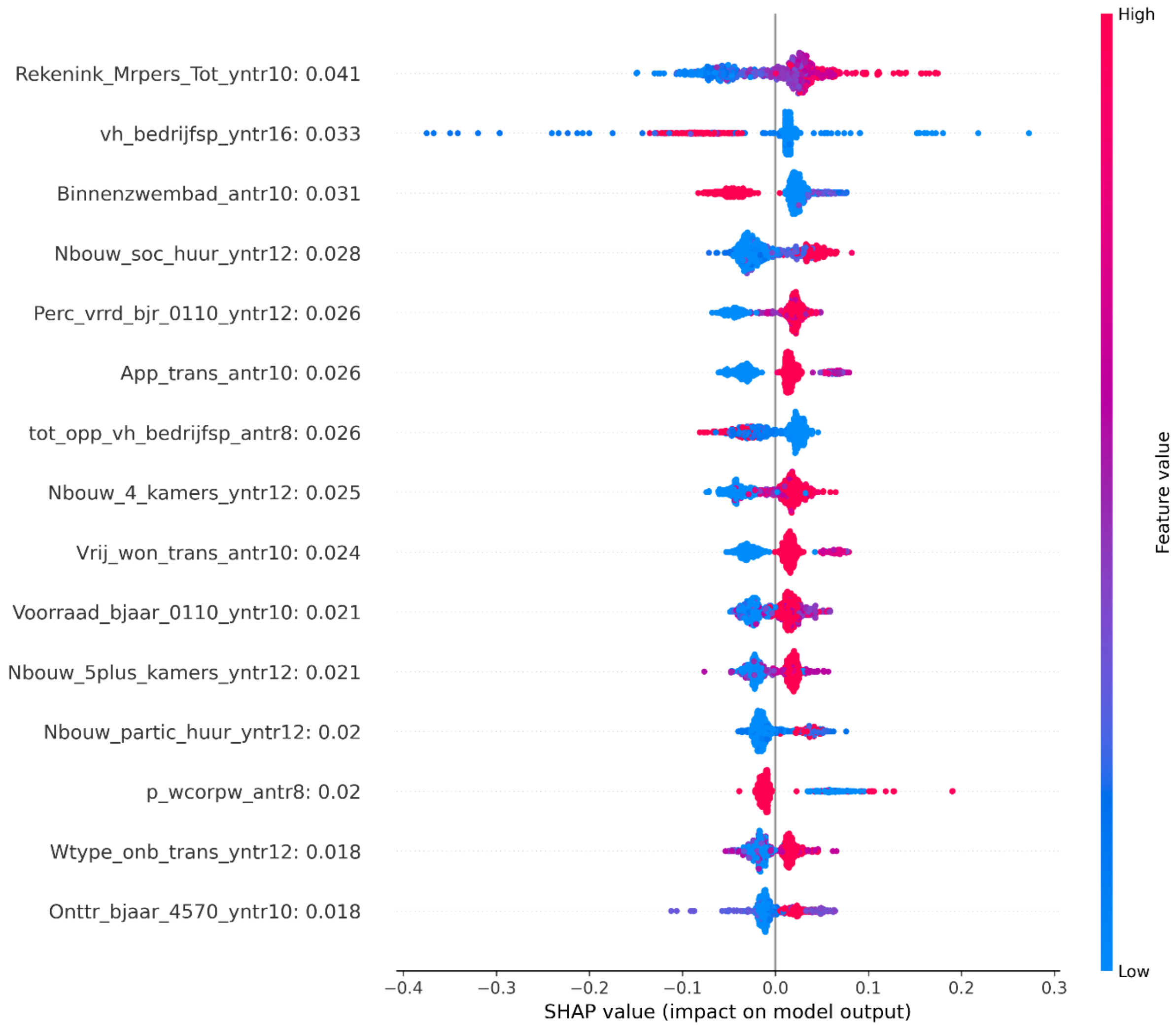

Rekenink_Mrpers_Tot_yntr10: 0.041
vh_bedrijfsp_yntr16: 0.033
Binnenzwembad_antr10: 0.031
Nbouw_soc_huur_yntr12: 0.028
Perc_vrrd_bjr_0110_yntr12: 0.026
App_trans_antr10: 0.026
tot_opp_vh_bedrijfsp_antr8: 0.026
Nbouw_4_kamers_yntr12: 0.025
Vrij_won_trans_antr10: 0.024
Voorraad_bjaar_0110_yntr10: 0.021
Nbouw_5plus_kamers_yntr12: 0.021
Nbouw_partic_huur_yntr12: 0.02
p_wcorpw_antr8: 0.02
Wtype_onb_trans_yntr12: 0.018
Onttr_bjaar_4570_yntr10: 0.018
−0.4
−0.3
−0.2
−0.1
0.0
0.1
0.2
0.3
SHAP value (impact on model output)
High
Low
Feature value

XGB

Multimodal: specific exposome + genome

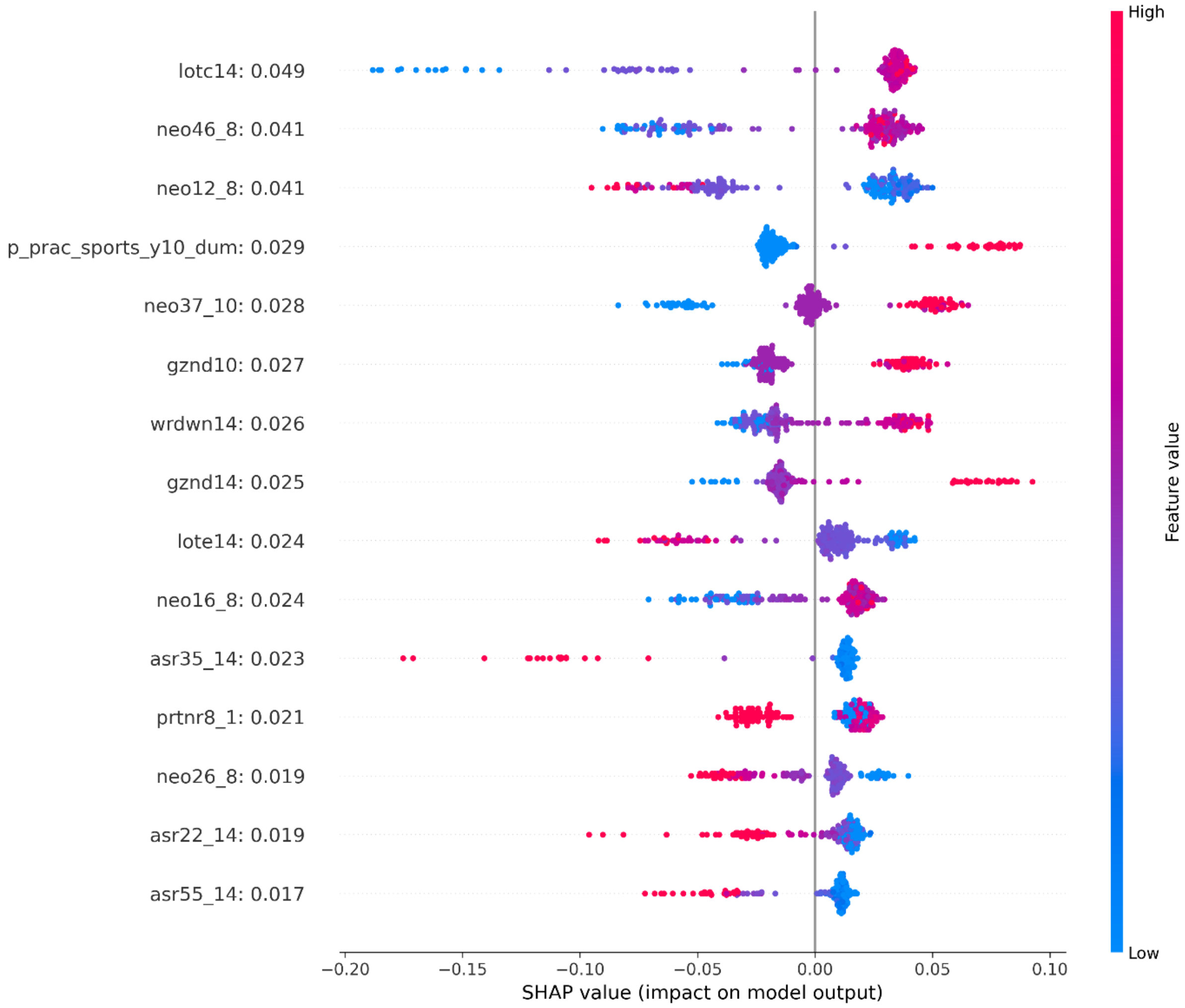

RF

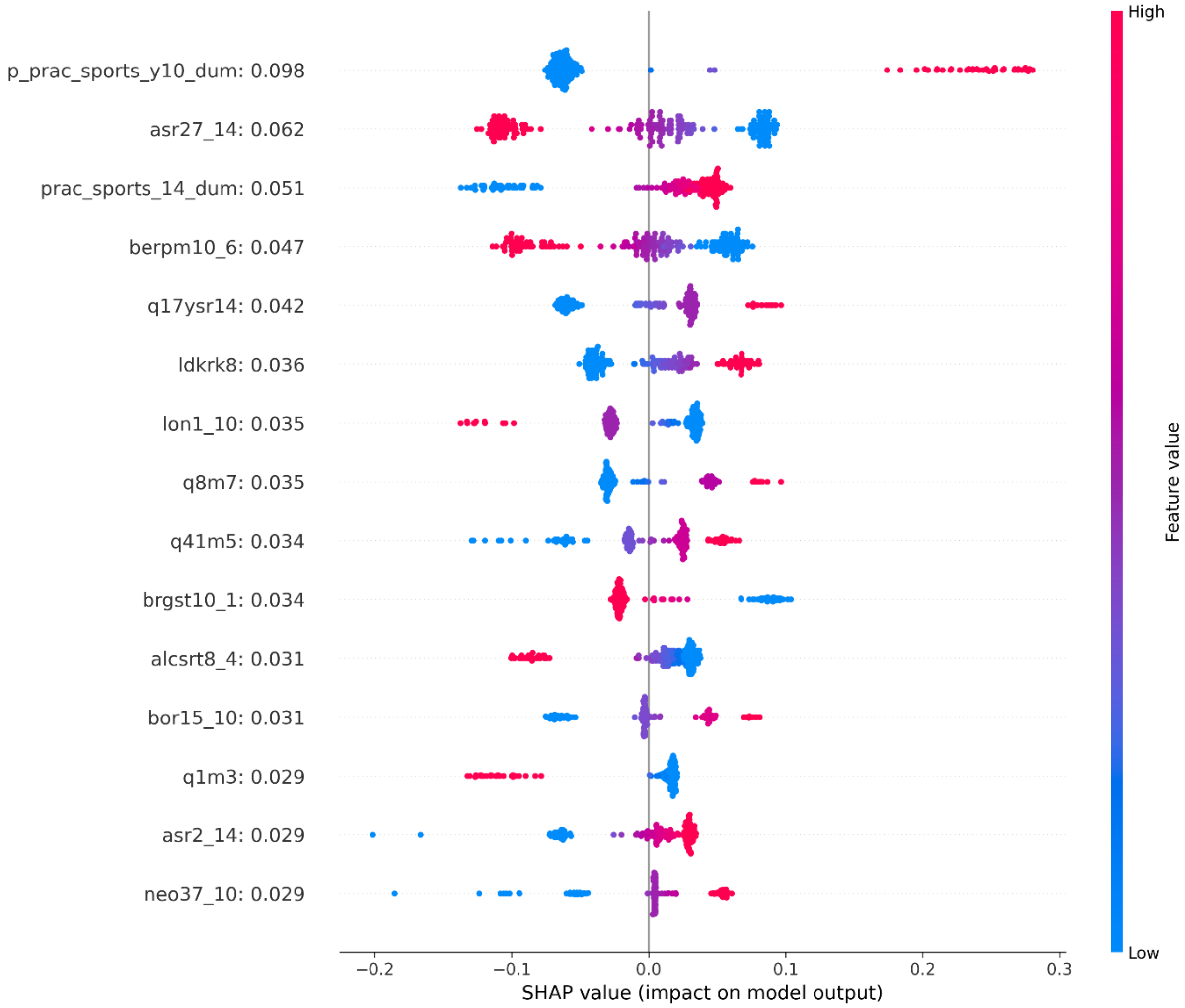
p_prac_sports_y10_dum: 0.098
asr27_14: 0.062
prac_sports_14_dum: 0.051
berpm10_6: 0.047
q17ysr14: 0.042
ldkrk8: 0.036
lon1_10: 0.035
q8m7: 0.035
q41m5: 0.034
brgst10_1: 0.034
alcsrt8_4: 0.031
bor15_10: 0.031
q1m3: 0.029
asr2_14: 0.029
neo37_10: 0.029
−0.2
−0.1
0.0
0.1
0.2
0.3
SHAP value (impact on model output)
High
Feature value
Low

SVM

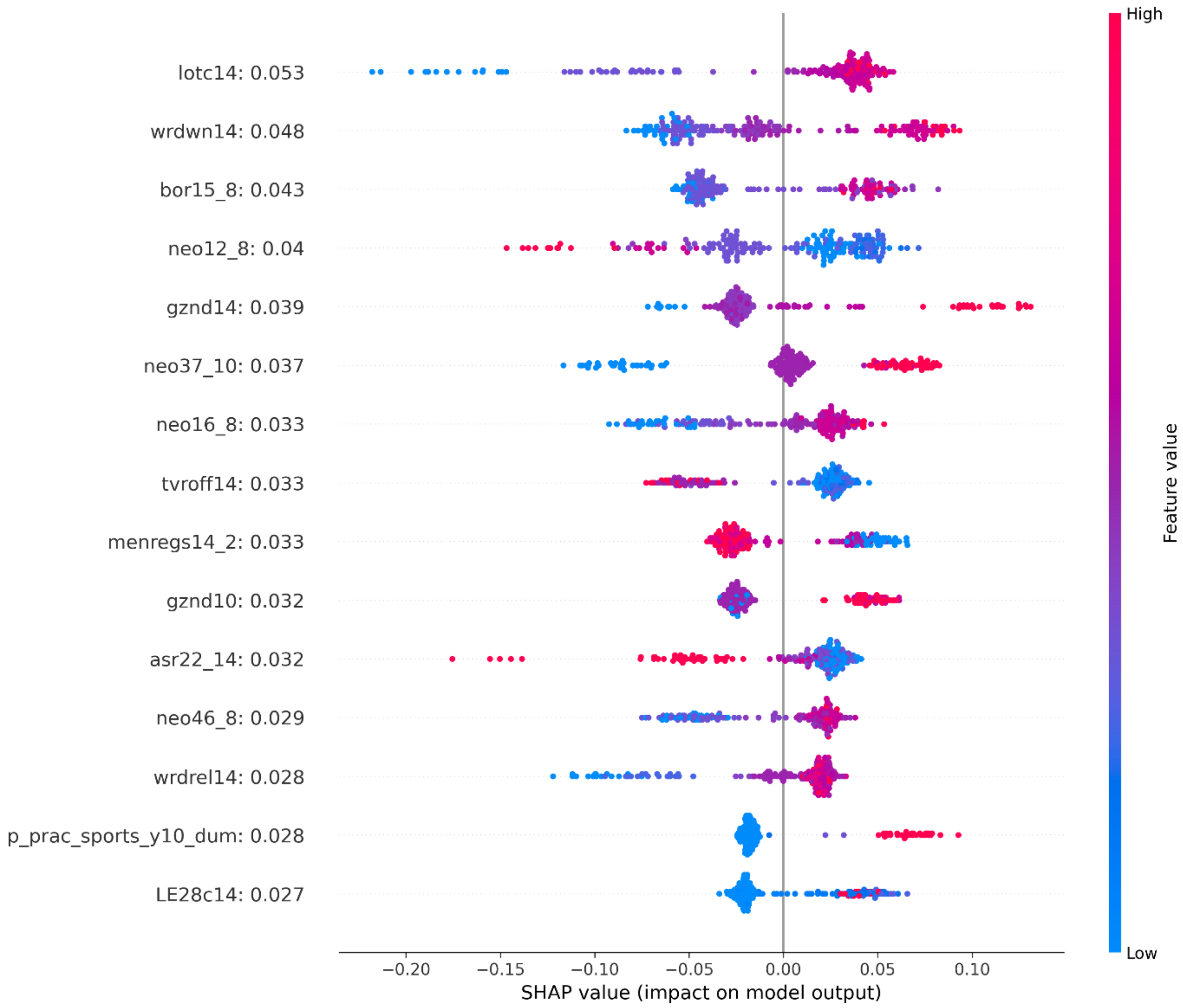
lotc14: 0.053
wrdwn14: 0.048
bor15_8: 0.043
neo12_8: 0.04
gznd14: 0.039
neo37_10: 0.037
neo16_8: 0.033
tvroff14: 0.033
menregs14_2: 0.033
gznd10: 0.032
asr22_14: 0.032
neo46_8: 0.029
wrdrel14: 0.028
p_prac_sports_y10_dum: 0.028
LE28c14: 0.027
−0.20
−0.15
−0.10
−0.05
0.00
0.05
0.10
SHAP value (impact on model output)
High
Low
Feature value

XGB

Multimodal: specific exposome + general exposome

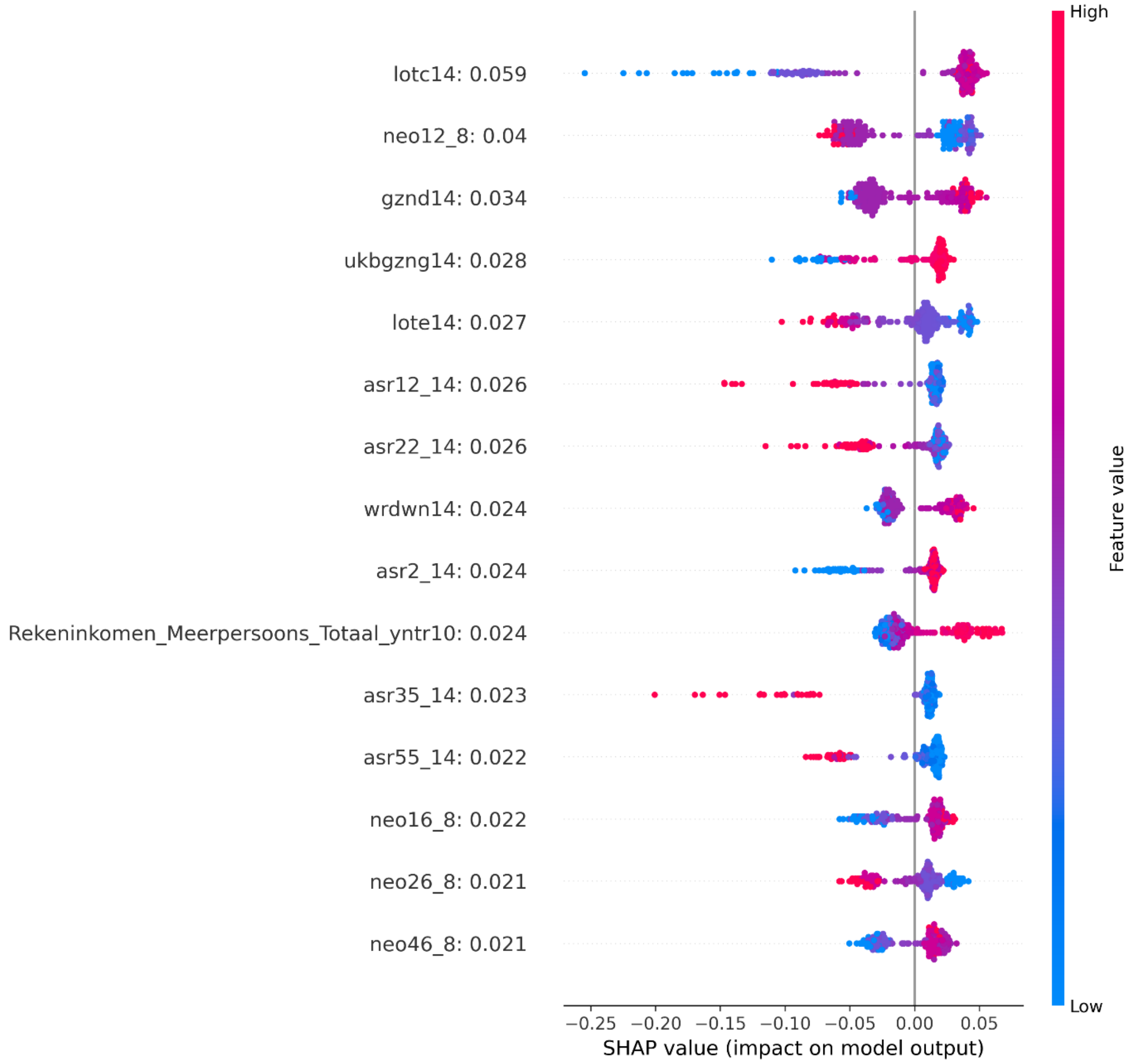

RF

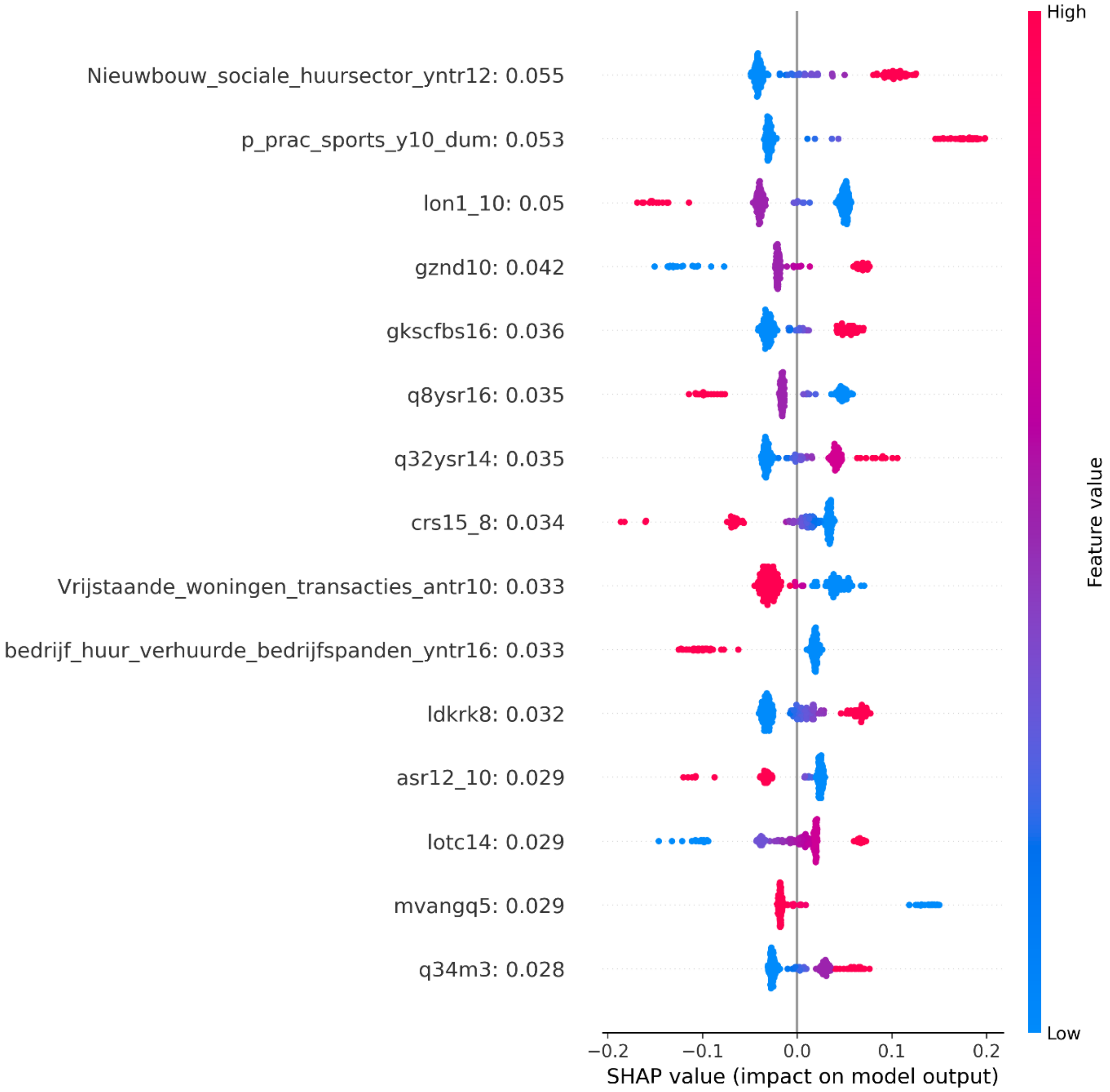
High
Low
Feature value
Nieuwbouw_sociale_huursector_yntr12: 0.055
p_prac_sports_y10_dum: 0.053
lon1_10: 0.05
gznd10: 0.042
gkscfbs16: 0.036
q8ysr16: 0.035
q32ysr14: 0.035
crs15_8: 0.034
Vrijstaande_woningen_transacties_antr10: 0.033
bedrijf_huur_verhuurde_bedrijfspanden_yntr16: 0.033
ldkrk8: 0.032
asr12_10: 0.029
lotc14: 0.029
mvangq5: 0.029
q34m3: 0.028
−0.2
−0.1
0.0
0.1
0.2
SHAP value (impact on model output)

SVM

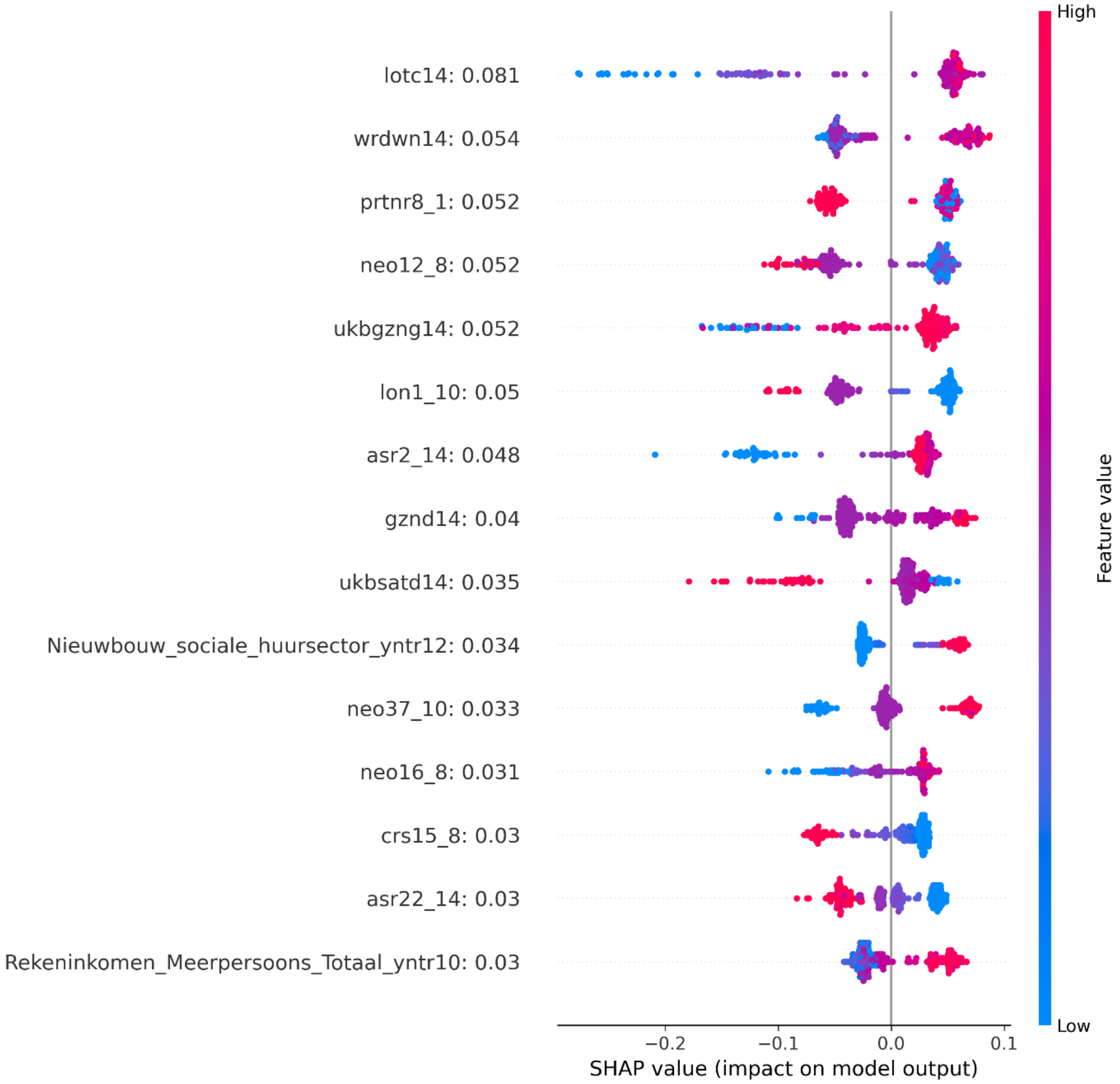
High
lotc14: 0.081
wrdwn14: 0.054
prtnr8_1: 0.052
neo12_8: 0.052
ukbgzng14: 0.052
lon1_10: 0.05
asr2_14: 0.048
gznd14: 0.04
ukbsatd14: 0.035
Nieuwbouw_sociale_huursector_yntr12: 0.034
neo37_10: 0.033
neo16_8: 0.031
crs15_8: 0.03
asr22_14: 0.03
Rekeninkomen_Meerpersoons_Totaal_yntr10: 0.03
Feature value
Low
−0.2
−0.1
0.0
0.1
SHAP value (impact on model output)

XGB

Multimodal: genome + general exposome

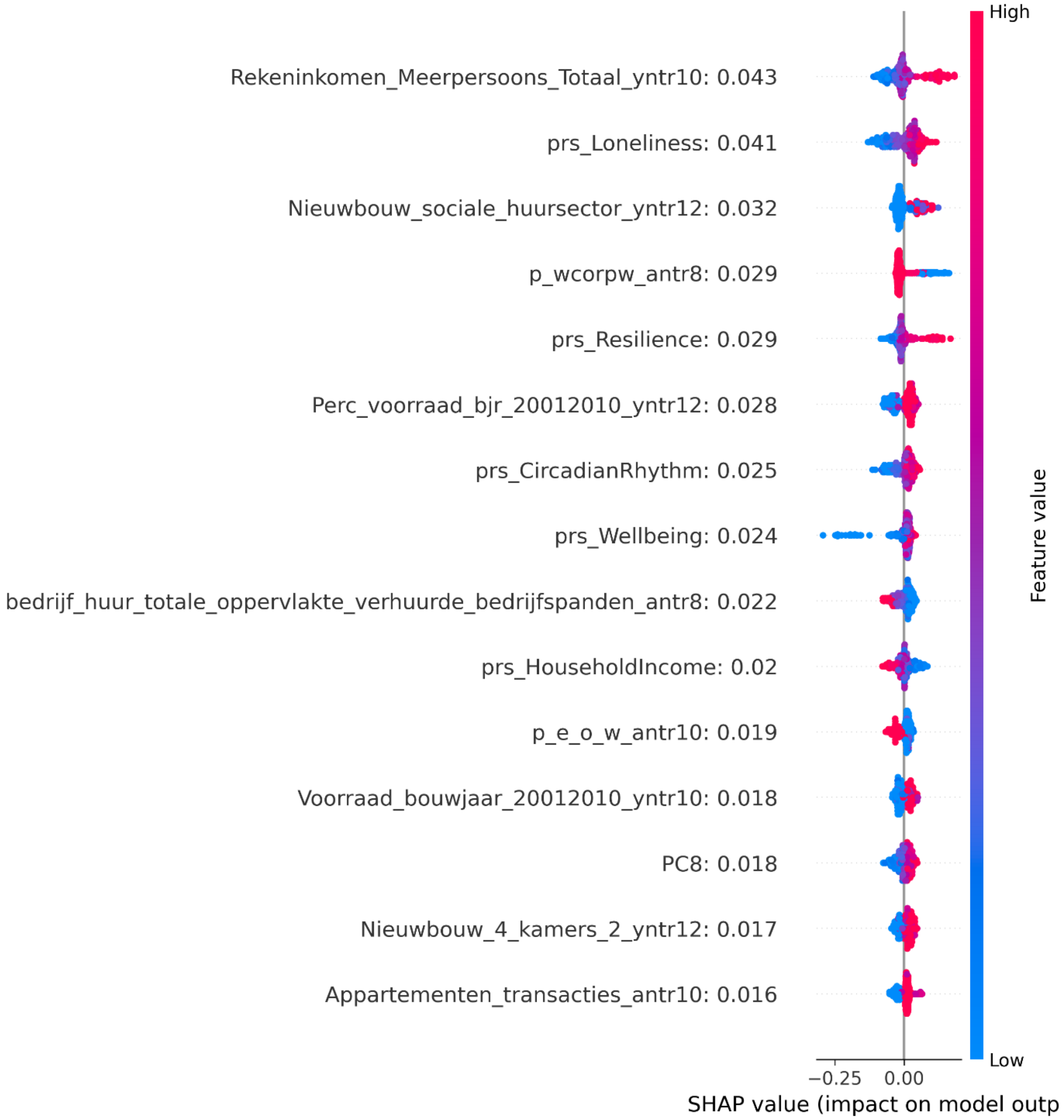

RF

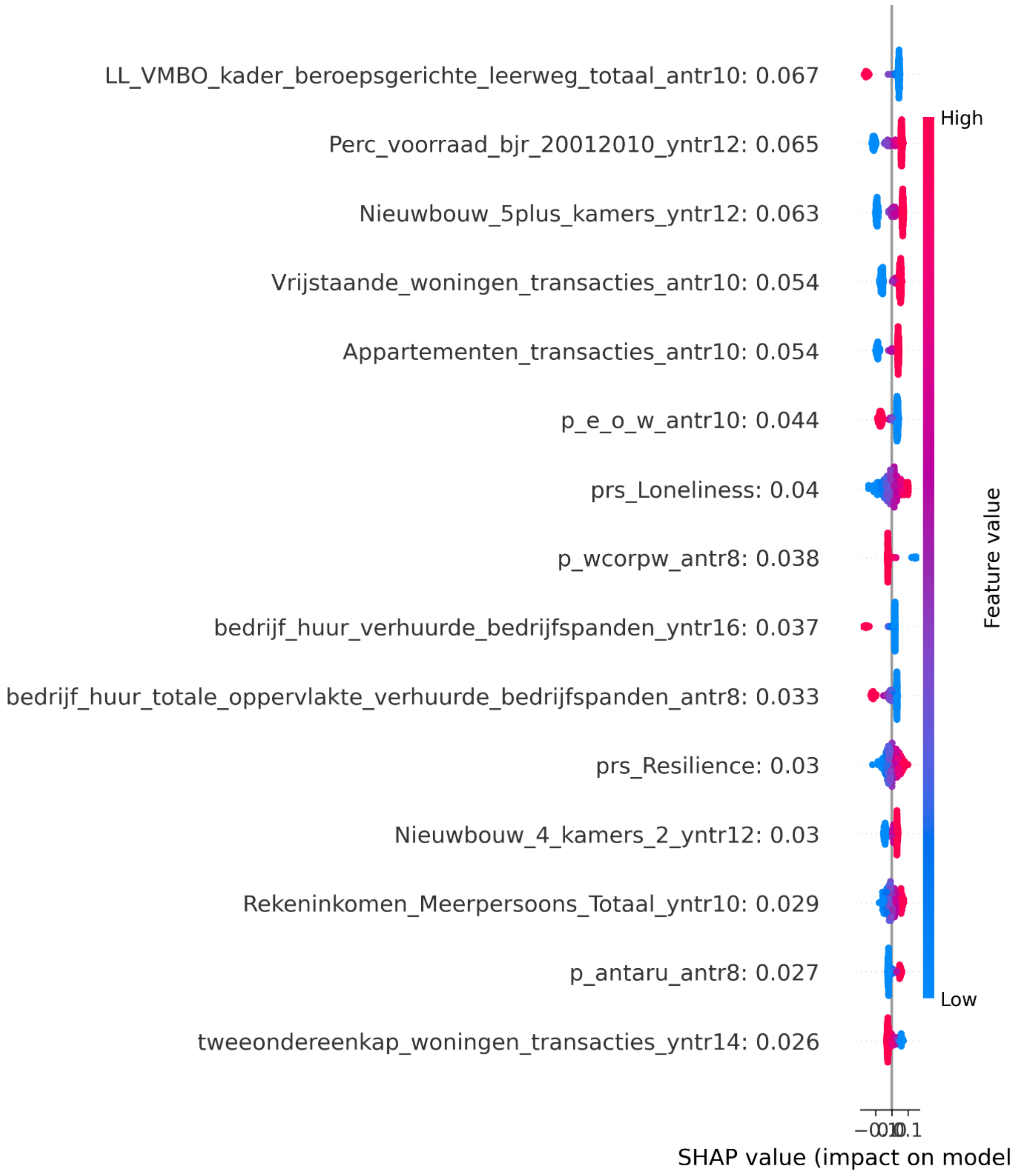

LL_VMBO_kader_beroepsgerichte_leerweg_totaal_antr10: 0.067
Perc_voorraad_bjr_20012010_yntr12: 0.065
Nieuwbouw_5plus_kamers_yntr12: 0.063
Vrijstaande_woningen_transacties_antr10: 0.054
Appartementen_transacties_antr10: 0.054
p_e_o_w_antr10: 0.044
prs_Loneliness: 0.04
p_wcorpw_antr8: 0.038
bedrijf_huur_verhuurde_bedrijfspanden_yntr16: 0.037
bedrijf_huur_totale_oppervlakte_verhuurde_bedrijfspanden_antr8: 0.033
prs_Resilience: 0.03
Nieuwbouw_4_kamers_2_yntr12: 0.03
Rekeninkomen_Meerpersoons_Totaal_yntr10: 0.029
p_antaru_antr8: 0.027
tweeondereenkap_woningen_transacties_yntr14: 0.026
High
Low
Feature value
SHAP value (impact on model

SVM

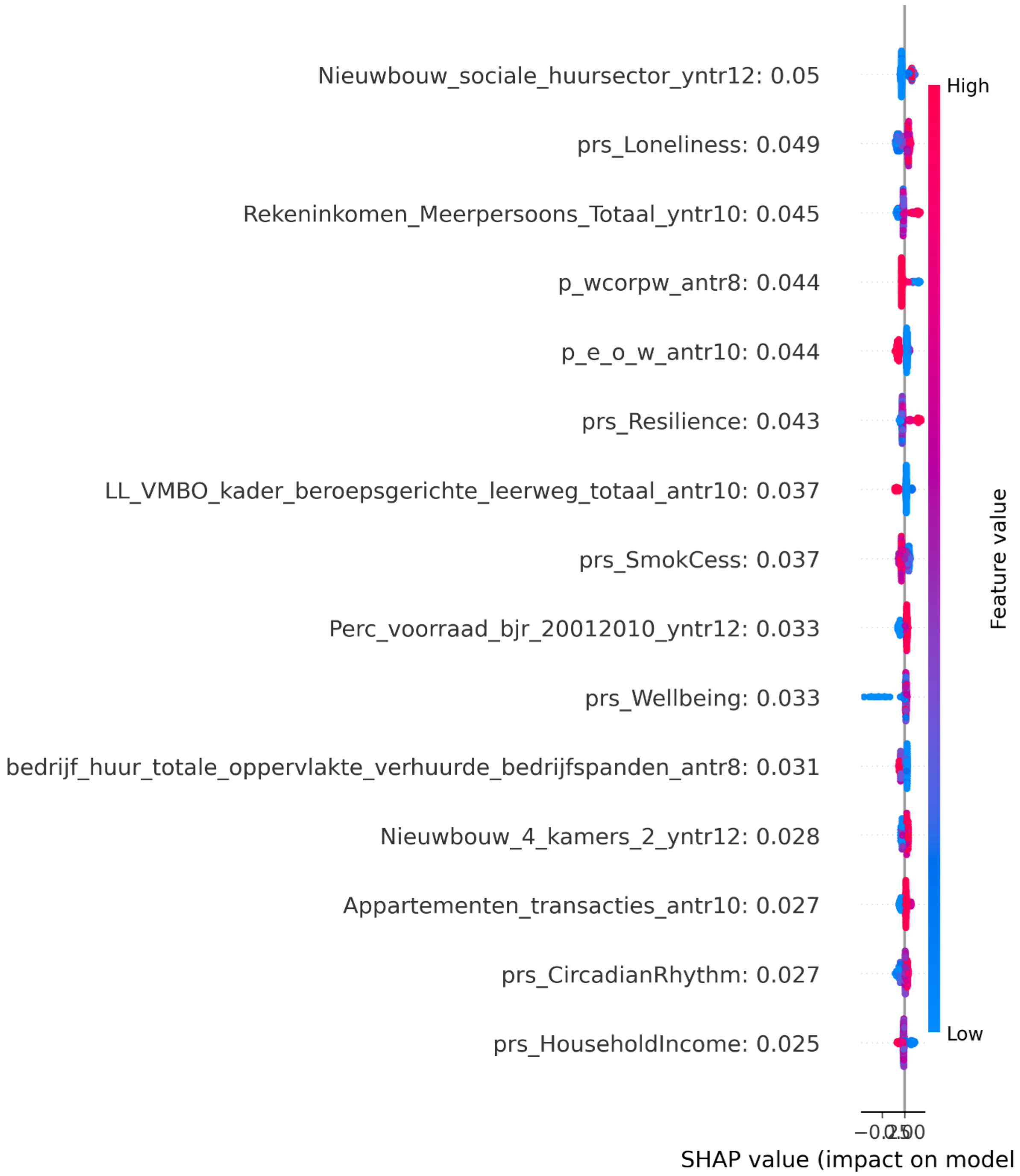

Nieuwbouw_sociale_huursector_yntr12: 0.05
prs_Loneliness: 0.049
Rekeninkomen_Meerpersoons_Totaal_yntr10: 0.045
p_wcorpw_antr8: 0.044
p_e_o_w_antr10: 0.044
prs_Resilience: 0.043
LL_VMBO_kader_beroepsgerichte_leerweg_totaal_antr10: 0.037
prs_SmokCess: 0.037
Perc_voorraad_bjr_20012010_yntr12: 0.033
prs_Wellbeing: 0.033
bedrijf_huur_totale_oppervlakte_verhuurde_bedrijfspanden_antr8: 0.031
Nieuwbouw_4_kamers_2_yntr12: 0.028
Appartementen_transacties_antr10: 0.027
prs_CircadianRhythm: 0.027
prs_HouseholdIncome: 0.025
High
Low
Feature value
SHAP value (impact on model

XGB

Multimodal: specific exposome + genome + general exposome

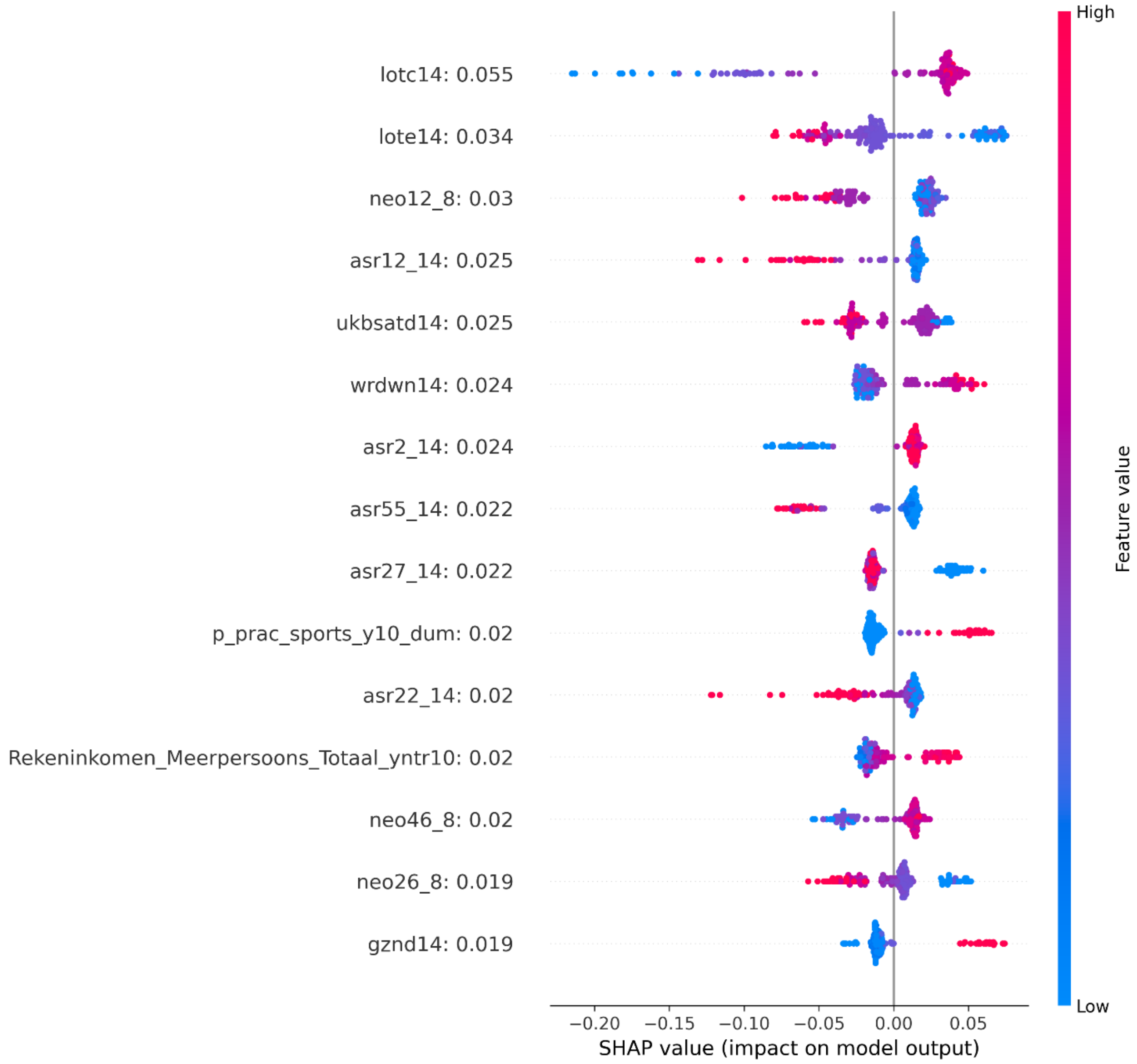

RF

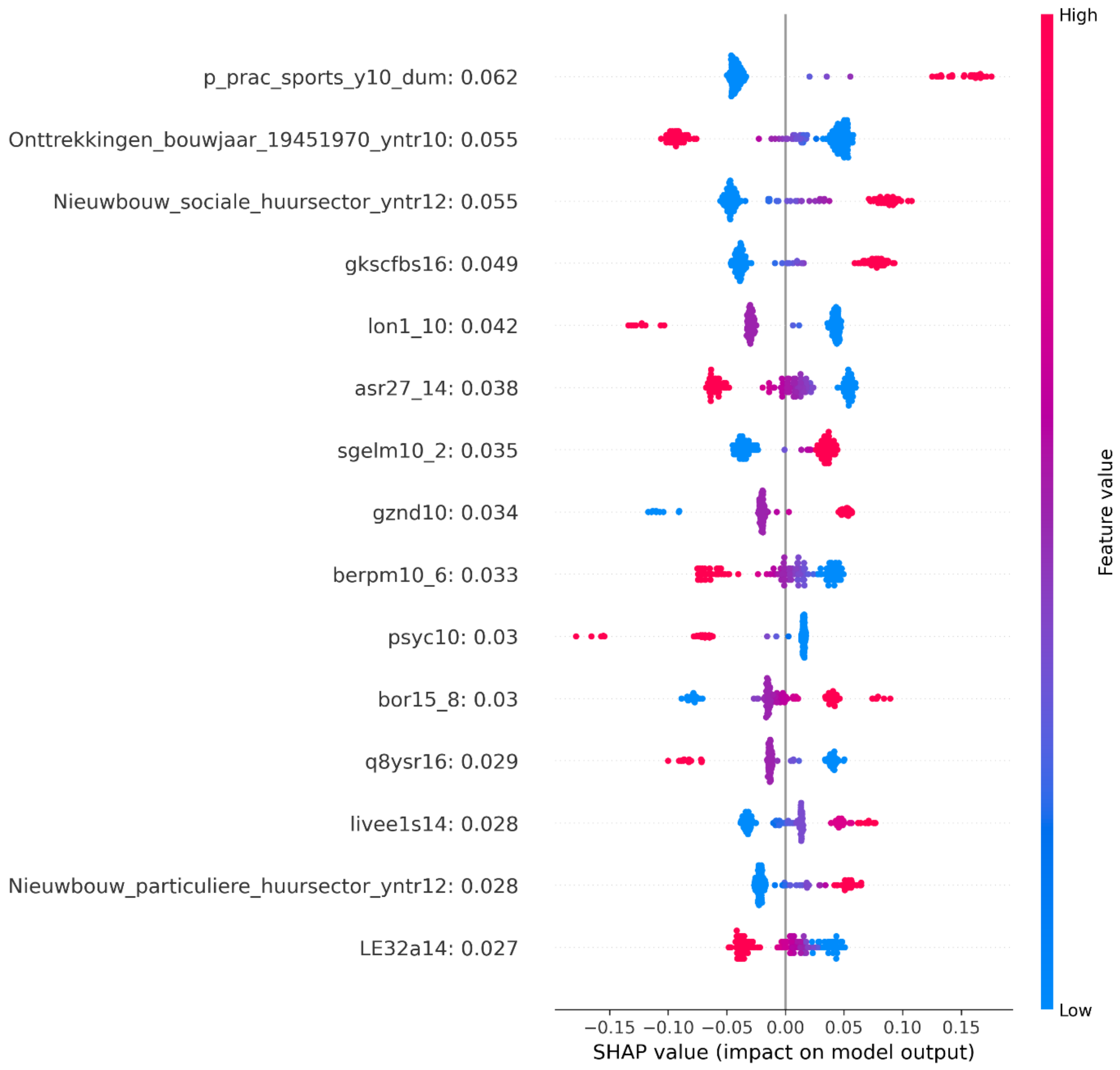
High
p_prac_sports_y10_dum: 0.062
Onttrekkingen_bouwjaar_19451970_yntr10: 0.055
Nieuwbouw_sociale_huursector_yntr12: 0.055
gkscfbs16: 0.049
lon1_10: 0.042
asr27_14: 0.038
sgelm10_2: 0.035
gznd10: 0.034
berpm10_6: 0.033
psyc10: 0.03
bor15_8: 0.03
q8ysr16: 0.029
livee1s14: 0.028
Nieuwbouw_particuliere_huursector_yntr12: 0.028
LE32a14: 0.027
Feature value
Low
−0.15 −0.10 −0.05 0.00 0.05 0.10 0.15
SHAP value (impact on model output)

SVM

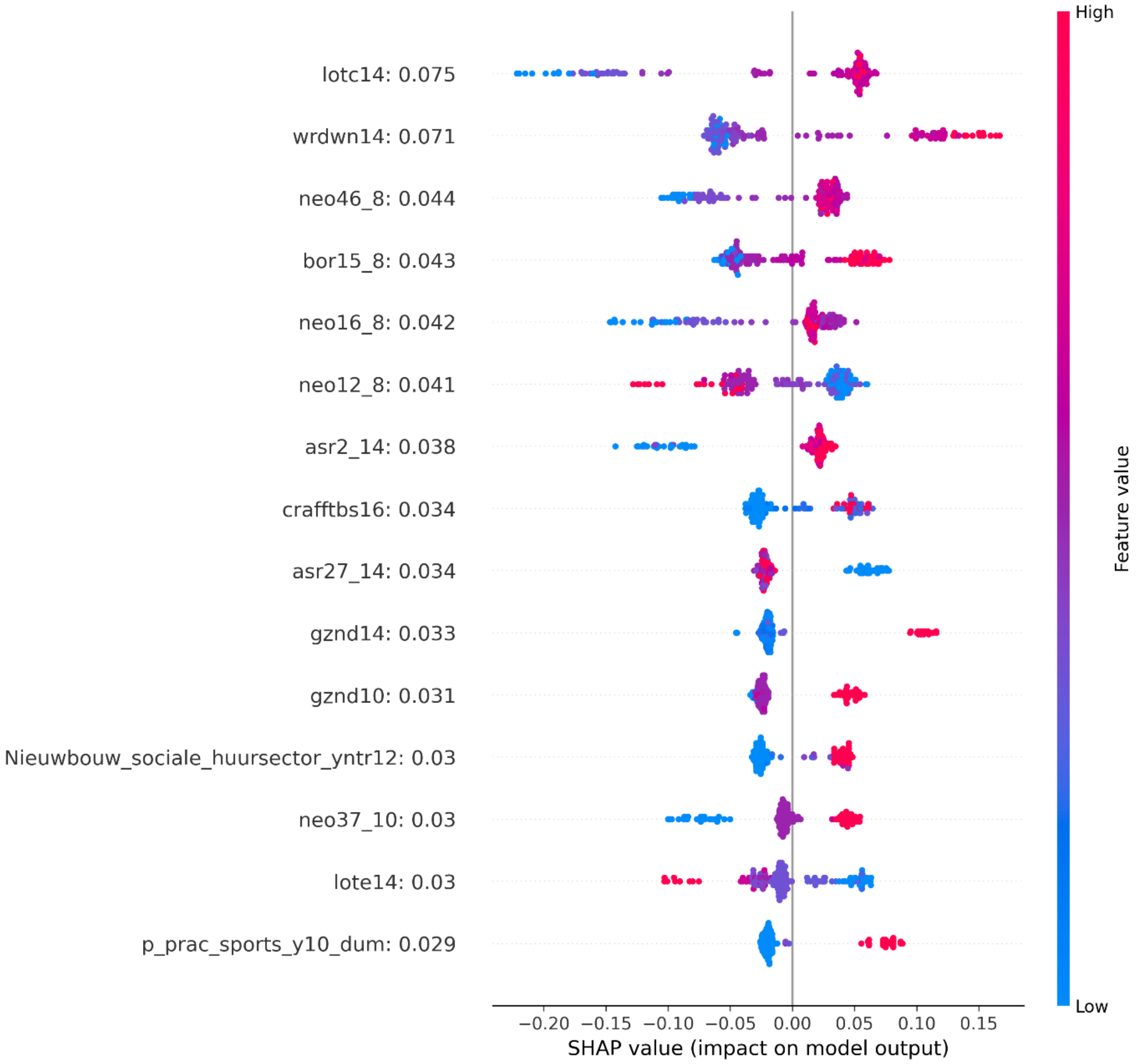
lotc14: 0.075
wrdwn14: 0.071
neo46_8: 0.044
bor15_8: 0.043
neo16_8: 0.042
neo12_8: 0.041
asr2_14: 0.038
crafftbs16: 0.034
asr27_14: 0.034
gznd14: 0.033
gznd10: 0.031
Nieuwbouw_sociale_huursector_yntr12: 0.03
neo37_10: 0.03
lote14: 0.03
p_prac_sports_y10_dum: 0.029
−0.20 −0.15 −0.10 −0.05 0.00 0.05 0.10 0.15
SHAP value (impact on model output)
High
Feature value
Low

XGB

Supplemental Material 2

Sensitivity analyses

A set of sensitivity analyses were conducted to test the robustness of the results from our main analyses. Although tree-based models such as random forest and XGBoost are relatively outlier robust, we investigated the influence of outliers on model performance by repeating all our main analyses (unimodal and multimodal models) after removing multivariate outliers based on Mahalanobis distances (MD). These distances were calculated after estimating a robust feature covariance matrix using the *MinCovDet* function in Python. Observations with squared MD values larger than a threshold value corresponding to a *p*-value of .001 compared to a Chi square distribution were removed, and all unimodal and multimodal models were repeated based on these reduced samples. Model performance was not substantively affected except for the unimodal specific exposome model (*reduction* in R^2 of .181 to .575; Table S8) and the specific + general exposome model (*reduction* in R^2 of .112 to .617). However, given the large number of features on which the covariance matrix was based, observations were (too) easily flagged as being outliers, sometimes reducing our sample sizes by a large factor (e.g., as much as 50% in the unimodal specific exposome model). We therefore repeated the analyses, now simply removing observations belonging to the top 5% MD values. Differences in performance compared to the main analyses were small (maximum increase of .021 for the unimodal specific exposome model, maximum decrease of -.040 unimodal general exposome). Note that the performance after removing outliers largely *decreased* meaning that the 'outliers' likely represented rare but possible and informative cases.

Results of sensitivity analyses specific to different unimodal and multimodal models are reported below.

Unimodal specific exposome

In line with previous work[6], we first excluded features related to mental health before feeding the features into the pipeline. In these analyses, 140 features (out of 1527) were selected, with 49 features (35%) from childhood and adolescence (each contributing at least two features; Table S5). The model based on these features reached the same performance (.701 [.633 - .757]) compared to our previously reported unimodal specific exposome model (.702).

A strong asset of our outcome measure was that it was a wellbeing score based on multiple measurements in adulthood. However, this could have led to differences in performance depending on how many wellbeing measurement waves were present for each individual. The most common combination was having data from all three outcome waves (42%), followed by a combination of ANTR8 and ANTR10 (28%), and a combination of ANTR10 and ANTR14 (13%), with the other combinations being much less frequent. Only 15% of the sample had only a single adult wave on which the outcome was based. We investigated whether the number of outcome measurements influenced our results by comparing the mean squared prediction errors between participants with one (15%), two (43%), or three (42%) outcome measurements. An ANOVA on box-cox transformed squared errors (because of their skewness) showed a difference between the three groups ($p = 4.054\text{x}10^{-4}$). Errors tended to be somewhat larger for those with one single outcome measurement ($M = .333$) compared to two ($M =.108$) or three ($M = .152$) waves, with only the difference between the single outcome and two outcome group being significant ($p = 3.573\text{x}10^{-4}$). However, the single outcome group was relatively small (15% of the sample); although individual differences in reliability of the outcome on model performance may have been present, we can expect the influence to be limited.

In a way, the comparison between the unimodal specific exposome, genome, and general exposome analyses was not entirely fair, given that respectively 2645, 60, and 732

features were available. We tested whether the sheer number of available specific exposome features was responsible for the higher performance of the specific exposome compared to the genome and general exposome. To this end, we randomly sampled 60 features from the full set of 2645 specific exposome features and entered those into the machine learning pipeline, repeating this procedure 5 times. The same process was conducted again, but then taking a random sample of 732 features. When 732 features were included, the average R^2 (over the 5 datasets) was .668 (*SD* = .016), relatively similar to the R^2 based on all features (.702). When 60 features were included the average was .407 (*SD* = .041), a strong reduction in model performance but still much higher compared to the unimodal genome and general exposome performance (R^2 values around zero). Thus, the difference in predictive power between the specific exposome and genome/general exposome models did not appear to be due to the sheer number of available features.

We also investigated the extent to which using a longitudinal outcome influenced our results. A concern with this approach is that prediction performance may be inflated because we are partly predicting back in time; the outcome measure is partly based on measurements (e.g., from ANTR8) that precede some of the features (e.g., from ANTR10 and ANTR14) used to predict the outcome. To test for this, we first created a single wave ANTR14 wellbeing factor score based on the ANTR14 wellbeing measures in a subsample of participants (N_{train} = 678, N_{test} = 171) for which this information was available. As in the main analyses, all features from childhood/adolescence and adulthood were included; because the outcome was based on the last study wave, we are only predicting forward in time (i.e., predictors from YNTR3 to ANTR8) or concurrently (ANTR14). Using this outcome, the predictive accuracy (R^2 = .663, [.546 - .749]) was highly similar to the accuracy the main analyses based on the longitudinal outcome (.701).

Finally, we tested whether the increased predictive power of more proximal features were due to the greater availability of features in more proximal study waves. To this end, we identified the wave with the smallest number of features available (YNTR5, 126 features). Subsequently, we randomly sampled 125 features from all subsequent study waves, and re-trained and evaluated all models based on this smaller subset of features, repeating this procedure three times per study wave. In this way, there was a level playing field in terms of number of predictors for each wave. Based on the results of these analyses (Table S9), the conclusions remained unchanged: the younger childhood waves up until YNTR5 were not predictive of adult wellbeing, model performance around age 7, 10 and 12 were highly similar, with a linear increase from age 14 onwards to adulthood. In general, the R^2 values were slightly attenuated as expected from the smaller number of included features, but the rank order of predictiveness of the study waves remained unchanged.

Unimodal general exposome

Many general exposome features were characterized by distributions with many zeros (for example, the number of swimming pools within a 1km radius usually is zero). We therefore first dichotomized all general exposome features for which the mode was zero and subsequently re-ran our unimodal general exposome models, which are reported in the manuscript. Supplemental Material 3 reports results on non-transformed general exposome features.

Multimodal specific exposome, genome, and general exposome

In our multimodal models including all three modalities, the number of features (212 specific exposome + 28 genotypic (including platform dummies and genetic PCs) + 29 exposures = 269) was larger than the number of people in the test set (146). This may have led to overfitting in the training set, undermining model performance in the test set. To test this, we reduced the number of features by doing a PCA on the specific exposome features,

and extracted 51 principal components which captured at least 65% of the variance of the original features. These 51 independent principal components were entered as features in combination with the 28 genetic and 29 environmental features (a total of 108) in the ML models. The number of principal components was chosen to arrive at a similar features-to-people-in-test set-ratio (108 / 146 = 74%) as in our optimal unimodal specific exposome model (212 / 276 = 77%). For comparison, models were also trained and evaluated based on the 51 principal components alone. Again, in these analyses, adding the genetic and environmental features to the model did not significantly increase model performance (.624 [.481 - .717]) compared to when only the specific exposome features (i.e., the 51 principal components) were included (.634 [.497 - .727]; Δ_{MSE} = .040, Z = .304, p = .761). The lack of incremental prediction thus do not seem to be only due to the overfitting due to the larger number of features compared to the number of participants.

Supplemental Material 3

General exposome: non-dichotomized features

As a sensitivity analysis, because of the skewness of the general exposome features, we dichotomized all general exposome features for which the mode was zero and found that this model showed increased performance. Below, we report results for the "regular" general exposome models without this dichotomization (see also Table S4).

Table S3.1. Model performance general exposome models without dichotomization.

Model/Data set	Optimal model	R^2	LL CI	UL CI
Unimodal general exposome	RF	.036	.009	.059
Specific exposome + general exposome	XGB	.668	.580	.736
Genome + general exposome	XGB	.033	-.028	.083
Specific exposome + genome + general exposome	XGB	.658	.555	.725

Note. RF = Random forest; XGB = extreme gradient boosting; LL = lower limit; UL = upper limit; CI = 95% confidence interval.

Feature	Label	Domain
g_ink_po_yntr16	Average income per person, YNTR_16	Population, Income
p_hh_osm_antr10	Percentage of households around or below social minimum, ANTR_10	Population, Income
Rekeninkomen_Totaal_yntr10	Accounting household income housing benefit receivers (total), YNTR_10	Income
g_ink_po_yntr14	Average income per person, YNTR_14	Population, Income
Nieuwbouw_3_kamers_yntr12	Number of newly built houses (3 rooms), YNTR_12	Housing stock
Vrijstaande_woningen_transacties_antr10	Freestanding housing transactions, ANTR_10	Housing stock
bedrijf_huur_verhuurde_bedrijfspanden_yntr16	Number of rented business premises, YNTR_16	Housing stock, Land use
Rekeninkomen_Aanvrager_65jplus_yntr10	Accounting household income housing benefit receivers (65> years old), YNTR_10	Income
vrz_antr10	Liveability score for amenities, ANTR_10	Liveability, Amenities
Nieuwbouw_4_kamers_2_yntr12	Number of newly built houses (4 rooms), YNTR_12	Housing stock
Onttrekkingen_totaal_yntr12	Number of residential property conversions, YNTR_12	Housing stock
Leerlingen_voortgezet_onderwijs_vrouwen_yntr12	Pupils secondary school (female), YNTR_12	Population, Education
Rekeninkomen_Aanvrager_23_64j_yntr10	Accounting household income housing benefit receivers (23-64 years old), YNTR_10	Income
Rekeninkomen_Meerpersoons_Totaal_yntr10	Accounting multi-person household income housing benefit receivers, YNTR_10	Income
Perc_leerlingen_basisonderwijs_lft_12_jaar_antr10	Percentage elementary shool pupils age 12, ANTR_10	Population, Education

Feature	Label	Domain
Rekeninkomen_Aanvrager_65jplus_yntr10	Accounting household income housing benefit receivers (65> years old), YNTR_10	Income
Nieuwbouw_3_kamers_yntr12	Number of newly built houses (3 rooms), YNTR_12	Housing stock
vrz_antr10	Liveability score for amenities, ANTR_10	Liveability, Amenities
Rekeninkomen_Aanvrager_23_64j_yntr10	Accounting household income housing benefit receivers (23-64 years old), YNTR_10	Income
g_ink_po_yntr16	Average income per person, YNTR_16	Population, Income
Rekeninkomen_Totaal_yntr10	Accounting household income housing benefit receivers (total), YNTR_10	Income
Binnenzwembad_antr10	Swimming pools (absolute), ANTR_10	Amenities
Hoekwoningen_transacties_yntr12	Corner house transactions, YNTR_12	Housing stock
p_hh_osm_antr10	Percentage of households around or below social minimum, ANTR_10	Population, Income
Nieuwbouw_4_kamers_2_yntr12	Number of newly built houses (4 rooms), YNTR_12	Housing stock
Totaal_bioscopen_en_filmtheaters_per_100.000_inwoners_antr10	Cinemas (relative), ANTR_10	Amenities
Nieuwbouw_meergezinswoningen_yntr12	Number of newly built houses (multi-person household homes), YNTR_12	Housing stock
Rekeninkomen_Meerpersoons_Totaal_yntr10	Accounting multi-person household income housing benefit receivers, YNTR_10	Income
LL_VMBO_kader_beroepsgerichte_leerweg_totaal_antr10	Pre-vocational education pupils ("kader"), ANTR_10	Population, Education
p_stadsv_antr8	District heating (relative), ANTR_8	Housing stock

Figure S3.1. SHAP (top) and permutation importance values (bottom) of top 15 general exposome features across all three machine learning models (extreme gradient boosting, support vector machine, and random forest).

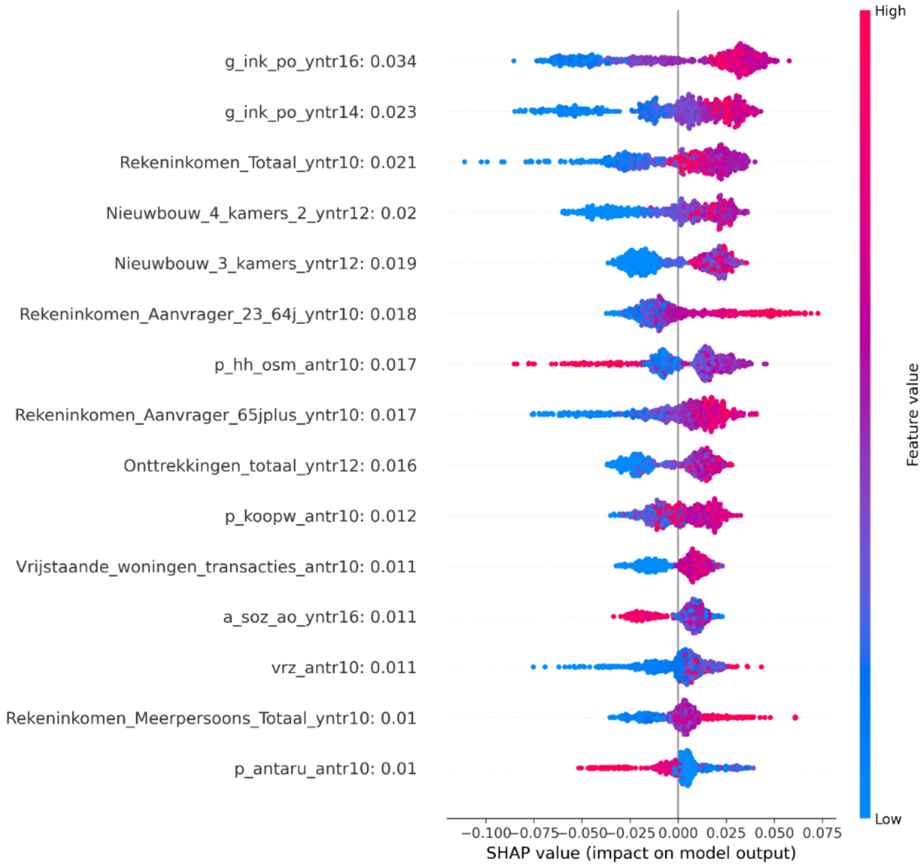

Figure S3.2. SHapley Additive exPlanation (SHAP) values of top 15 (non-dichotomized) general exposome features based on optimal random forest model.

As found for the dichotomized general exposome models, adding the “regular” general exposome features did not improve models beyond the specific exposome, either independently (.668 [.578 - .737] vs. .688 [.598 - .755]) or jointly with the genomic predictors (.658 [.556 - .726]) vs. .672 [.571 - .740]).

Specific exposome: including wellbeing indicators in childhood and adolescence

In the manuscript, we report models in which direct wellbeing (i.e., outcome) indicators in childhood and adolescence were removed. Below are the R^2-values for models including these features. In general, the unimodal specific exposome model had a somewhat higher R^2-

values compared to the model without these features (.702), while the models based on multiple data modalities performed somewhat worse. But overall, model performance was highly similar.

Table S3.2. Model performance specific exposome models including wellbeing indicators in childhood and adolescence.

					Specific exposome only
Model/Data set	Best model	R^2	LL CI	UL CI	R^2
Unimodal specific exposome	XGB	.722	.661	.771	-
Specific exposome + general exposome	XGB	.676	.591	.740	.700
Specific exposome + genome	XGB	.621	.516	.695	.642
Specific exposome + genome + general exposome	XGB	.587	.435	.686	.620

Note. RF = Random forest; XGB = extreme gradient boosting; LL = lower limit; UL = upper limit; CI = 95% confidence interval.

As can be expected based on the longitudinal stability of wellbeing, previous wellbeing levels from adolescence (*welbbs16, welbes16, kwleefs16*) were predictive of wellbeing in adulthood (Figure S3.3 and S3.4).

Feature	Label	Domain
lotc14	optimistic about future, ANTR_14	Optimism
p_prac_sports_y10_dum	Practice any sports no/yes (Parent), YNTR_10	Exercise
neo37_10	cheerful/vivacious, ANTR_10	Psychological, Personality
neo12_8	not cheerful/light-hearted, ANTR_8	Psychological, Personality
neo16_8	lonely/blue, ANTR_8	Psychological, Personality
bor5_10	feel empty, ANTR_10	Mental health, Borderline
lote14	rarely count on good things, ANTR_14	Optimism
lon1_10	lack companionship, ANTR_10	Loneliness
welbbs16	Wellbeing - My living conditions are excellent, YNTR_16	Mental health
LE28c14	Life events item 28: Had a child - 1-5 years ago, ANTR_14	Life events
gznd10	How would you rate your general health?, ANTR_10	Health
wrdwn14	Rating living situation (0-10), ANTR_14	Living situation, SES
welbes16	Wellbeing - If I had to live my life again, I would do more or less the same, YNTR_16	Mental health
prtnr8	Steady relationship (yes/no), ANTR_8	Social relations
bor11_10	PAI-BOR 11 - I often wonder what I should do with my life, ANTR_10	Mental health, Borderline

Feature	Label	Domain
p_prac_sports_y10_dum	Practice any sports no/yes (Parent), YNTR_10	Exercise
lotc14	optimistic about future, ANTR_14	Optimism
neo37_10	cheerful/vivacious, ANTR_10	Psychological, Personality
neo12_8	not cheerful/light-hearted, ANTR_8	Psychological, Personality
asr35_14	worthless, ANTR_14	Mental health, Psychopathology
lote14	rarely count on good things, ANTR_14	Optimism
bor5_10	feel empty, ANTR_10	Mental health, Borderline
kwleefs16	Where on the bar would you say your life generally stands?, YNTR_16	Mental health
neo16_8	lonely/blue, ANTR_8	Psychological, Personality
gznd14	How would you rate your general health?, ANTR_14	Health
lon1_10	lack companionship, ANTR_10	Loneliness
neo46_8	NEO item 46: I am seldom sad or depressed., ANTR_8	Psychological, Personality
prtnr8	Steady relationship (yes/no), ANTR_8	Social relations
crs15_8	underachiever, ANTR_8	ADHD
asr12_10	feel lonely, ANTR_10	Mental health, Psychopathology

Figure S3.3. SHAP (top) and permutation importance values (bottom) of top 15 specific exposome features (models including child and adolescence wellbeing indicators) across all three machine learning models (extreme gradient boosting, support vector machine, and random forest).

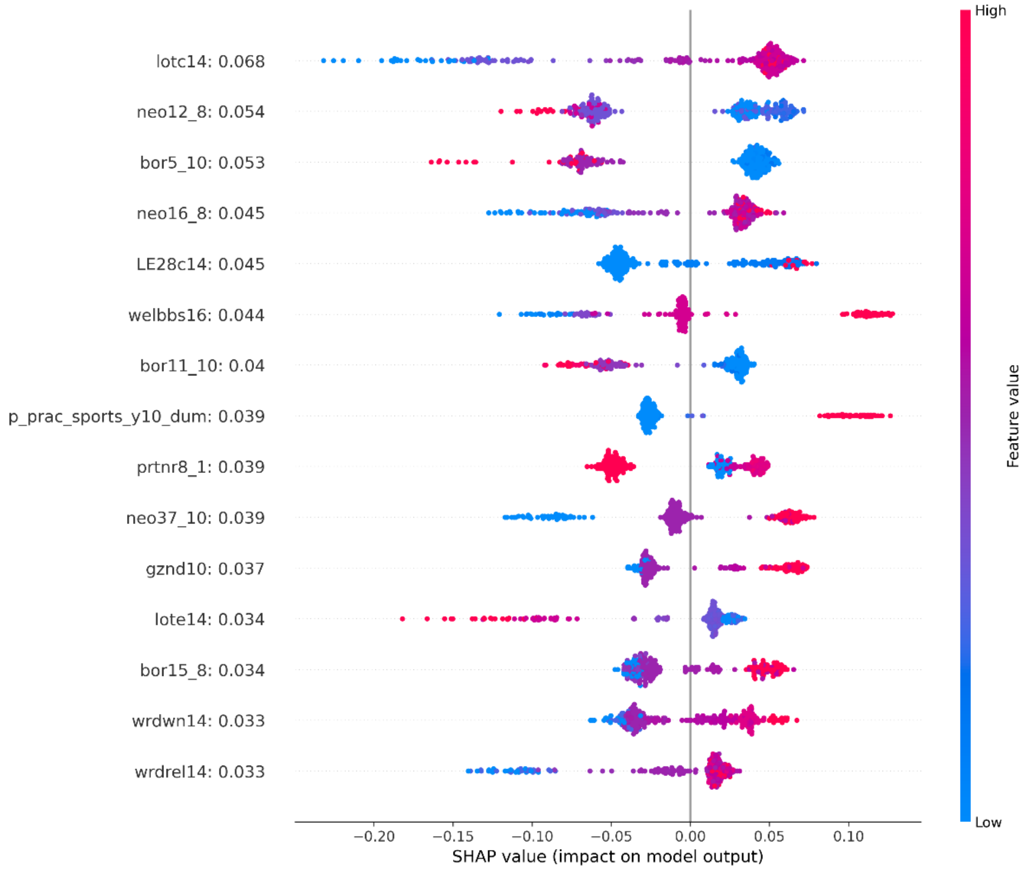

Figure S3.4. SHapley Additive exPlanation (SHAP) values of top 15 specific exposome features from models including child/adolescence wellbeing features, based on optimal extreme gradient boost model.
