# Supplementary material for: Building machine learning prediction models for well-being using predictors from the exposome and genome in a population cohort
Source: Nat Ment Health. 2024 Aug 14;2(10):1217–30. doi: 10.1038/s44220-024-00294-2 (PMC11511667; doi:10.1038/s44220-024-00294-2)
Supplement: Supplementary file 2 — Reporting Summary [file 44220_2024_294_MOESM2_ESM.pdf]

nature portfolio

Corresponding author(s): Dirk Pelt

Last updated by author(s): May 7, 2024

Reporting Summary

Nature Portfolio wishes to improve the reproducibility of the work that we publish. This form provides structure for consistency and transparency in reporting. For further information on Nature Portfolio policies, see our Editorial Policies and the Editorial Policy Checklist.

Statistics

For all statistical analyses, confirm that the following items are present in the figure legend, table legend, main text, or Methods section.

n/a	Confirmed	
☐	☒	The exact sample size (*n*) for each experimental group/condition, given as a discrete number and unit of measurement
☐	☒	A statement on whether measurements were taken from distinct samples or whether the same sample was measured repeatedly
☐	☒	The statistical test(s) used AND whether they are one- or two-sided<br>*Only common tests should be described solely by name; describe more complex techniques in the Methods section.*
☐	☒	A description of all covariates tested
☐	☒	A description of any assumptions or corrections, such as tests of normality and adjustment for multiple comparisons
☐	☒	A full description of the statistical parameters including central tendency (e.g. means) or other basic estimates (e.g. regression coefficient) AND variation (e.g. standard deviation) or associated estimates of uncertainty (e.g. confidence intervals)
☐	☒	For null hypothesis testing, the test statistic (e.g. F, t, r) with confidence intervals, effect sizes, degrees of freedom and P value noted<br>*Give P values as exact values whenever suitable.*
☒	☐	For Bayesian analysis, information on the choice of priors and Markov chain Monte Carlo settings
☒	☐	For hierarchical and complex designs, identification of the appropriate level for tests and full reporting of outcomes
☐	☒	Estimates of effect sizes (e.g. Cohen's d, Pearson's r), indicating how they were calculated

Our web collection on statistics for biologists contains articles on many of the points above.

Software and code

Policy information about availability of computer code

Data collection	In more recent cohorts, data was collected through online survey tools (Survalyzer, NetQ).
Data analysis	All pre-processing steps were conducted in the R environment (version 4.0.5), packages heavily used were tidyverse (2.0.0), lavaan (0.6-15), e1071 (1.7-13), and fastDummies (1.6.3). The figures were made using ggplot2 (3.4.3). LDpred 0.9 and plink2 were used for the construction of the polygenic scores. The machine learning analyses were conducted in Python using PyCharm in combination with Anaconda3, most notable with the scikit-learn (1.3.0) and XGBoost (1.7.6) packages. See https://osf.io/zphw8/ for Python code including all loaded packages.

For manuscripts utilizing custom algorithms or software that are central to the research but not yet described in published literature, software must be made available to editors and reviewers. We strongly encourage code deposition in a community repository (e.g. GitHub). See the Nature Portfolio guidelines for submitting code & software for further information.

Data

Policy information about availability of data

All manuscripts must include a data availability statement. This statement should provide the following information, where applicable:

- Accession codes, unique identifiers, or web links for publicly available datasets
- A description of any restrictions on data availability
- For clinical datasets or third party data, please ensure that the statement adheres to our policy

Being part of a national prospective cohort study, Netherlands Twin Register data cannot be made publicly available for privacy reasons, but they are available for

legitimate researchers via the data access procedure (https://tweelingenregister.vu.nl/information_for_researchers/working-with-ntr-data). Data of the Geoscience and health cohort consortium (GECCO) can be requested through the data access request form (https://www.gecco.nl/exposure-data-1/).

Research involving human participants, their data, or biological material

Policy information about studies with human participants or human data. See also policy information about sex, gender (identity/presentation), and sexual orientation and race, ethnicity and racism.

Reporting on sex and gender	In childhood, parent's reported on their child's gender by checking a box after the statements "Gender of oldest twin" ("Geslacht oudste van de tweeling") and "Gender of youngest twin" ("Geslacht oudste van de tweeling"), with the boxes labeled "boy" ("jongen") and "girl" ("meisje"). In adulthood, participants checked a box next to the statement "Gender" ("Geslacht"), with labels "male" ("man") and "female" ("vrouw"). These variables were entered as features in our machine learning models: gender specific analyses were not conducted because we were interested in building wellbeing prediction models for the entire population.
Reporting on race, ethnicity, or other socially relevant groupings	Our phenotypic set of variables included questions on the birth country of the participants and the birth country of their parents. In all our models including genetic predictors (polygenic scores), we included the first ten principal components to correct for population stratification.
Population characteristics	See below.
Recruitment	At the start of the NTR, a commercial 'birth felicitation' bureau that visited parents of newborns at home and through city councils. Additional recruitment is done with the support of the Dutch Society of Parentsof Multiples (Nederlandse Vereniging van Ouders van Meerlingen: NVOM; https://www.nvom.nl), through (online) newsletters, and events.<br><br>For more information on NTR recruitment, see: 10.1375/twin.5.5.401, 10.1017/thg.2019.93, 10.1017/thg.2012.118
Ethics oversight	All procedures performed in studies involving human participants were in accordance with the ethical standards of the institutional and/or national research committee and with the 1964 Helsinki declaration. Data collection was approved by the Central Ethics Committee on Research Involving Human Subjects of the University Medical Centers Amsterdam. Signed informed consent was obtained from all individual participants included in the study. Only participants who consented to record linkage were included. Participants were not compensated for participation.

Note that full information on the approval of the study protocol must also be provided in the manuscript.

Field-specific reporting

Please select the one below that is the best fit for your research. If you are not sure, read the appropriate sections before making your selection.

☐ Life sciences ☒ Behavioural & social sciences ☐ Ecological, evolutionary & environmental sciences

For a reference copy of the document with all sections, see nature.com/documents/nr-reporting-summary-flat.pdf

Behavioural & social sciences study design

All studies must disclose on these points even when the disclosure is negative.

Study description	This study involves quantitative, longitudinal survey and genetic data (polygenic scores) of voluntary participants of the Netherlands Twin Register collected between 1991 and 2022, linked with environmental exposures at the postal code level provided by the Geoscience and hEalth Cohort COnsortium (GECCO).
Research sample	The Netherlands Twin Register is a population-based cohort established in 1986. The NTR is a population-wide, non-clinical sample and distinguishes between the Young NTR (YNTR) and the Adult NTR (ANTR). The YNTR comprises of children rated by their parents, and standardized surveys are sent out around ages 1, 2, 3, 5, 7, 9, and 12. For several years (2004 - 2014), YNTR twins aged 14, 16, and 18 were invited for a self-report survey study. When participants in the YNTR reach the age of 18 years they are invited to take part in the ANTR. The ANTR consists of adult participants providing self-reports. The NTR collects DNA in their biobank. The sample was chosen for availability of wellbeing data, psychosocial data, genetic data, and possibility of record linkage. Gender distributions varied across the different datasets but were predominantly female [range 66%-73%]. Age ranged between 18 and 77. Participants were mostly higher educated. Full demographic information is reported in Table 1. The sample is not fully representative.
Sampling strategy	Sample size was determined based on availability of data. As pre-registered, we requested all NTR variables with sample sizes N>3000 to ensure a large enough initial sample size for each variable. For child data, only mother-reports were requested to maximize sample size. The NTR is not a random sample of the Dutch population (more female and higher educated participants, see also Table S2 on attrition). Rather, in the NTR, purposive sampling is used to recruit multiples and their family members. Once participants are recruited, sampling is based on availability. For the genetic data, criterion-based sampling is used: sufficient phenotypic (i.e., survey) data needs to be available before becoming eligible for genetic data collection.
Data collection	In the ANTR, self-reports were paper and pencil until ~2009 (ANTR8), when surveys were partly distributed online as a pilot. Since then, data collection has gradually moved to fully online (present). In the YNTR, the surveys were paper and pencil until ~2016, after which parents filled in surveys about their children online. No researcher was present during any of the data collection. Since all data

	has been collected before the design of this study, all researchers involved with data collection were blind to the study hypotheses.
Timing	The childhood waves were collected from 01/90 until present (periodically), the adolescent waves between 05/04 and 12/13. The adult waves were collected between 01/09 and 12/12 (ANTR8), between 05/13 and 12/15 (ANTR10), and between 06/19 and 02/20 (ANTR14). For more information on NTR study waves, see: 10.1017/thg.2019.93, 10.1017/thg.2012.118
Data exclusions	For the outcome variable (wellbeing), we excluded participants younger than 18 years old (N = 129) because we were interested in predicting adult wellbeing. Those with only one single wellbeing measurement available were also excluded (N = 1,094). Participants and features with more than 55% missing values were excluded (Unimodal specific exposome: Np = 19,365, Nf = 2,968; Unimodal genome: Np = 14,895; Unimodal general exposome: Np = 16,910, Nf = 5,287). We pre-registered 50% but this would have reduced our sample sizes too much.
Non-participation	In general, the response rate in the NTR is estimated to be between 40% and 50% (see 10.1017/thg.2012.140 and 10.1017/thg.2012.118).
Randomization	This study did not involve random assignment of participants to experimental groups. All important covariates (age and gender) were included in the analyses.

Reporting for specific materials, systems and methods

We require information from authors about some types of materials, experimental systems and methods used in many studies. Here, indicate whether each material, system or method listed is relevant to your study. If you are not sure if a list item applies to your research, read the appropriate section before selecting a response.

Materials & experimental systems

n/a	Involved in the study	
☒	☐	Antibodies
☒	☐	Eukaryotic cell lines
☒	☐	Palaeontology and archaeology
☒	☐	Animals and other organisms
☒	☐	Clinical data
☒	☐	Dual use research of concern
☒	☐	Plants

Methods

n/a	Involved in the study	
☒	☐	ChIP-seq
☒	☐	Flow cytometry
☒	☐	MRI-based neuroimaging
